# Insights into lentil diversity, domestication, and the genetic basis of important agronomic traits through resequencing of 238 *Lens* accessions

**DOI:** 10.1111/tpj.70908

**Published:** 2026-05-14

**Authors:** Yu Ma, Dorrie Main, Clarice J. Coyne, Lyndon D. Porter, Garett C. Heineck, Mary Burrows, Marilyn L. Warburton, Renan Uhdre, Hatice Sari, Britton Bourland, Kirstin Bett, Rebecca J. McGee

**Affiliations:** ^1^ Department of Horticulture and Crop Science Ohio State University Columbus Ohio USA; ^2^ Department of Horticulture Washington State University Pullman WA USA; ^3^ Department of Crop and Soil Sciences Washington State University Pullman WA USA; ^4^ USDA‐ARS Plant Germplasm Introduction and Testing Unit Washington State University Pullman WA USA; ^5^ USDA‐ARS Grain Legume Genetics and Physiology Research Unit Prosser WA USA; ^6^ USDA‐ARS Northwest Sustainable Agroecosystems Research Unit Prosser WA USA; ^7^ Virginia Agricultural Experiment Station Virginia Tech Blacksburg Virginia USA; ^8^ Department of Plant Sciences University of Saskatchewan Saskatoon Saskatchewan Canada; ^9^ USDA‐ARS Grain Legume Genetics and Physiology Research Unit Pullman WA USA

**Keywords:** *Lens culinaris*, *Lens orientalis*, *Lens odemensis*, *Lens ervoides*, *Lens nigricans*, WGS, SNP, population diversity, GWAS

## Abstract

Lentil is an important legume crop with significant nutritional value, playing a pivotal role in environmentally sustainable agricultural systems. The lentil whole‐genome resequencing data have not been reported. In this study, we re‐sequenced a total of 238 *Lens* accessions, including 112 cultivated lentils, 71 landraces, and 55 wild species, and generated a comprehensive map of lentil genome variation with 103 290 296 single‐nucleotide polymorphisms (SNPs), 9 099 509 indels, and 276 171 copy number variations (CNVs). The highest numbers of variants were observed in wild species overall. Population genomic analysis revealed lentil was first domesticated in the Near East. Among wild species, *L. orientalis* showed the closest relationship with cultivated lentils. Demographic history analysis demonstrated the divergence time between *L. orientalis* and *L. culinaris* was around 12 Kya. Scans for selective sweeps indicated traits including flowering time and disease resistance might have been under continuous selection during domestication. The genetic architecture of *Fusarium* root rot resistance and other economically and agronomically important traits were also identified. The two genes encoding a toll‐interleukin receptor nucleotide‐binding site leucine‐rich repeat (TIR‐NBS‐LRR) protein and an auxin‐binding protein were predicted as candidate genes associated with *Fusarium* root rot resistance. This study provides valuable genomic resources for both basic and applied efforts to understand and exploit the genetic basis of important traits for lentil crop improvement via molecular breeding.

## INTRODUCTION

Cultivated lentil (*Lens culinaris* Medik. subsp. *culinaris*, 2*n* = 2*x* = 14) is an important grain legume worldwide. It is increasingly used as a dietary source of protein, carbohydrates, minerals, and fiber. As an integral rotation component of cereal‐based cropping systems, it contributes to agricultural and environmental sustainability by enabling the management of pesticide and herbicide residues, and by fixing atmospheric nitrogen (Stefaniak & McPhee, 2015). The genus *Lens* are cool‐season legumes, belonging to the family Fabaceae. There have been debates about the taxonomy of *Lens* as morphological, hybridization, cytogenetics, and molecular studies have provided conflicting results (Abo‐Elwafa et al., 1995; Ahmad et al., 1996; Ahmad & McNeil, 1996; Fratini & Ruiz, 2006; Havey & Muehlbauer, 1989; Koul et al., 2017; Mayer & Bagga, 2002; Mayer & Soltis, 1994; Sharma et al., 1995; Sharma et al., 1996; Sonnante et al., 2003; Suvorova, 2014). The most accepted classification recognizes four species with seven taxa, including cultivated *L. culinaris* subsp. *culinaris* and six wild *L. culinaris* Medik. subsp. *orientalis* (Boiss.) Ponert, *L. culinaris* Medik. subsp. *tomentosus* (Ladiz.) *L*. odemensis Ladiz., *L. lamottei* Czefr., *L. ervoides* (Brign.) Grande, and *L. nigricans* (M. Bieb.) Webb & Berthel. (Ferguson et al., 2000; Koul et al., 2017; Liber et al., 2021; Wong et al., 2015).

Lentil was one of the first domesticated crops (Zohary & Hopf, 1973). However, domestication history of cultivated lentils remains largely unknown. It has been indicated *L. culinaris* subsp. *orientalis* (henceforth referred to as *L. orientalis*) is the closest wild progenitor of *L. culinaris* (Daniel Zohary, 1972). Wild lentil relatives are widely distributed throughout the Mediterranean Basin and co‐occur in the Aegean area and southwestern Turkey (Guerra‐García et al., 2021). Cross‐compatibility levels differ between cultivated *L. culinaris* and different wild lentil relatives. *L. orientalis* and *L. odemensis* can be crossed with *L. culinaris* (Coyne et al., 2020). However, due to hybrid embryo breakdown, successful crosses between *L. ervoides* and *L. nigricans* and cultivated species have been difficult to achieve (Abbo & Ladizinsky, 1991; Abbo & Ladizinsky, 1994; Fratini & Ruiz, 2006).

Next‐generation sequencing technology has revolutionized agriculture science and proven to be powerful for understanding population structure, evolution and domestication, and genetic mechanisms underlying important traits. Several Quantitative Trait Loci (QTL) associated with *Fusarium* root rot (FRR) resistance (Heineck et al., 2022) and other important agronomic traits (Fedoruk, 2013; Khazaei et al., 2018; Kumar, Gupta, et al., 2018; Neupane et al., 2023; Rajendran et al., 2021; Singh et al., 2019) have been reported using genome‐wide association study (GWAS) in lentil. However, resolution has been limited by low‐density markers, making it difficult to identify plausible candidate genes. In addition, domestication history and population structure remain largely unknown. In this study, we resequenced the genomes of 238 *Lens* accessions, comprising cultivated and wild relative species. We constructed a comprehensive variation map with 103 million single‐nucleotide polymorphisms (SNPs), 9 million indels, and 276 171 copy number variations (CNVs), from which we analyzed phylogenetic relationship and population diversity, supporting the previously proposed center of origin in the Near East. Introgression regions and genetic architecture underlying FRR resistance and 13 agronomic traits were identified. Results from this study provide insights into the evolutionary history of lentil and will enable important advances in lentil improvement and breeding programs.

## RESULTS

### Sequencing and variation discovery

We resequenced 238 cultivated and wild *Len* accessions (Table S1) and generated a total of 10.16 Tb of whole‐genome sequencing (WGS) raw data with 67.26 billion paired‐end reads (Table S2). Average sequence depth of mapped clean reads was 10.19‐fold, ranging from 7.33‐fold to 15.02‐fold. Genome coverage varied among *L. culinaris* cultivated (87.00%), *L. culinaris* landraces (85.15%), *L. orientalis* (81.01%), *L. odemensis* (62.79%), *L. ervoides* (53.16%), and *L. nigricans* (37.52%) (Figure S1).

We identified 103 290 296 SNPs, 9 099 509 indels (≤5 bp), and 276 171 CNVs across all lentil accessions (Table 1). Of 103 million SNPs, 5.30% were located in genes and 1.52% were in coding regions (Table 1 and Table S3). The average non‐synonymous to synonymous ratio for SNPs was 1.25 (Table S3), which is comparable to ratios reported in chickpea (1.20) (Varshney et al., 2019), rice (1.29) (Xu et al., 2012), and pigeon pea (1.18) (Varshney et al., 2017). In addition, 255 462 homozygous SNPs and 1 007 850 heterozygous SNPs were identified for the reference genotype, CDC Redberry. Due to the high likelihood of being a variant calling error for these homozygous SNPs, the error rate was estimated at approximately 0.25%. Of 9 million indels (1–5 bp), 3 797 018 were insertions and 5 302 491 were deletions (Table 1). 86.20% of indels were found in intergenic regions and 0.67% were in coding regions where 76.94% could cause frameshift mutations (Tables S4 and S5). Of 276 171 CNVs, 199 940 were duplicated CNVs and 76 231 were deleted CNVs (Table 1). The number of SNPs and indels across each accession is summarized in Tables S6 and S7.

**Table 1:** Summary of variants identified in the 238 lentil accessions

Variant	Type	Cultivated	Landrace	Wild	*Lens orientalis*	*Lens odemensis*	*Lens ervoides*	*Lens nigricans*	All genotypes
	Accessions (*n*)^a^	112	71	55	18	4	28	5	238
SNP^b^	Total	49 397 557	50 828 966	89 363 556	63 960 606	11 620 577	36 707 230	16 369 193	103 290 296
	Exons	570 592	586 606	1 443 179	890 585	207 361	565 551	531 107	1 572 887
	Introns	1 410 782	1 456 932	3 553 422	2 227 046	411 070	1 328 569	1 158 395	3 901 458
	Intergenic	45 686 614	46 987 228	79 840 915	57 969 757	10 511 698	33 112 892	13 337 146	92 877 537
Indel	Total	3 448 232	3 586 974	7 714 721	4 150 670	793 582	3 390 692	1 388 570	9 099 509
	Insertions (1–5 bp)^c^	1 408 539	1 466 441	3 250 363	1 707 545	323 449	1 442 266	625 570	3 797 018
	Deletions (1–5 bp)^c^	2 039 693	2 120 533	4 464 358	2 443 125	470 133	1 948 426	763 000	5 302 491
CNV^d^	Total	168 400	166 736	272 393	187 378	114 544	200 942	147 542	276 171
	Deletions	70 119	69 303	75 957	71 754	37 499	66 452	44 771	76 231
	Duplications	98 281	97 433	196 436	115 624	77 045	134 490	102 771	199 940

^a^ *n* indicates numbers of accessions.

^b^ SNP stands for single nucleotide polymorphism.

^c^ bp stands for base pair.

^d^ CNV represents copy number variation.

**Figure 1:**
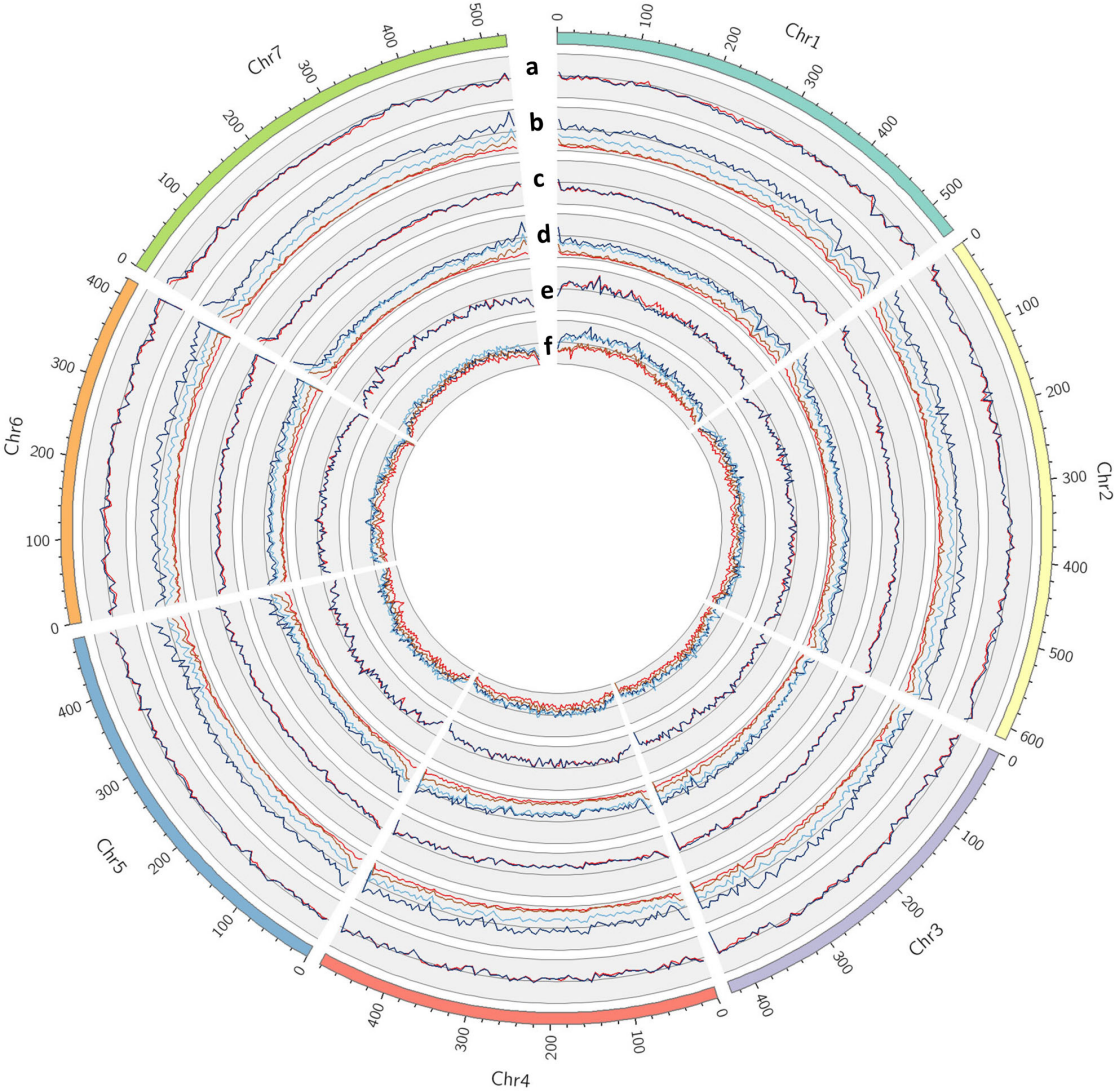
Circos graph representing genome‐wide variants among 238 lentil accessions. (a) SNP density in 5 Mb windows for cultivated (red) and landrace (blue). (b) SNP density in 5 Mb windows for *L. orientalis* (dark blue), *L. odemensis* (red), *L. ervoides* (light blue), and *L. nigricans* (brown). (c) Indel density in 5 Mb windows for cultivated (red) and landrace (blue). (d) Indel density in 5 Mb windows for *L. orientalis* (dark blue), *L. odemensis* (red), *L. ervoides* (light blue), and *L. nigricans* (brown). (e) CNV density in 5 Mb windows for cultivated (red) and landrace (blue). (f) CNV density in 5 Mb windows for *L. orientalis* (dark blue), *L. odemensis* (red), *L. ervoides* (light blue), and *L. nigricans* (brown). The SNP axis ranges from 0 to 160 000. The indel axis ranges from 0 to 150 000. The CNV axis ranges from 0 to 600. The diagram was drawn using Circos (http://circos.ca).

The abundance of variants varied among different groups (Figure 1). The numbers of observed variants were much higher in wild species than *L. culinaris* overall (Table 1). Compared with the two lentil groups in *L. culinaris*, the abundance of SNPs and indels was slightly higher in landraces than in cultivated lentils, while the abundance of CNVs was lower. The wild species, *L. orientalis*, had the highest number of variants, and *L. odemensis* had the lowest (Table 1). A total of 815 427 SNPs and 23 540 indels were identified in common between the six lentil groups (Figures S2 and S3). Among wild species, the highest numbers of unique SNPs and indels were observed in *L. orientalis* and *L. ervoides*, respectively (Figures S2 and S3). In addition, a large portion of SNPs identified in landraces and cultivated lentils was shared with *L. orientalis* (Figures S2 and S3). The variants discovered here provide new and valuable genetic and genomic resources to assist lentil crop improvement and new cultivar development.

### Population structure of *Lens* accessions

**Figure 2:**
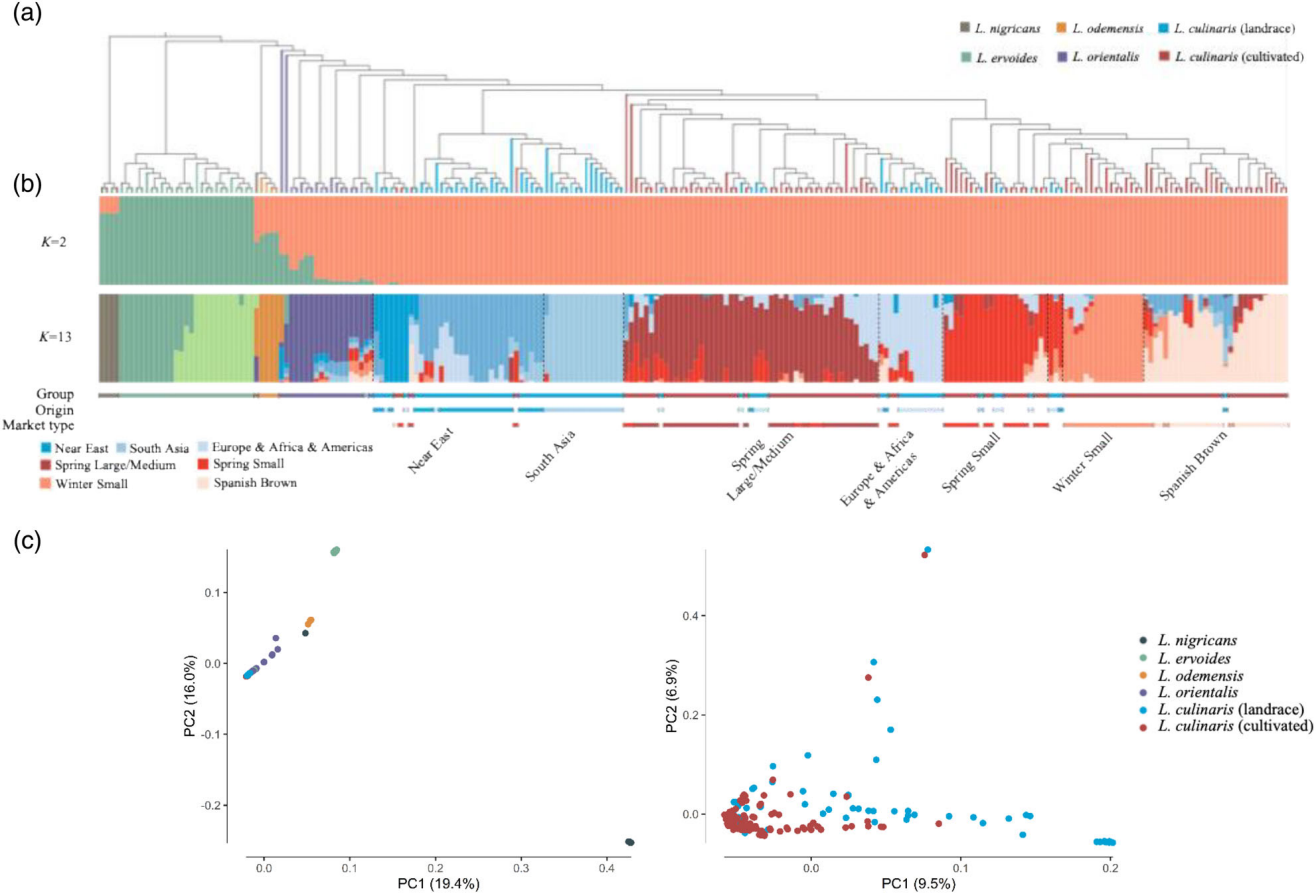
Phylogeny and population structure of 238 lentil accessions. (a) A neighbor‐joining tree constructed using 379 235 SNPs. (b) Population structure analysis with different numbers of kinship (*K* = 2 or 13). Group names are indicated by the first colored horizontal bar; Geographic origins of landraces are indicated by the second colored bar; Market types of cultivated lentils are indicated by the third colored bar. (c) PCA plots of the first two principal components for all 238 lentil accessions (left) and for *L. culinaris* accessions (right).

Phylogenetic relationships among the 238 lentil accessions were explored using 379 235 SNPs at fourfold degenerate sites. The neighbor‐joining phylogenetic analysis with 500 bootstraps revealed monophyletic clades for wild and domesticated lentils (Figure 2a). The phylogenetic relationship of five lentil species (Figure S4) was consistent with previously reported phylogeny and gene pool classification (Wong et al., 2015). Among wild species, *L. orientalis* showed the closest relationship with *L. culinaris* (Figure 2a), supporting the hypothesis that *L. orientalis* is the wild progenitor of *L. culinaris* (Daniel Zohary, 1972). This was consistent with the result of the model‐based clustering analysis when *K* = 2 (Figure 2b). The cross‐validation error analysis showed optimum clustering was estimated at *K* = 13 (Figure S5). When *K* = 13, landraces were subdivided into three clusters including accessions from the Near East, from South Asia, and from Europe, Africa, and Americas together, reflecting spatial genetic variation (Figure 2b). Furthermore, accessions from the Near East were sisters to the other landrace clusters, thus supporting the hypothesis that lentil domestication has likely taken place in the Near East (Daniel Zohary, 1972). In addition, modern Spring Large/Medium, Spring Small, Winter Small, and Spanish Brown market types formed four clusters within cultivated lentils (Figure 2b). In both the phylogeny and population structure analyses (Figure 2a,b), *L. culinaris* accessions were mainly divided into landrace and cultivated subgroups. It was noteworthy that most landraces that originated from Europe, Africa, and Americas were assigned into the cultivated subgroups. The admixture between these landraces and cultivated lentils could be derived from the close phylogenetic relationship and shared pedigree among these accessions, as well as frequent uses of diverse landraces as parents in developing cultivated lentils. The results from phylogenetic and population analysis were further illuminated by principal component analysis (PCA) (Figure 2c and Figure S6). Taxonomic discrepancies were detected in one *L. nigricans* accession, one *L. ervoides* accession and one *L. orientalis* accession (Figure 2a,b, Table S8). Seven landrace accessions and fourteen cultivated accessions were clustered into unexpected groups. These admixed accessions were therefore excluded from downstream analysis (Table S8).

Nucleotide diversity (π) was estimated for each lentil group. *L. orientalis* (3.23 × 10^−3^) showed the greatest diversity among all groups. In *L. culinaris*, landrace (2.48 × 10^−3^) had higher nucleotide diversity than cultivated accessions (1.85 × 10^−3^), suggesting a bottleneck (π_landrace_/ π_cultivated_ = 1.34) occurred during the improvement of cultivated lentils (Table S9). The linkage disequilibrium (LD) decay was measured as the physical distance where the correlation coefficient dropped to half of its maximum value. *L. nigricans* and *L. odemensis* were not included in the LD decay analysis due to the limited sample size. *L. ervoides* has the most rapid LD decay (3.3 kb at *r* ^*2*^ = 0.288). The LD decay rate was slower in *L. culinaris* landrace (39.5 kb at *r* ^*2*^ = 0.281) and *L. culinaris* cultivated accessions (83.6 kb at *r* ^*2*^ = 0.335) than *L. orientalis* (15.0 kb at *r* ^*2*^ = 0.163) (Figure S7). This study found that the LD decay in *L. culinaris* landraces was substantially smaller than was previously reported (Ma et al., 2020) where fewer molecular markers were used. The LD decay in *L. culinaris* cultivated accessions was similar to pigeon pea (*Cajanus cajan*, 70 kb) (Varshney et al., 2017) and *indica* rice (*Oryza sativa*, ~75 kb) (Mather et al., 2007), but slower than sorghum (*Sorghum bicolor*, 19.7 kb) (Mace et al., 2013), and more rapid than common bean (*Phaseolus vulgaris*, 107 kb) (Wu et al., 2020), soybean (*Glycine max*, 150 kb) (Zhou et al., 2015) and chickpea (*Cicer arietinum*, 320 kb) (Varshney et al., 2019). The LD decay between different phylogeographic groups and market type groups was estimated (Figure S7). The fixation index (*F*_ST_) was estimated between two lentil groups to determine the level of population differentiation. *L. culinaris* cultivated accessions showed the closest genetic relationship with *L. culinaris* landrace (*F*_ST_ = 0.0609), followed by *L. orientalis* (*F*_ST_ = 0.2160) and furthest genetic relationship with *L. nigricans* (*F*_ST_ = 0.4265) (Table S10).

### Center of origin of *L. culinaris*

**Figure 3:**
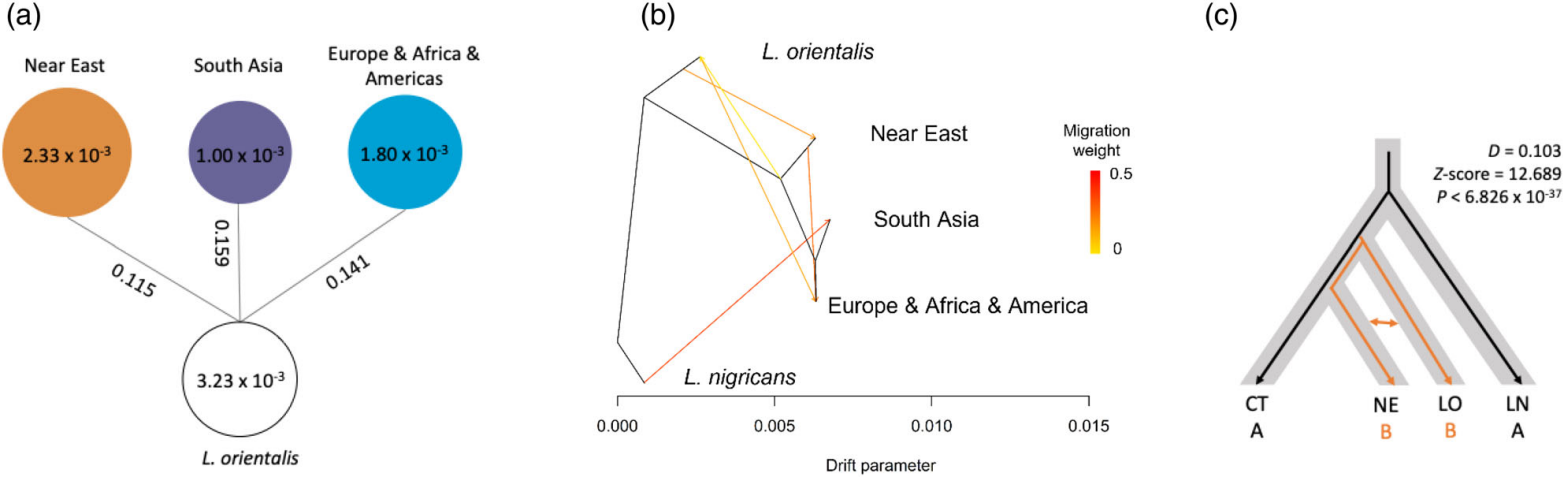
Proposed center of origin of *L. culinaris*. (a) Nucleotide diversity (π) of three phylogeographic groups of *L. culinaris* landrace and fixation index (*F*_ST_) between each group and *L. orientalis*. The π values are represented in each circle, while *F*_ST_ values are represented on each line between groups. (b) Detection of gene flow among the three phylogeographic groups of *L. culinaris* landrace and *L. orientalis* using TreeMix. *L. nigricans* was used as an outgroup. Arrows represent the direction of gene flow. The red color of the line indicates a strong migration while the yellow color shows a weak migration. The scale bar indicates 10 times the average standard error of the entries in the sample covariance matrix. (c) ABBA‐BABA analysis of *L. culinaris* cultivated (CT), *L. culinaris* landrace Near East group (NE), *L. orientalis* (LO), constructed using *L. nigricans* (LN) as an outgroup.

The *L. culinaris* landrace was divided into three phylogeographic groups: Near East, South Asia, and Europe, Africa, and Americas (Figure 2b and Figure S6c). To investigate their relationship with *L. orientalis*, we estimated nucleotide diversities (π) for these three groups and the fixation index (*F*_ST_) between each group and *L. orientalis*. The Near East group had the smallest *F*_ST_ (0.115) compared to South Asia (0.159) and Europe, Africa, and Americas (0.141) (Figure 3a), indicating the Near East is the closest group to *L. orientalis*. In addition, the Near East group had the highest nucleotide diversities (π = 2.33 × 10^−3^) (Figure 3a) and the most rapid LD decay (Figure S7b), consistent with Vavilov's theory that the center of origin has great crop genetic diversity (Vavilov, 1992). Subsequently, we explored gene flow between the three phylogeographic groups and *L. orientalis* using TreeMix. With *L. nigricans* as an outgroup, we detected relatively strong gene flow from *L. orientalis* to the Near East group and the Europe, Africa, and Americas group. A significant migration event was identified from the Near East group into the Europe, Africa, and Americas group (Figure 3b). This gene flow observation was also supported by the ABBA‐BABA test (Figure 3c). These findings further confirm that the Near East was the center of origin for lentil, consistent with the earlier hypothesis (Daniel Zohary, 1972).

### Demographic history inference

**Figure 4:**
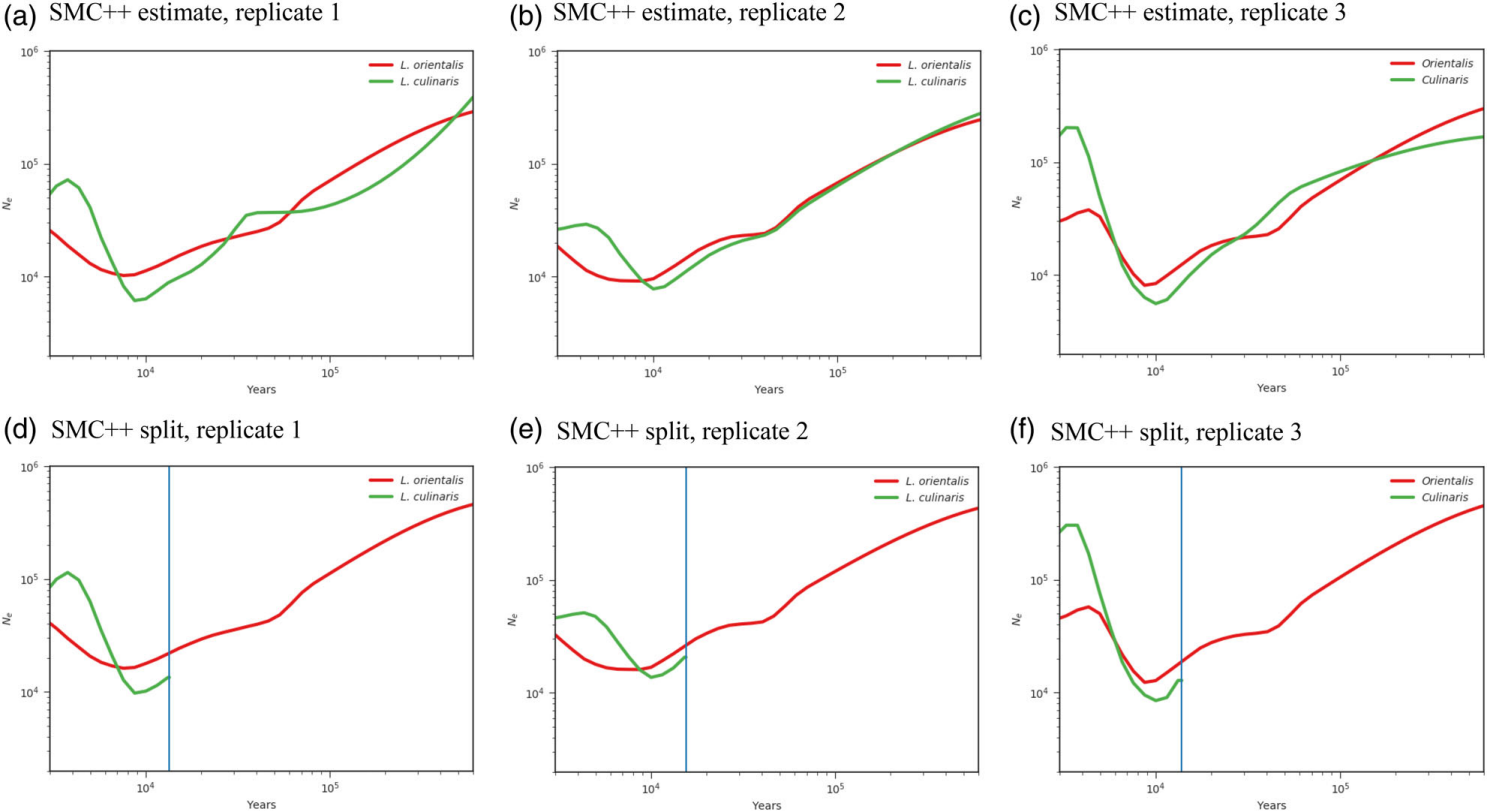
Demographic history of *L. orientalis* and *L. culinaris*. (a–c) Change of effective population size (*Ne*) in *L. orientalis* and *L. culinaris* inferred by a SMC++ estimate analysis with three replicates. (d–f) Divergence between *L. orientalis* and *L. culinaris* inferred by a SMC++ split analysis with three replicates. The SMC++ estimate and SMC++ split analyses were performed on the same three sets of randomly selected 10 samples from each species. The generation time was set as 1 year and the mutation rate per site per generation as 8.3 × 10^−9^.

The demographic history has been characterized in many crops, such as maize (Beissinger et al., 2016; Wang, Beissinger, et al., 2017), African rice (Cubry et al., 2018; Meyer et al., 2016), lettuce (Wei et al., 2021), and grapevine (Liang et al., 2019). However, little demographic analysis has been reported in lentil. We estimated the domestication time by investigating the change of effective population size (*Ne*) of *L. orientalis* and *L. culinaris*. Results showed these two species experienced a steady decline of *Ne* from 200 000–300 000 at 500 Kya to 6000–10 000 at 9 Kya (Figure 4a–c). The *Ne* contraction in *L. culinaris* during this period was relatively stronger than in *L. orientalis*. The subsequent *Ne* expansion was observed around 3 Kya to 10 Kya in both species (Figure 4a–c). The divergence time between *L. orientalis* and *L. culinaris* was estimated to be around 12 Kya (Figure 4d–f). In addition, the three phylogeographic groups of *L. culinaris* landrace, and the four market type groups of *L. culinaris* cultivated accessions, showed a similar partern of *Ne* contraction and expasion to *L. culinaris* (Figure S8).

### Human selection of domestication traits in lentil

**Figure 5:**
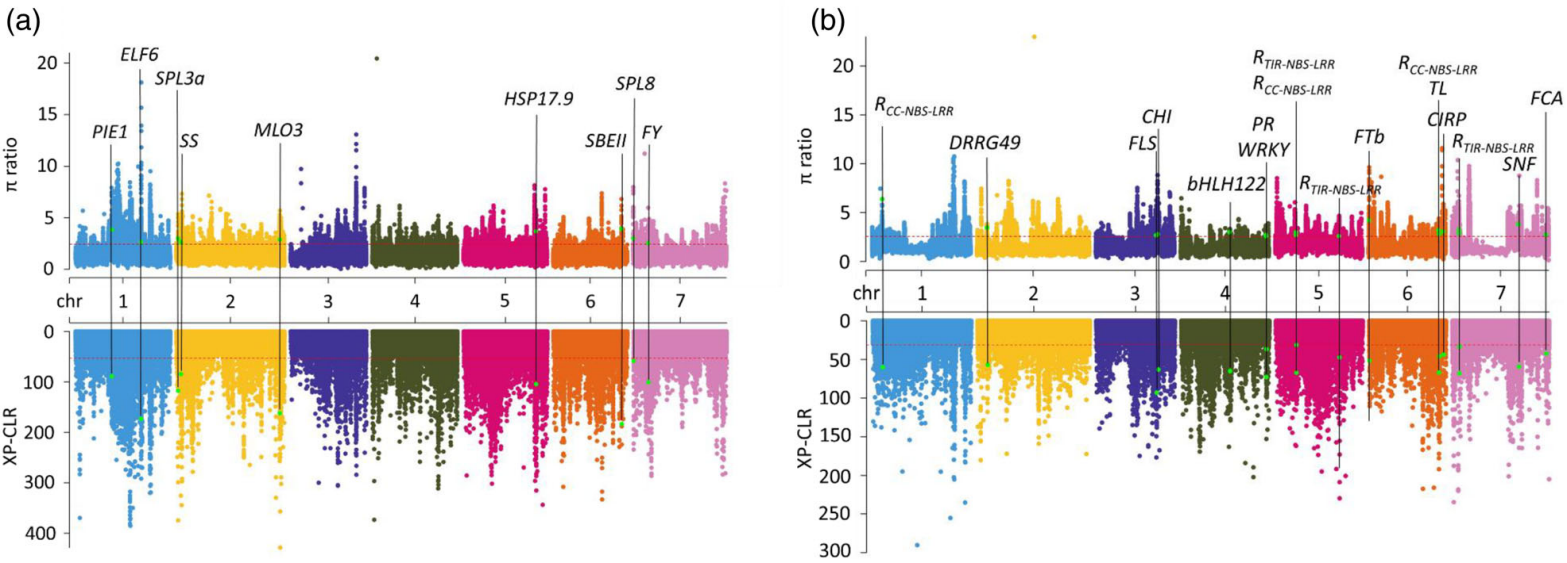
Genome‐wide distribution of selective sweeps in lentil. (a) Selective sweeps in *L. culinaris* landraces compared with *L. orientalis*. (b) Selective sweeps in *L. culinaris* cultivated lines compared with *L. culinaris* landraces. Selective sweep regions inferred from π ratio statistics (top) and XP‐CLR (bottom). The horizontal dashed lines indicate the cutoffs (top 5%). Possible candidate genes under selection are labeled in green.

Environment and human selection played an important role in shaping the lentil genomes during domestication. To investigate the selection signals in *Lens* species, we used XP‐CLR and *π* ratio analysis to identify candidate regions that scored in the top 5% of both methods. *L. culinaris* landraces were first compared with *L. orientalis* to identify the selection signals associated with changes in traits. In total, 1095 selective sweeps covering 193.3 Mb and containing 3334 candidate genes were identified (Table S11). We found genes encoding starch branching protein II (SBEII) and starch synthase (SS) within the selective sweeps, indicating potential alteration of starch accumulation (Hennen‐Bierwagen et al., 2008) (Figure 5a). Notably, flowering time regulation genes *ELF6* (Yang et al., 2016), *PIE1* (Noh & Amasino, 2003), *FY* (Feng & Michaels, 2011), and *SPL3a* (Yuan et al., 2021), stress‐related genes *SPL8* (Gou et al., 2018) and *HSP17.9* (DeRocher et al., 1991), and disease‐related gene *MLO3* (Kusch & Panstruga, 2017) were detected in the regions under selection (Figure 5a and Table S11). To identify selective sweeps associated with improvement‐related traits, we compared *L. culinaris* cultivated accessions with the *L. culinaris* landrace group. About 948 selective sweeps covering 160.4 Mb with 2846 candidate genes were detected (Table S12). *FLS* and *CHI* have been previously implicated in alerting flavonoid content and flavonoid as a secondary metabolite has been shown to be connected with plant defense (Dai et al., 1996; Lu et al., 2017; Skadhauge et al., 2004). Moreover, disease resistance R genes encoding TIR‐NBS‐LRR and CC‐NBS‐LRR (McHale et al., 2006) were detected in the regions (Figure 5b). These findings indicated there was a strong selection against biotic stress during lentil breeding. We also found *ERD* (John et al., 2016) gene related to cold acclimation and *VIP3* and *FLC* genes critical in the vernalization pathway when comparing between Spring Small and Winter Small lentil types (Table S13). The protein‐coding genes within the sweeps showed enrichment for gene ontology (GO) terms including cysteine synthase activity (GO:0004124), calcium‐mediated signaling (GO:0019722), kinase binding (GO:0019900), mechanosensitive ion channel activity (GO:0008381), cysteine‐type endopeptidase inhibitor activity (GO:0004869), oxygen binding (GO:0019825), manganese ion transmembrane transporter activity (GO:0005384), cellular manganese ion homeostasis (GO:0030026), defense response to bacterium (GO:0042742) when comparing between Spring Large/Medium and Spring Small (Tables S14 and S18). However, there are no significant GO results for the other three comparisons. (Tables S15–S17).

### Identification of loci related to *Fusarium* root rot resistance

Six traits were measured for FRR resistance under two treatments (2I: 2 × 10^6^ spores/ml and 4I: 4 × 10^6^ spores/ml) using *L. culinaris* landrace and cultivated accessions (Figures S9 and S10, Tables S1 and S19). Three traits including shoot length, shoot dry weight, and root dry weight were also evaluated (Figure S11, Tables S1 and S19). We performed GWAS on the six individual traits and their comprehensive values using 22 532 545 SNPs and identified 419 associated peaks for 2I treatment and 212 associated peaks for 4I treatment (Figures S12–S14, Table S20), as well as 10 signals associated with root dry weight (Figure S15 and Table S20). Surprisingly, only one association signal was detected in both treatments (Table S21 and Figure S16). In addition, 73 associated signals were detected for at least two FRR traits under the 2I treatment (Figure S17a and Table S22), while 10 peaks associated with at least two FRR traits were found under the 4I treatment (Figure S17b and Table S23). FRR resistance levels in landraces were significantly higher than cultivated accessions (Figures S18 and S19). Furthermore, five identified signals associated with root dry weight overlapped with those associated FRR. Shoot length and dry weight of landraces were significantly higher than cultivated accessions (Figure S20).

**Figure 6:**
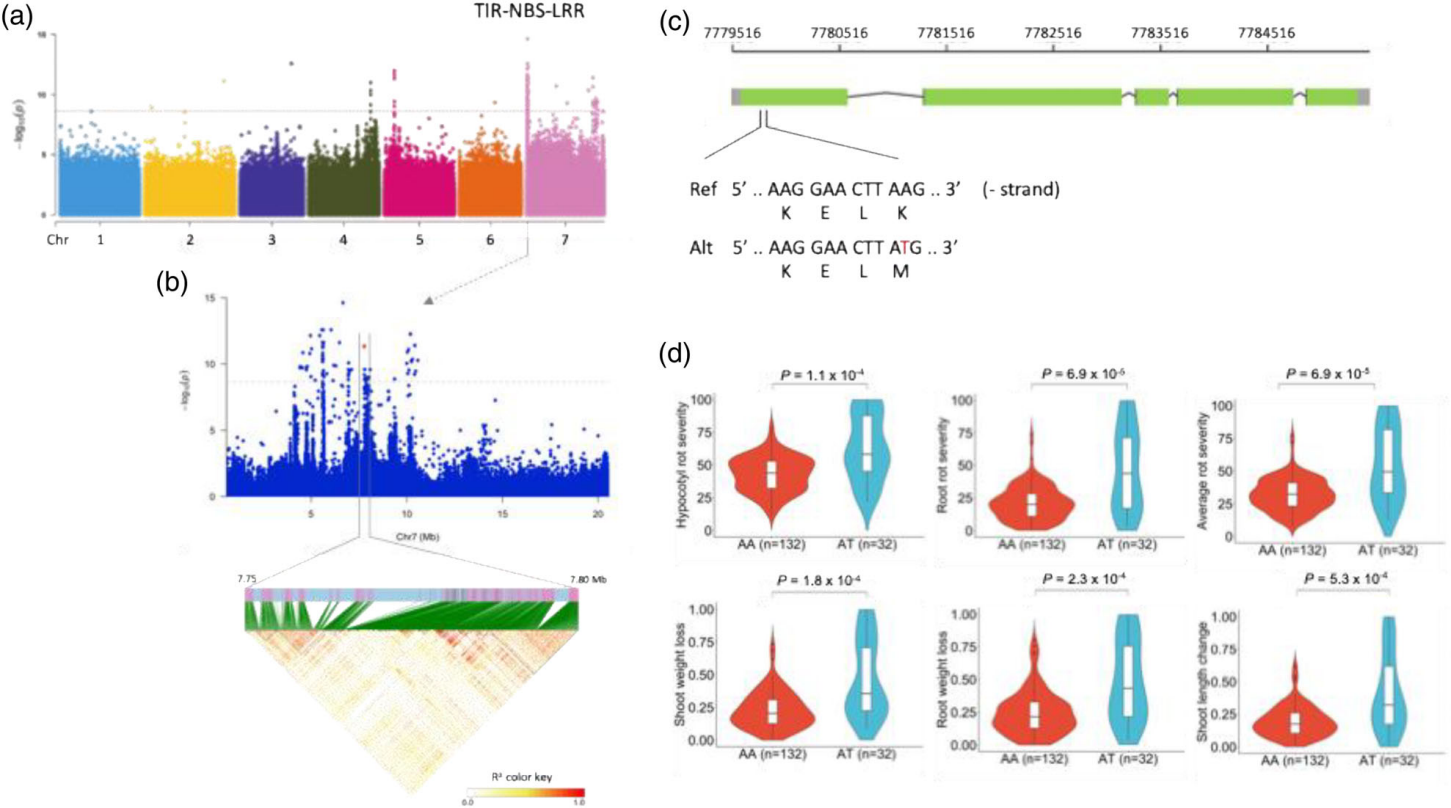
The genetic architecture underlying *Fusarium* root rot (FRR) resistance. (a) GWAS results from the analysis of shoot length change measured under the 2I treatment. The red horizontal dashed line represents the significance threshold (Bonferroni correction, *α* = 0.05). (b) Local Manhattan plot surrounding the peak for shoot length change on chromosome 7. The significantly associated SNP (chr7:7779810) within the gene *Lcu.2RBY.7g004590* encoding a toll‐interleukin receptor nucleotide‐binding site leucine‐rich repeat (TIR‐NBS‐LRR) protein is indicated by the red dot. The lower panel shows the LD blocks of significantly associated loci in the region spanning 7.75–7.80 Mb. (c) Gene structure of *Lcu.2RBY.7g004590* (*TIR‐NBS‐LRR*) and the SNP (A → T at chr7:7779810) introduced an amino acid change (Lys → Met) in the fifth exon of the gene. (d) Significant divergence of the lentil accessions carrying the AA alleles compared with the accessions carrying the AT alleles for the six FRR traits (hypocotyl rot severity, root rot severity, average rot severity, shoot weight loss, root weight loss, and shoot length change). The number (*n*) of accessions harboring the given alleles is shown in the parentheses. The significance was tested with Tukey's tests. In the violin plots, central line: median values; bounds of box: 25th and 75th percentiles; whiskers: 1.5*IQR (IQR: the interquartile range between the 25th and 75th percentile).

A significant GWAS signal on chromosome 7 was identified for three FRR traits, shoot dry weight loss, shoot length change, and comprehensive value (Figure 6a,b, Figure S21, and Table S22). The significantly associated SNP (chr7:7779810) is in the coding region of a candidate gene *Lcu.2RBY.7g004590*, encoding a toll‐interleukin receptor nucleotide‐binding site leucine‐rich repeat (TIR‐NBS‐LRR) protein, leading to a non‐synonymous amino acid change (K–M) (Figure 6c). The *TIR‐NBS‐LRR* gene is known as one of the most important resistance gene families in plants and is functionally responsible for plant resistance to pathogens (McDowell & Woffenden, 2003). It was worth noting that there were no lentil accessions carrying alternative homozygous alleles and a majority of lentil accessions (*n* = 132) harbored the reference alleles. Accessions carrying reference alleles had a significantly higher FRR resistance than accessions harboring heterozygous alleles (Figure 6d). Thus, the reference allele could have been preferably selected during lentil domestication.

We also detected a peak SNP in *ABP19a* (*Lcu.2RBY.7g005600*) at Chr7:10576840, encoding an auxin‐binding protein. *ABP19a* is an ortholog of the rice *OsGLP* that has been previously shown to be involved in general plant defense responses (Lane, 2002; Manosalva et al., 2009). This significant SNP was identified in four FRR traits: root rot severity, average rot severity, shoot length change, and comprehensive value (Figure S22 and Table S22). Haplotype analysis revealed the candidate gene *Lcu.2RBY.7g005600* had eight major haplotypes and significant differences were detected among them (Figures S23a and S23b). The favorable haplotype is dominant in accessions originating from South Asia, while a high frequency of the unfavorable haplotype was observed in the Spring Large/Medium group (Figure S23c). Additionally, this gene was located within a selective sweep (*L. culinaris* landrace vs *L. culinaris* cultivated) (Table S22), indicating it underwent selection for improved FRR resistance during lentil domestication and breeding.

### Identification of loci related to agronomic traits

Thirteen quantitative traits were evaluated on *L. culinaris* accessions (Table S24 and Figure S24). Ten traits were evaluated across 3 years while three traits, including pod shatter, pod drop, and seed weight, were assessed only in 2022. Drought and abnormally high temperatures in 2021 (Figure S25) affected plant growth, resulting in low yield (Figure S24). A total of 700, 530, and 1018 associated peaks were identified for agronomic traits in 2020, 2021, and 2022, respectively. Among them, 175, 119, and 261 associated variants were located in gene regions (exonic or intronic sequences) or very close by up/downstream (within 5 kb of the genes). No association signals for the same traits were shared across 3 years, whereas traits with higher heritability, such as days to flowering and days to swollen pods, were those with the highest number of shared association signals (Tables S25–S27).

## DISCUSSION

Resequencing has been performed on various legumes including soybean (Kim et al., 2021; Zhou et al., 2015), pigeon pea (Varshney et al., 2017), chickpea (Varshney et al., 2019), common bean (Wu et al., 2020), rice bean (Guan et al., 2022), and mung bean (Liu et al., 2022); however, it has not yet been applied to lentil. Lentil is an important grain legume to promote both a sustainable and resilient agroecosystem. In the present work, we analyzed genome sequences of 238 *Lens* accessions, which encompassed landraces, cultivated lines, and wild species, and provided more than 112 million sequence variants. Higher variation was observed in wild species, as compared with landraces and cultivated lines. Among wild species, *L. orientalis* and *L. ervoides* had more unique variants than others.

Understanding population structure and phylogenetic relationship holds significant importance in genebank management and germplasm utilization. Our analyses revealed taxonomic status and gene pool classification of *L. culinaris* and related wild species. *L. nigricans* is the most distantly related wild species to domesticated *L. culinaris*, consistent with most conclusions from previous work (Liber et al., 2021; Ogutcen et al., 2018; Wong et al., 2015). Our study indicated *L. ervoides* is a sister of *L. odemensis*, *L. orientalis*, and *L. culinaris*, corroborating results identified from Wong et al.^15^ as opposed to Ogutcen et al.^70^ and Liber et al.^16^ *L. orientalis* is the closest species to *L. culinaris*, confirming it is the wild progenitor of the cultivated species. Population structure and phylogenetic analysis identified taxonomic discrepancies for three accessions of wild species which can now be rectified. Through the evaluation of taxonomic relationships, our study demonstrated the value of WGS in germplasm management and crop improvement.

The Near East represents a major domestication center for many important crops, including wheat, barley, and chickpea (Lev‐Yadun et al., 2000; Vavilov, 1992). By analyzing nucleotide diversity, fixation index, and gene flow, we confirmed the Near East region is the center of origin of lentil as well. Domestication and cultivation of lentil is closely associated with wheat and barley (Zohary & Hopf, 1973). Archeological evidence dates the existence of lentil back to 11 000 BC (~13 Kya) in Greece and 8500 BC (~10 Kya)–7500 BC (~9.5 kya) in Syria. However, there is no conclusive evidence provided by archeological remains about the site of lentil domestication (Sonnante et al., 2009). In our study, by accessing the change of effective population size of *L. orientalis* and *L. culinaris*, we found the domestication time of cultivated lentil was estimated to be around 12 Kya during the Pre‐Pottery Neolithic A period. Another interesting result is *L. culinaris* landraces did not cluster based on their geographic distribution, except for those from Near East and South Asia. It is worth noting that *L. culinaris* landraces from South Asia are distinct and presented low diversity, similar to previous findings (Guerra‐Garcia et al., 2022; Ma et al., 2020). Both environmental and human‐mediated factors may contribute to the decline of genetic variation in this region.

Currently, Canada produces the majority of the world's lentils. As the geographic dispersion of lentil production widens, so too must the research on breeding for adaptability in new agroecosystems increase. We conducted a comprehensive GWAS analysis for 13 agronomic traits across multiple years, as well as resistance to FRR under controlled environments to identify causal loci controlling important traits. FRR is a major threat to lentil production. A previous study revealed 11 QTL through GWAS using a SNP data set derived from genotyping by sequencing. We deployed approximately 4000 times more molecular markers than the previous GWAS study, reporting here 631 marker trait associations. Among them, 237 overlapped with selective sweeps for domestication and improvement of lentil, indicating the resistance trait was intentionally selected for during the domestication process. We found two candidate genes (*TIR‐NBS‐LRR* and *ABP19a*) associated with FRR resistance in lentil. Among them, *TIR‐NBS‐LRR* has been characterized as a large class of functionally defined resistance genes (R‐genes) that play critical roles in defense against pathogen attack in many crops, including Arabidopsis (Meyers et al., 2003), soybean (Zhou et al., 2022), cotton (Li et al., 2019), and pepper (Wan et al., 2012). However, due to the current limitation of the genetic transformation system in lentil, functional analysis of these candidate genes is challenging. Future studies should be taken to validate the roles of these genes and to assess the robustness of the QTL identified for agronomic traits across diverse environments.

Collectively, our study provides a valuable resource for understanding population genetics, center of origin, domestication, as well as the genetic basis underlying important complex traits. The genetic and genomic resources will facilitate future breeding for lentil crop improvement.

## MATERIALS AND METHODS

### DNA sample preparation and sequencing

The diversity panel used in this study included 238 lentil accessions, comprised of 112 cultivated lines, 71 landraces, and 55 wild *Lens* accessions which consisted of 18 *Lens orientalis*, 4 *Lens odemensis*, 28 *Lens ervoides*, and 5 *Lens nigricans* (Table S1 and Figure S26). We also included the reference genotype (CDC Redberry) for whole‐genome resequencing to validate variant calling accuracy. Landraces and wild *Lens* accessions were obtained from the Western Regional Plant Introduction Station, USDA‐ARS, Pullman, WA, USA, whereas cultivated lines were from the USDA‐ARS Grain Legume Genetics and Physiology Research Unit, Pullman, WA. Total DNA was extracted from each lentil accession using the DNeasy 96 Plant Kit (QIAGEN, Valencia, CA, USA) from approximately 0.1 g young leaf tissue. For each accession, 250 ng of genomic DNA was used to construct a sequencing library following the manufacturer's instruction of the KAPA Hyper Prep kit (Roche Applied Science, Pleasanton, CA, USA), with a mean insert size of 425 bp. 150 bp paired‐end reads were generated on an Illumina Novoseq sequencer at QB3 Genomics Lab at the University of California, Berkeley. Clean reads were obtained using Fastp (Chen et al., 2018) by removing reads containing adapter or >5% “N,” short reads (read length <80 bp), low‐quality reads (with the parameter ‐q 15, ‐u 40, ‐5, ‐‐cut_front_window_size 1, ‐‐cut_front_mean_quality 10, ‐r, ‐‐cut_right_window_size 4, ‐‐cut_right_mean_quality 15). 3 bp off the 5′ end and 1 bp off the 3′ end of a read were also trimmed.

### Variation calling and annotation

Paired‐end resequencing reads were mapped to the lentil reference genome version 2.0 (Ramsay et al., 2021) using bwa‐mem2 (version 2.1) (Vasimuddin et al., 2019) with default parameters. SAMtools (version 1.10) (Li et al., 2009) was used to convert mapping results to BAM format and sort BAM files. Duplicate reads were marked with Picard (version 2.21.9). Depth and genome coverage of mapped reads were estimated using BEDTools (version 2.29.2) (Quinlan & Hall, 2010). Variant detection followed the Best Practices Workflows recommended by GATK toolkit (McKenna et al., 2010). HaplotypeCaller was used to generate GVCF files. Per‐sample GVCF files were consolidated by GenomicsDBImport, and genetic variants were jointed using GenotypeGVCFs in GATK toolkit (version 4.1.4.1) (McKenna et al., 2010). With the SelectVariants and the VariantFiltration functions in GATK, SNPs, and indels were selected, and hard filtering was applied using parameters “QD <2.0 || SOR >3.0 || FS >60.0 || MQ <40.0 || MQRankSum <−12.5 || ReadPosRankSum <−8.0” to raw SNPs, and “QD <2.0 || FS >200.0 || ReadPosRankSum <−20.0” to raw indels. BCFtools (version 1.10.2) (Li, 2011) was further applied to filter SNPs and indels using following criteria: (1) bi‐allelic; (2) <30% missing call rate; (3) minor allele frequency >0.008; (4) loci present in chromosomes. In addition, only insertions and deletions less than or equal than 5 bp were retained for indels. Filtered variants were counted using BCFtools (version 1.10.2) (Li, 2011) on different chromosomes, lentil lines, and groups. Venn diagrams were displayed by the Venn function of the Venn package in the R 4.0.3 program (Team, R, 2013). ANNOVAR (Wang et al., 2010) were used to annotate filtered variants based on gene annotations of lentil reference genome version 2.0 (Ramsay et al., 2021). SNP and indel density were estimated using VCFtools (version 0.1.16) (Danecek et al., 2011).

Copy number variations (CNVs) were identified using CNVcaller (Wang, Zheng, et al., 2017). Briefly, aligned reads were counted across the reference genome segmented using 800 bp sliding windows, and the comparable read depth of each lentil line was generated. CNV regions were detected with parameters “‐f 0.1 ‐h 2 ‐r 0.15”. CNV with minor allele frequency >0.008 and SILHOUETTESCORE >0.5 were retained.

### Population genetic analysis

We used 379 235 SNPs at fourfold‐degenerate sites for phylogenetic and population structure analysis. A neighbor‐joining phylogenetic tree was conducted using MEGA X (Kumar, Stecher, et al., 2018) with 500 bootstraps, employing the p‐distance substitution model, and *L. nigricans* was used as an outgroup. The iTOL version 5 (https://itol.embl.de/) was used to display the phylogenetic tree. Population structure analysis was performed using ADMIXTURE (version 1.3.0) (Alexander et al., 2009) with the number of clusters K ranging from 2 to 20 and a default cross‐validation setting (‐‐cv = 5). The optimal K was estimated by cross‐validation errors calculated from 20 independent runs for each K. PCA was performed using PLINK (version 1.90) (Purcell et al., 2007) with the entire SNP dataset (103 290 296 SNPs), and the first three eigenvectors were plotted.

The fixation index (*F*_ST_) and nucleotide diversity (π) were estimated based on the entire SNP dataset using VCFtools (version 0.1.16) (Danecek et al., 2011). A 100 kb sliding window with step size of 10 kb was applied. To estimate LD decay, we calculated the correlation coefficient (*r* ^*2*^) for all pairs of SNPs within 500 kb using PopLDdecay (version 3.41) (Zhang et al., 2019) with parameters “‐MAF 0.05 ‐Miss 0.1 ‐Het 0.2.”

### Gene flow analysis

TreeMix (version 1.13) (Pickrell & Pritchard, 2012) was used to estimate gene flow between groups using LD‐pruned SNPs, which were generated by pruning the entire SNP dataset with the pairwise correlation >0.2 in a sliding window of 50 SNPs in steps of five SNPs using PLINK software (version 1.9) (Chang et al., 2015). The parameters used in TreeMix were “‐se ‐bootstrap ‐k 1000 ‐m,” wherein the migration event ‐m ranged from 1 to 8. In addition, gene flow between groups was detected using the ABBA‐BABA test (Martin et al., 2015) with all the SNPs. The *D* values were weighted using the genotype frequency of the outgroup. Significance of the *D* values was estimated based on *Z* scores obtained using the jackknife method (Efron & Stein, 1981).

### Demographic history inference

MSMC2 (version 2.1.3, https://github.com/stschiff/msmc2) was employed to estimate effective population size and infer the demographic history. The input files for MSMC2 were generated by MSMC Tools (https://github.com/stschiff/msmc‐tools). Briefly, only sites with mean coverage depth >10, and mapping quality and base quality >20 were used in the analysis. Masking files were generated using the script bamCaller.py and SNPs were phased using BEAGLE (version 5.2) (Browning et al., 2018). Reference genome mask files were generated using the SNPable tool (http://lh3lh3.users.sourceforge.net/snpable.shtml) and tools available at https://github.com/lh3/misc. First, the reference genome was split into overlapping 35‐mers subsequences, and these subsequences were then aligned to the reference genome using BWA with parameters “aln ‐R 1000000 ‐O 3 ‐E 3.” Final reference genome mask files were generated using the script gen_mask (‐l 35 ‐r 0.5). Scaled times and population sizes were converted into years and sizes by assuming a generation time of 1 year and a mutation rate of 8.3 × 10^−9^, as used previously (Ramsay et al., 2021). The estimation was performed on randomly selected three samples from each group and repeated four times.

SMC++ (version 1.15.2) (Terhorst et al., 2017) was used to infer population effective size and split time among different lentil groups. In brief, input files were generated using vcf2smc. The population effective size was estimated using SMC++ estimate and split time was inferred using SMC++ split. A generation of 1 year and a mutation rate of 8.3 × 10^−9^ was used. The estimation was performed on randomly selected 10 samples from each group and repeated three times.

### Selective sweep analysis

We used two approaches to detect candidate regions and genes potentially affected by selections. *π* ratio was estimated using VCFtools (version 0.1.16) (Danecek et al., 2011) on a 100 kb sliding window with step size of 10 kb. Windows containing less than 10 SNPs were not included. We also performed a cross‐population composite likelihood ratio test (XP‐CLR, version 1.1.2) (Chen et al., 2010) to investigate the selection signals. XP‐CLR scores were calculated with the parameters “‐‐ld 0.95 ‐‐maxsnps 100 ‐‐size 100000 ‐‐step 10000” for each chromosome. Regions in the top 5% of values/scores from both approaches were considered as putative selective sweeps. Genes within selective regions were used to perform GO enrichment analysis in R package ClusterProfiler (version 3.18.1) (Yu et al., 2012). Selective sweeps were identified in *L. orientalis* versus *L. culinaris* landrace and *L. culinaris* landrace versus *L. culinaris* cultivated, Spring Large/Medium versus Spring Small, Spring Small versus Winter Small.

### Field experiment and phenotyping

All *L. culinaris* accessions were planted in a field at the USDA‐ARS Central Ferry Research Farm (latitude, 46°39′5.1″ N; longitude, 117°45′45.4″ W) in April of 2020–2022, using a randomized complete block design with four replicates. The field was treated with a pre‐emergence herbicide (S‐metolachlor; Dual II Magnum, Syngenta) and seeds were treated with a fungicide mix (mefenoxam [13.3 ml a.i. 45 kg − 1], fludioxonil [2.4 ml a.i. 45 kg − 1], and thiabendazole [82.9 ml a.i. 45 kg − 1]) prior to planting. About 30 seeds of each accession were sown in 1.5 m single row plots with 1.5 m spacing between rows and irrigated with a subsurface drip irrigation system. Phenotypic data were collected on 10 agronomic traits in 2020–2022, including plant stand, days to flowering, days to swollen pods, days to maturity, canopy height, plant height, lodging, height to lowest pod, biomass and seed yield, while three traits, including pod shatter, pod drop, and seed weight, were only assessed in 2022. Plant stand was recorded 7 weeks after sowing. Days to flowering, days to swollen pods, and days to maturity were recorded as the number of days from sowing to when 10% of plants in a plot had at least one fully open flower, 10% of plants had at least one pod with fully swollen seeds, and 90% of plants displayed maturity, respectively. Canopy height and plant height were measured as whole plots at harvest. Lodging was estimated as follows: lodging (%) = [1 − (canopy height/plant height)] × 100. Height to lowest pod was collected by measuring the distance from the ground to the first branch with first pod. Pod shatter and pod drop were determined using a 1 (none) to 5 (all) scoring scale and recorded at harvest. Biomass and seed yield in each plot were collected after plants were harvested and thoroughly dried.

### *Fusarium* root rot disease phenotyping

The *L. culinaris* landrace and cultivated accessions were evaluated for the reaction to *Fusarium avenaceum* under greenhouse conditions (25°C 16 h/23°C 8 h) following the procedure described in Heineck et al. (2022). Briefly, three isolates collected from three production fields in Montana, USA were equally mixed and used for inoculation. Prior to inoculation, seeds were surface sterilized in a 1% detergent solution (Alconox, White Plains, New York, USA) for 30 sec followed by a 0.5% solution of NaClO (KIK International LLC, Concord, Ontario, CA) for 10 min, rinsed in distilled water three times and then air‐dried for 48 h. Sterilized seeds were planted into 21 cm deep Cone‐tainers (Stuewe and Sons, Tangent, OR, USA) filled with perlite (Supreme Perlite, Portland, OR) using a randomized split‐plot design (inoculation treatment as main plots and accessions as the split‐plot). Eight blocks for each treatment were planted over 8 days and harvested 28 days after sowing. During sowing, seeds of inoculated sets were pipetted with 2 ml of inoculum at 2 × 10^6^ spores/ml or 4 × 10^6^ spores/ml before covering with perlite, while non‐inoculated sets were treated with 2 ml of distilled water. At harvest, root rot severity and hypocotyl rot severity were determined by accessing roots and hypocotyls using a 0–100% scale. Average root rot was calculated by averaging root rot severity and hypocotyl rot severity. Shoot length was measured from each plant. Roots and shoots were separated and placed in a forced‐air dryer at 60°C for 5 days. Shoot and root dry weights were subsequently collected from each plant. Shoot/root dry weight loss and shoot length change were calculated with (dry weight or shoot length per plant of non‐inoculated treatment—dry weight or shoot length per plant of inoculated treatment)/dry weight or shoot length per plant of non‐inoculated treatment.

### GWAS analysis

A large‐scale GWAS was carried out for 19 traits (six FRR traits and 13 agronomic traits) using 22 532 545 SNPs (MAF ≥0.05 and miss ≤0.1), which were imputed using BEAGLE (version 5.2). To integrate multiple FRR‐related traits into a single quantitative index, each trait was standardized using the affiliation function method and weighted by its standard deviation. A comprehensive value for each accession was calculated as the weighted sum of standardized traits, and GWAS was also performed using the comprehensive values. We used the genome‐wide efficient mixed‐model association (GEMMA) (version 0.98.5) package with a univariate linear mixed model. Bonferroni‐corrected significance thresholds were estimated as 0.05/*n* (where *n* is the effective number of SNPs). Haplotype blocks were estimated using LDBlockShow (version 1.4) (Dong et al., 2021).

## AUTHOR CONTRIBUTIONS

YM performed most of the experiments, including collecting and analyzing data and writing manuscript. DM provided high‐performance computing resources. MB acquired the funding to support this study. LDP and GCH conducted *Fusarium* root rot experiment. YM, CJC, RU, MLW, and BB carried out field experiment. HS provided supports on validating genome‐wide association signals. KB contributed the reference genome for data analysis. CJC and RJM provided the materials, conceived the plan, and supervised the project. All authors contributed to the final manuscript.

## CONFLICT OF INTEREST

The authors declare no conflicts of interest.

## Supporting information


**Figure S1.** Violin plots of the coverage of each group across each chromosome of the lentil genome.
**Figure S2.** Venn diagram of unique and shared SNPs among different lentil groups.
**Figure S3.** Venn diagram of unique and shared indels among different lentil groups.
**Figure S4.** A phylogenetic tree inferred the evolutionary relationship of five lentil species.
**Figure S5.** Cross‐validation errors for K values.
**Figure S6.** Principle component analysis (PCA) plots for lentil accessions.
**Figure S7.** Linkage disequilibrium (LD) decay among different lentil groups.
**Figure S8.** Demographic history of *L. culinaris* (landrace) and *L. culinaris* (cultivated).
**Figure S9.** Frequency distribution of phenotypic variation of six traits for *Fusarium* root rot (4 × 10^6^ spores/ml) in 183 *L. culinaris* landrace and cultivated accessions.
**Figure S10.** Frequency distribution of phenotypic variation of six traits for *Fusarium* root rot (2 × 10^6^ spores/ml) in 183 *L. culinaris* landrace and cultivated accessions.
**Figure S11.** Frequency distribution of phenotypic variation of shoot length, shoot/root dry weight in 183 *L. culinaris* landrace and cultivated accessions.
**Figure S12.** The distribution of associated loci identified for *Fusarium* root rot (FRR).
**Figure S13.** Genome‐wide association study (GWAS) for *Fusarium* root rot (FRR) resistance under the 2I treatment.
**Figure S14.** Genome‐wide association study (GWAS) for *Fusarium* root rot (FRR) resistance under the 4I treatment.
**Figure S15.** Genome‐wide association study (GWAS) for shoot length and dry matters.
**Figure S16.** Local Manhattan plots of genome‐wide association on chromosome 7 (Lcu.2RBY.Chr7: 467130974) discovered under both treatments (2I and 4I) for *Fusarium* root rot (FRR).
**Figure S17.** UpSet plots of Genome‐wide association study (GWAS) loci for *Fusarium* root rot (FRR) resistance.
**Figure S18.** Phenotypic differences of *Fusarium* root rot traits (2I) between *L. culinaris* landrace and cultivated accessions.
**Figure S19.** Phenotypic differences of *Fusarium* root rot traits (4I) between *L. culinaris* landrace and cultivated accessions.
**Figure S20.** Phenotypic differences of shoot length and root and shoot dry weight between *L. culinaris* landrace and cultivated accessions.
**Figure S21.** GWAS result from the analysis of shoot weight loss and comprehensive value measured under the 2I treatment.
**Figure S22.** GWAS result from the analysis of root rot severity, average rot severity, shoot length change and comprehensive value measured under the 2I treatment.
**Figure S23.** Haplotype analysis on the candidate gene *ABP19a* (*Lcu.2RBY.7g005600*).
**Figure S24.** Frequency distribution of phenotypic variation of 13 agronomic traits collected over 3 years for 183 *L. culinaris* landrace and cultivated accessions.
**Figure S25.** Weather temperature comparison for April to August in 2020, 2021, and 2022.
**Figure S26.** Seed variation among different *Lens* groups.

**Table S1.** List of the whole‐genome resequenced 238 lentil accessions and their associated phenotypic data.
**Table S2.** Summary statistics of sequencing data for 238 lentil accessions.
**Table S3.** Summary of single‐nucleotide polymorphisms (SNPs) across each chromosome.
**Table S4.** Summary of indels across each chromosome.
**Table S5.** Summary of indels across each lentil group.
**Table S6.** Summary of single‐nucleotide polymorphisms (SNPs) across each lentil accession.
**Table S7.** Summary of Indels across each lentil accession.
**Table S8.** Taxonomic discrepancies and putative admixed accessions.
**Table S9.** The nucleotide diversity (π) of different lentil groups.
**Table S10.** The estimated fixation index (*F*_ST_) among different lentil groups.
**Table S11.** Genes within the selective sweep regions (*L. orientalis* versus *L. culinaris* landrace).
**Table S12.** Genes within the selective sweep regions (*L. culinaris* landrace versus *L. culinaris* cultivated).
**Table S13.** Genes within the selective sweep regions (Spring Small versus Winter Small).
**Table S14.** Genes within the selective sweep regions (Spring Large/Medium versus Spring Small).
**Table S15.** GO terms for the genes within the selective sweep regions (*L. orientalis* versus L. *culinaris* landrace).
**Table S16.** GO terms for the genes within the selective sweep regions (*L. culinaris* landrace versus *L. culinaris* cultivated).
**Table S17.** GO terms for the genes within the selective sweep regions (Spring Small versus Winter Small).
**Table S18.** GO terms for the genes within the selective sweep regions (Spring Large/Medium versus Spring Small).
**Table S19.** Statistical analysis results of *Fusarium* root rot traits for *L. culinaris* landrace and cultivated accessions.
**Table S20.** Genome‐wide association signals of *Fusarium* root rot traits in the *L. culinaris* accessions.
**Table S21.** Overlapped genome‐wide association signals of *Fusarium* root rot traits under both 2I and 4I treatments in the *L. culinaris* accessions.
**Table S22.** Common association signals detected for at least two *Fusarium* root rot (FRR) traits under the 2I treatment in the *L. culinaris* accessions.
**Table S23.** Common association signals detected for at least two *Fusarium* root rot (FRR) traits under the 4I treatment in the *L. culinaris* accessions.
**Table S24.** Statistical analysis results of agronomic traits for the *L. culinaris* accessions.
**Table S25.** Genome‐wide association signals related to agronomic traits collected in 2020 for the *L. culinaris* accessions.
**Table S26. G**enome‐wide association signals related to agronomic traits collected in 2021 for the *L. culinaris* accessions.
**Table S27.** Genome‐wide association signals related to agronomic traits collected in 2022 for the *L. culinaris* accessions.

## Data Availability

Whole‐genome resequencing raw data are available at the National Center for Biotechnology Information‐Sequence Read Archive (NCBI‐SRA) database (https://www.ncbi.nlm.nih.gov/sra) under accession number PRJNA1128027. The genotyping data are available from the Dryad repository: https://doi.org/10.5061/dryad.n02v6wxbh. All data supporting the findings of this study are available within the paper and within its Supporting Information.
