# Supplementary material for: Insights into lentil diversity, domestication, and the genetic basis of important agronomic traits through resequencing of 238 Lens accessions
Source: Plant J. 2026 May 14;126:e70908. doi: 10.1111/tpj.70908 (PMC13173320; doi:10.1111/tpj.70908)
Supplement: Supplementary file 1 — Figure S1. Violin plots of the coverage of each group across each chromosome of the lentil genome. Figure S2. Venn diagram of unique and shared SNPs among different lentil groups. Figure S3. Venn diagram of unique and shared indels among different lentil groups. Figure S4. A phylogenetic tree inferred the evolutionary relationship of five lentil species. Figure S5. Cross‐validation errors for K values. Figure S6. Principle component analysis (PCA) plots for lentil accessions. Figure S7. Linkage disequilibrium (LD) decay among different lentil groups. Figure S8. Demographic history of L. culinaris (landrace) and L. culinaris (cultivated). Figure S9. Frequency distribution of phenotypic variation of six traits for Fusarium root rot (4 × 10^6^ spores/ml) in 183 L. culinaris landrace and cultivated accessions. Figure S10. Frequency distribution of phenotypic variation of six traits for Fusarium root rot (2 × 10^6^ spores/ml) in 183 L. culinaris landrace and cultivated accessions. Figure S11. Frequency distribution of phenotypic variation of shoot length, shoot/root dry weight in 183 L. culinaris landrace and cultivated accessions. Figure S12. The distribution of associated loci identified for Fusarium root rot (FRR). Figure S13. Genome‐wide association study (GWAS) for Fusarium root rot (FRR) resistance under the 2I treatment. Figure S14. Genome‐wide association study (GWAS) for Fusarium root rot (FRR) resistance under the 4I treatment. Figure S15. Genome‐wide association study (GWAS) for shoot length and dry matters. Figure S16. Local Manhattan plots of genome‐wide association on chromosome 7 (Lcu.2RBY.Chr7: 467130974) discovered under both treatments (2I and 4I) for Fusarium root rot (FRR). Figure S17. UpSet plots of Genome‐wide association study (GWAS) loci for Fusarium root rot (FRR) resistance. Figure S18. Phenotypic differences of Fusarium root rot traits (2I) between L. culinaris landrace and cultivated accessions. Figure S19. Phenotypic differences of Fusarium root [file TPJ-126-0-s001.docx]

**
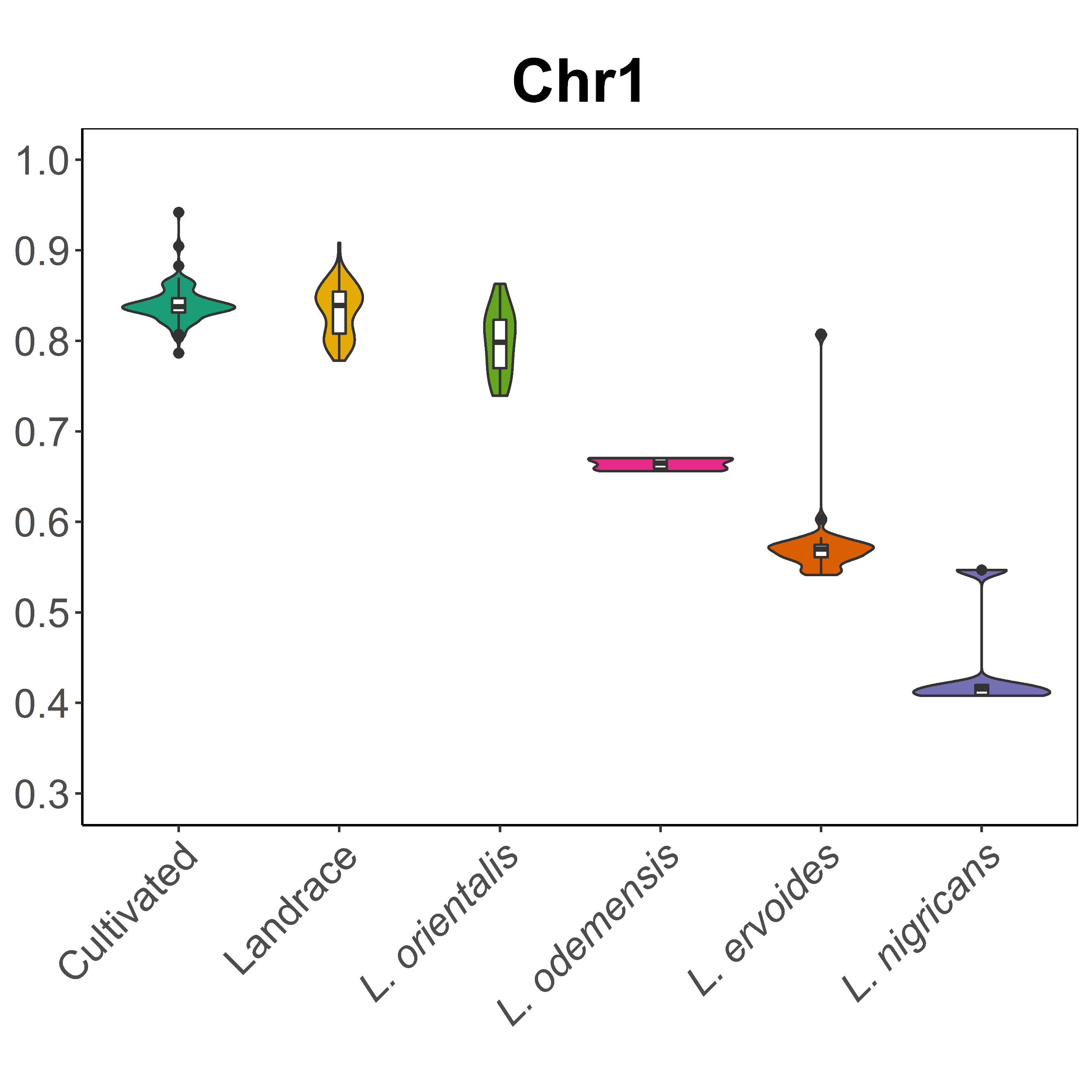
**
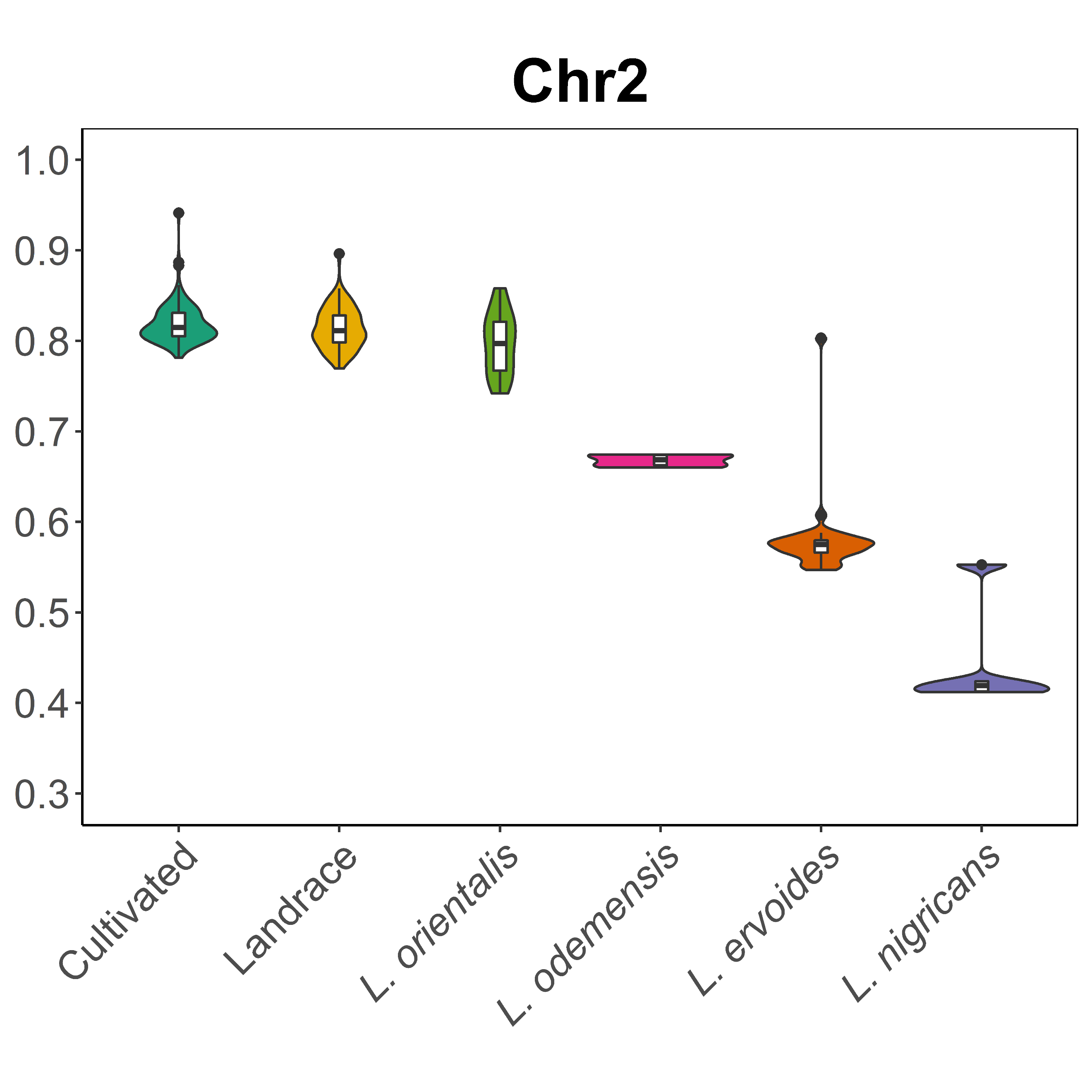

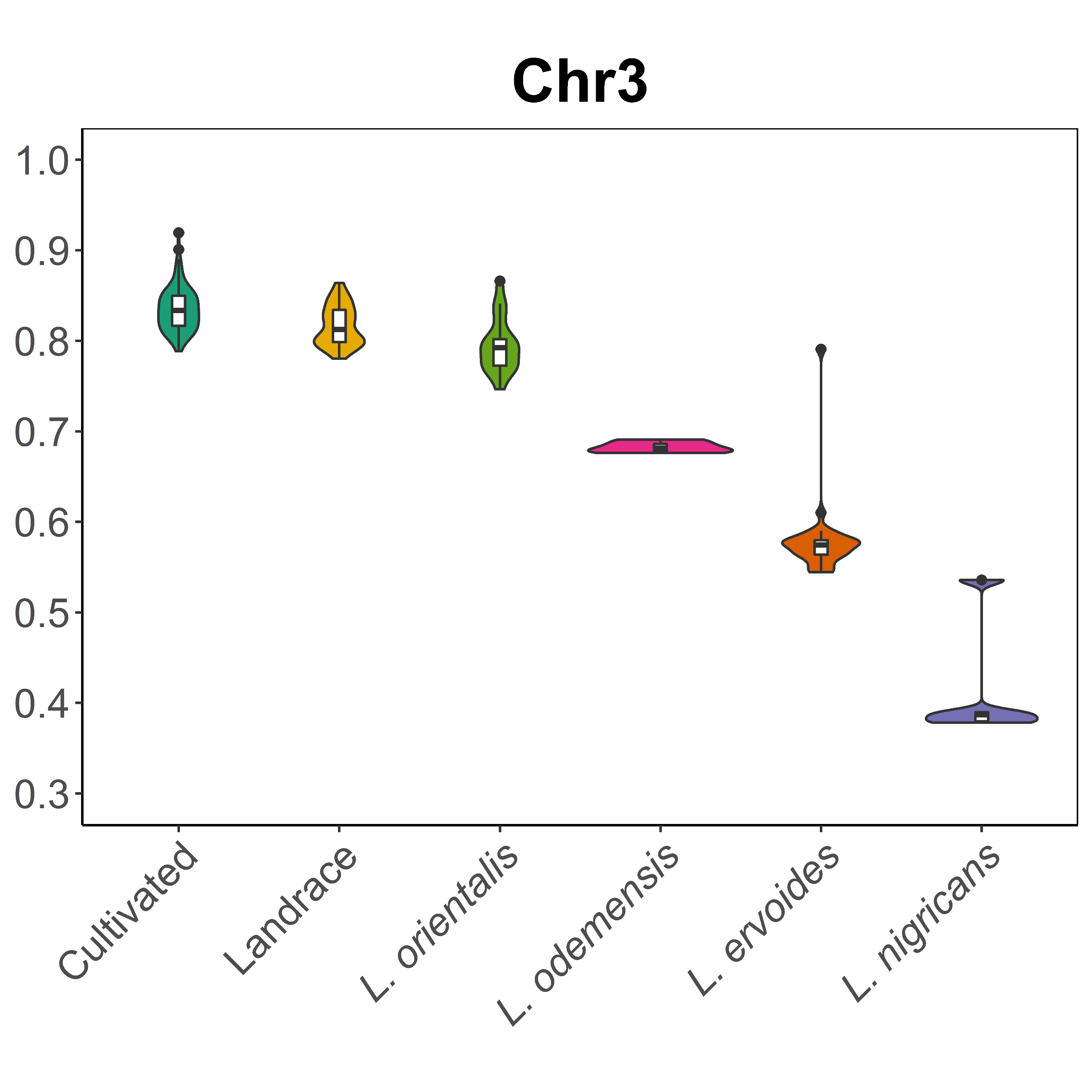

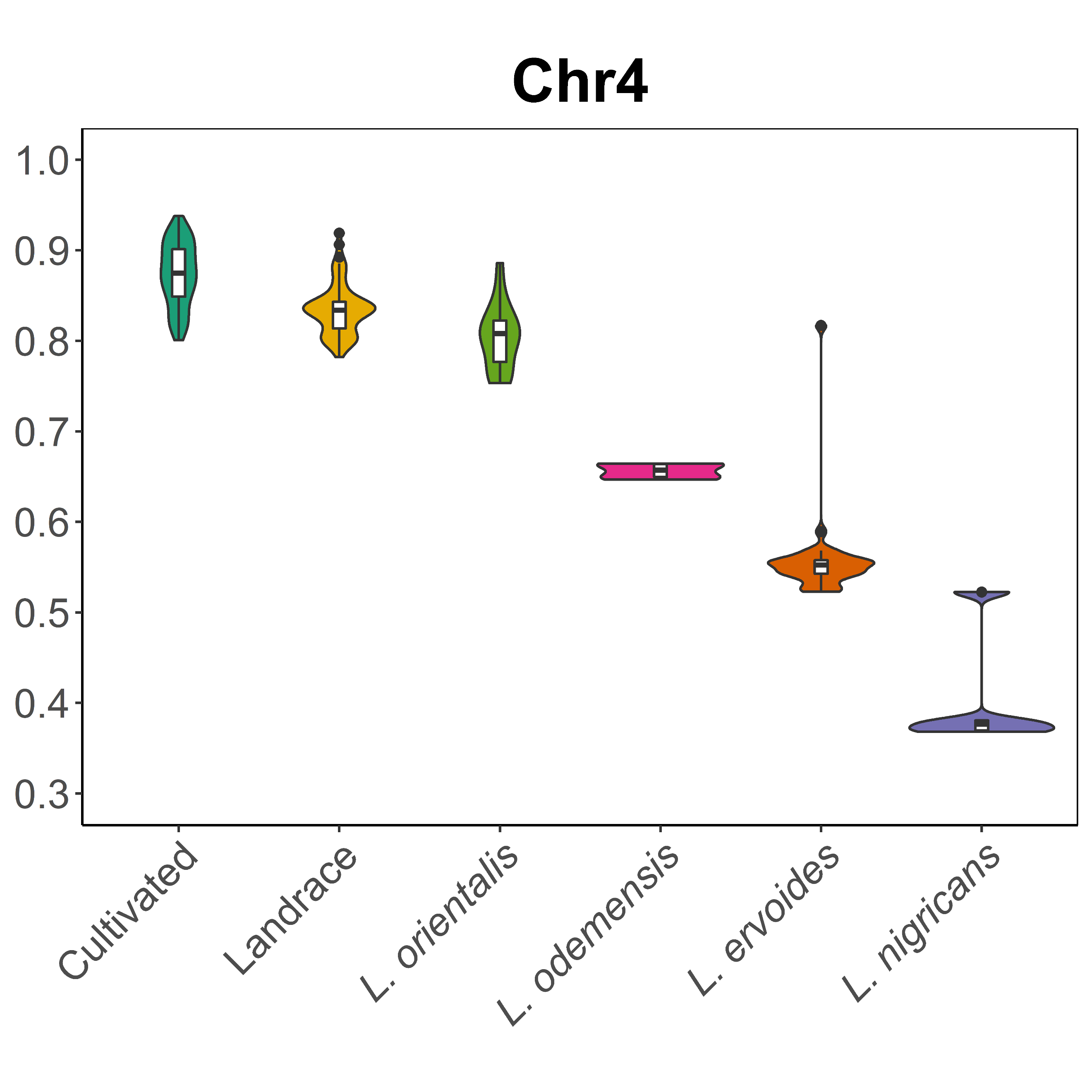

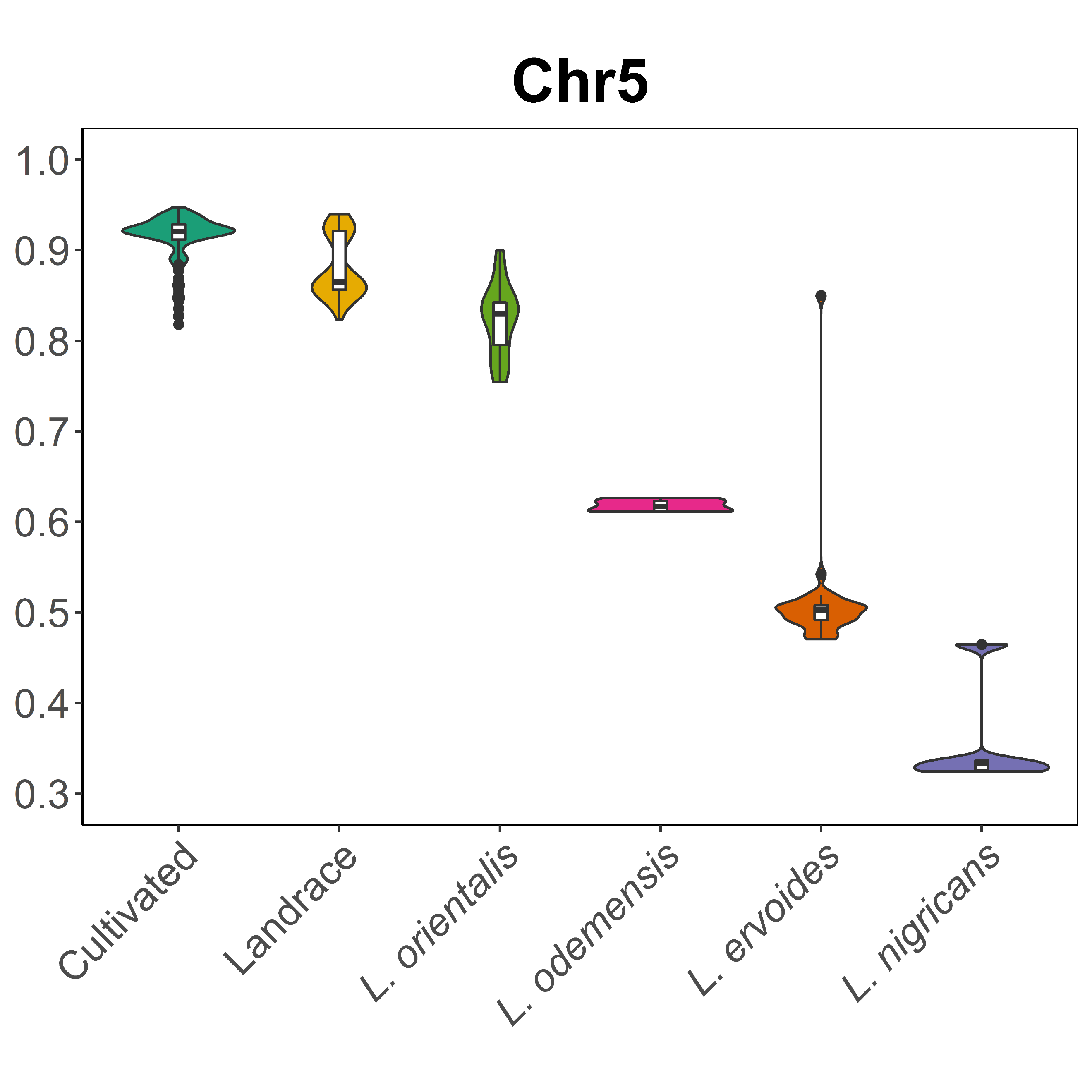

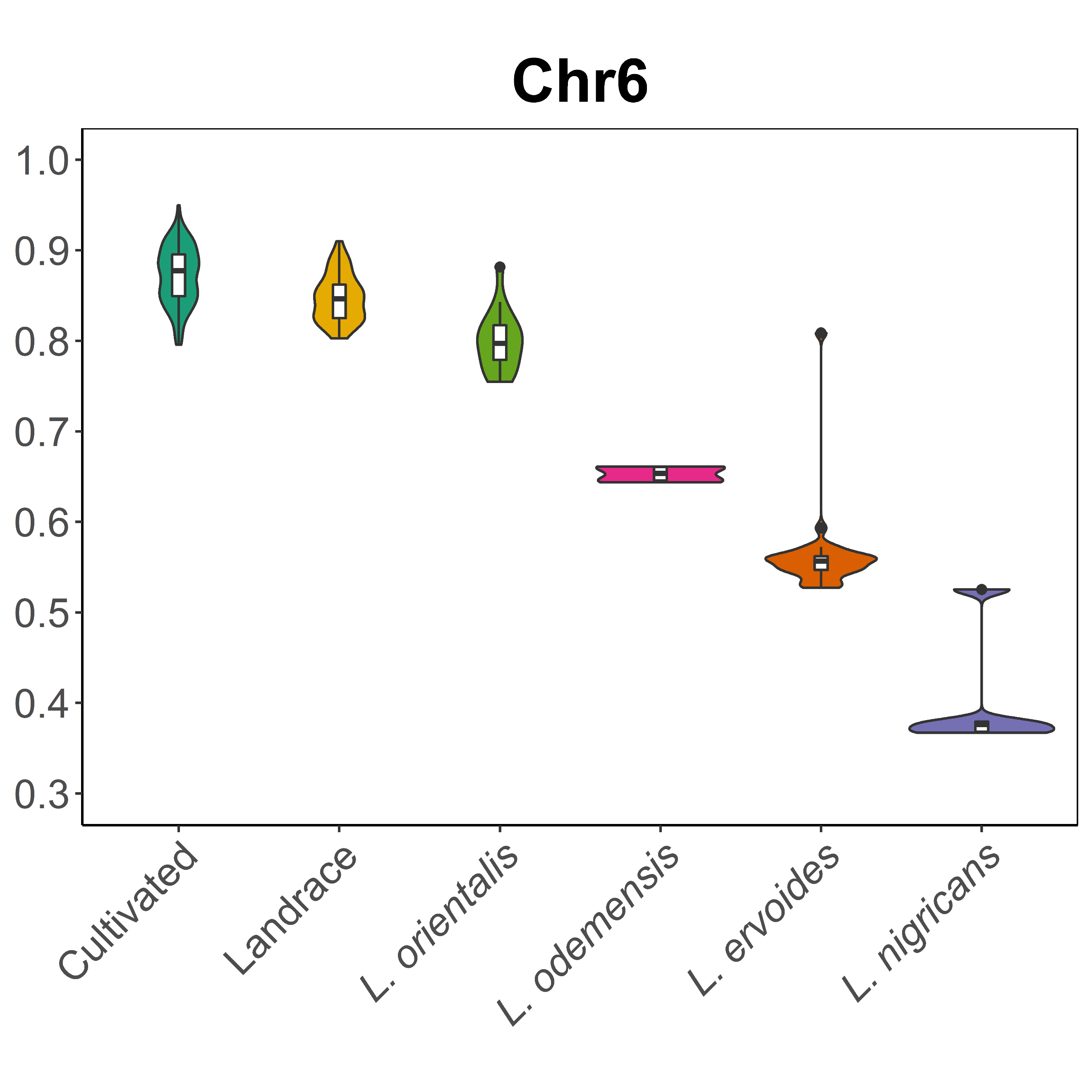

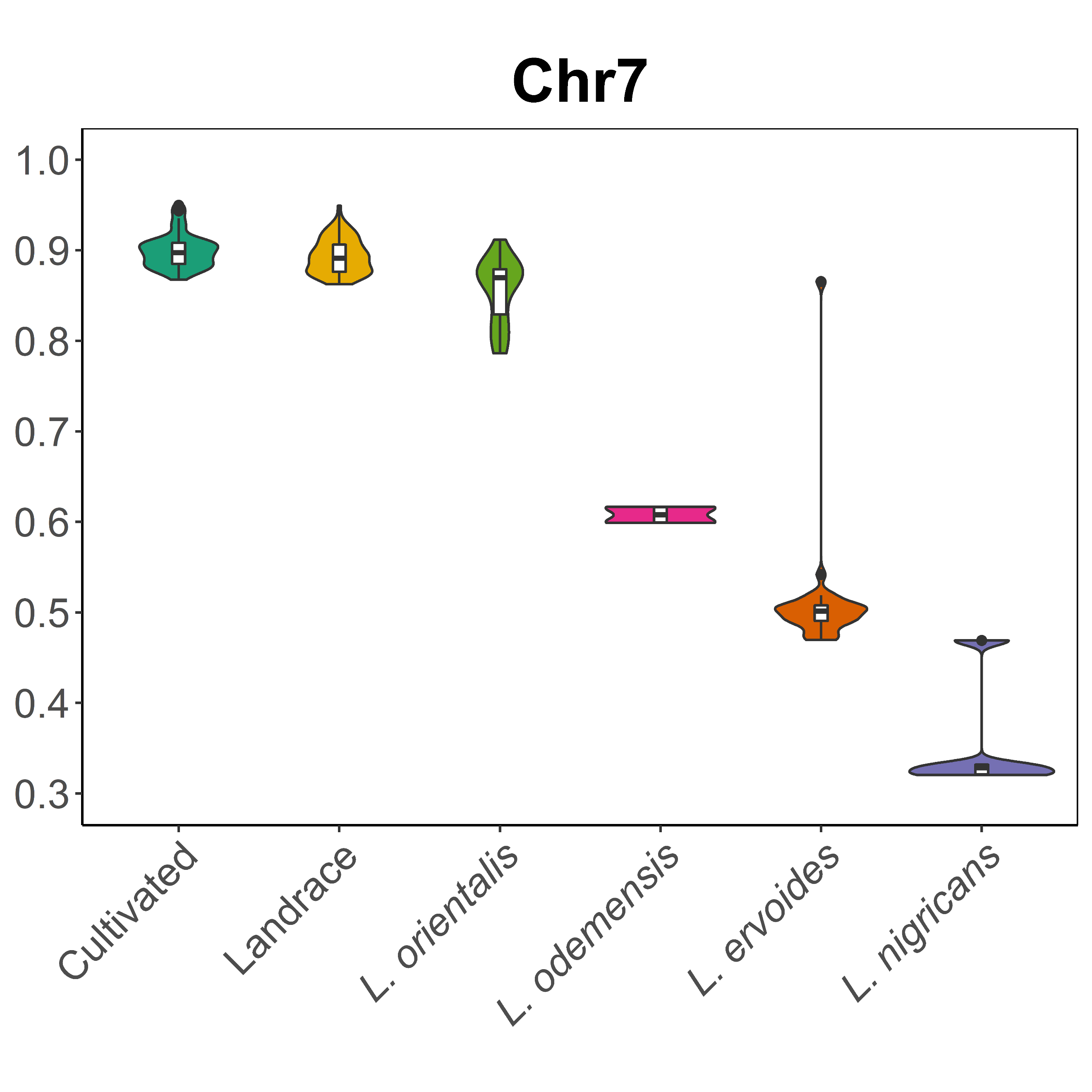


**Figure S1** Violin plots of the coverage of each group across each chromosome of the lentil genome.

b

a

**Figure S2** Venn diagram of unique and shared SNPs among different lentil groups. **a.** Among cultivated, landrace, *L. orientalis*, *L. odemensis*, *L. ervoides*, and *L. nigricans*. **b.** Among cultivated, landrace, and wild species.

b

a

**Figure S3** Venn diagram of unique and shared indels among different lentil groups. **a.** Among cultivated, landrace, *L. orientalis*, *L. odemensis*, *L. ervoides*, and *L. nigricans*. **b.** Among cultivated, landrace, and wild species.

**Figure S4** A phylogenetic tree inferred the evolutionary relationship of five lentil species. The gene pool classificaiton is indicated next to species name.


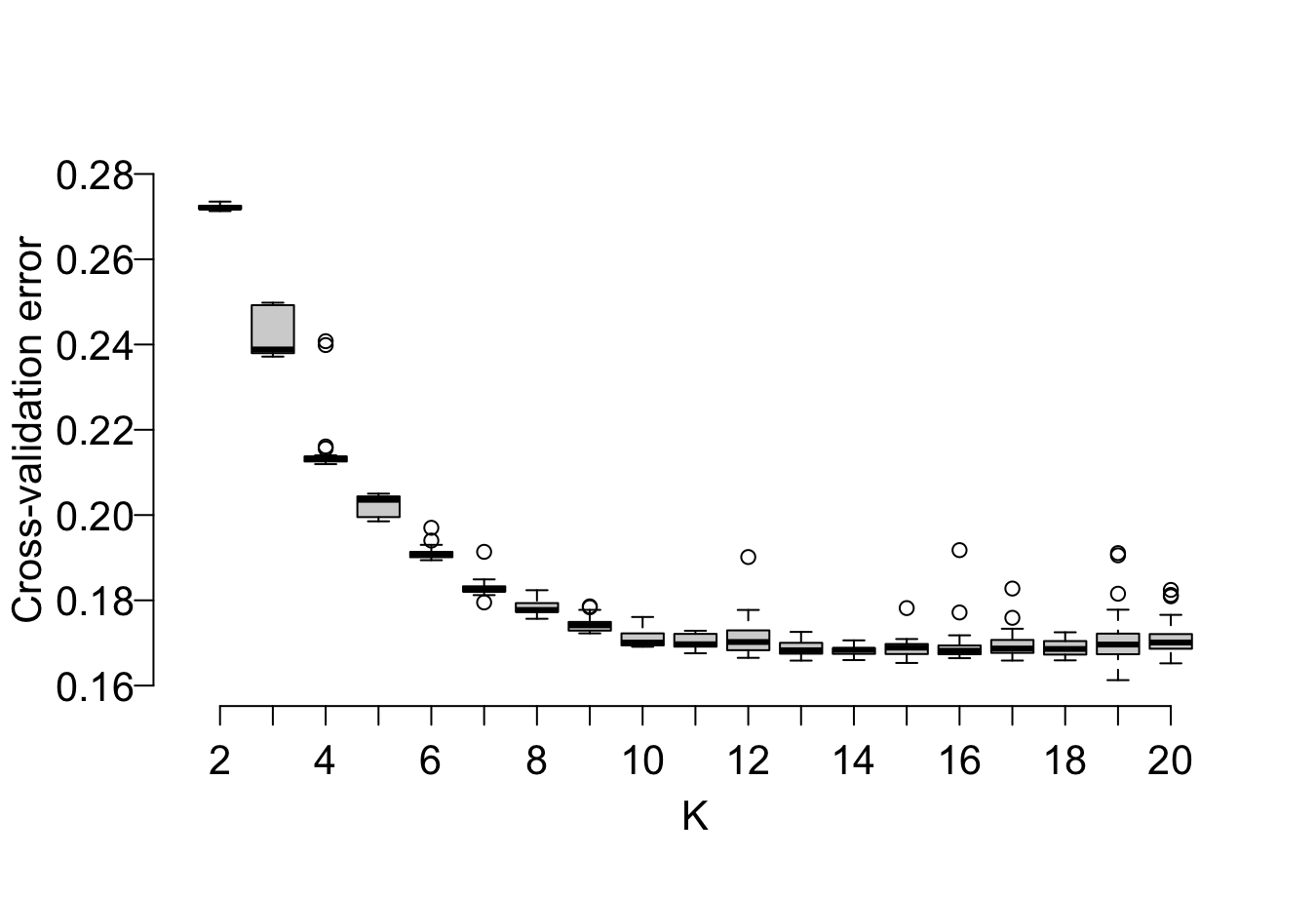
 **
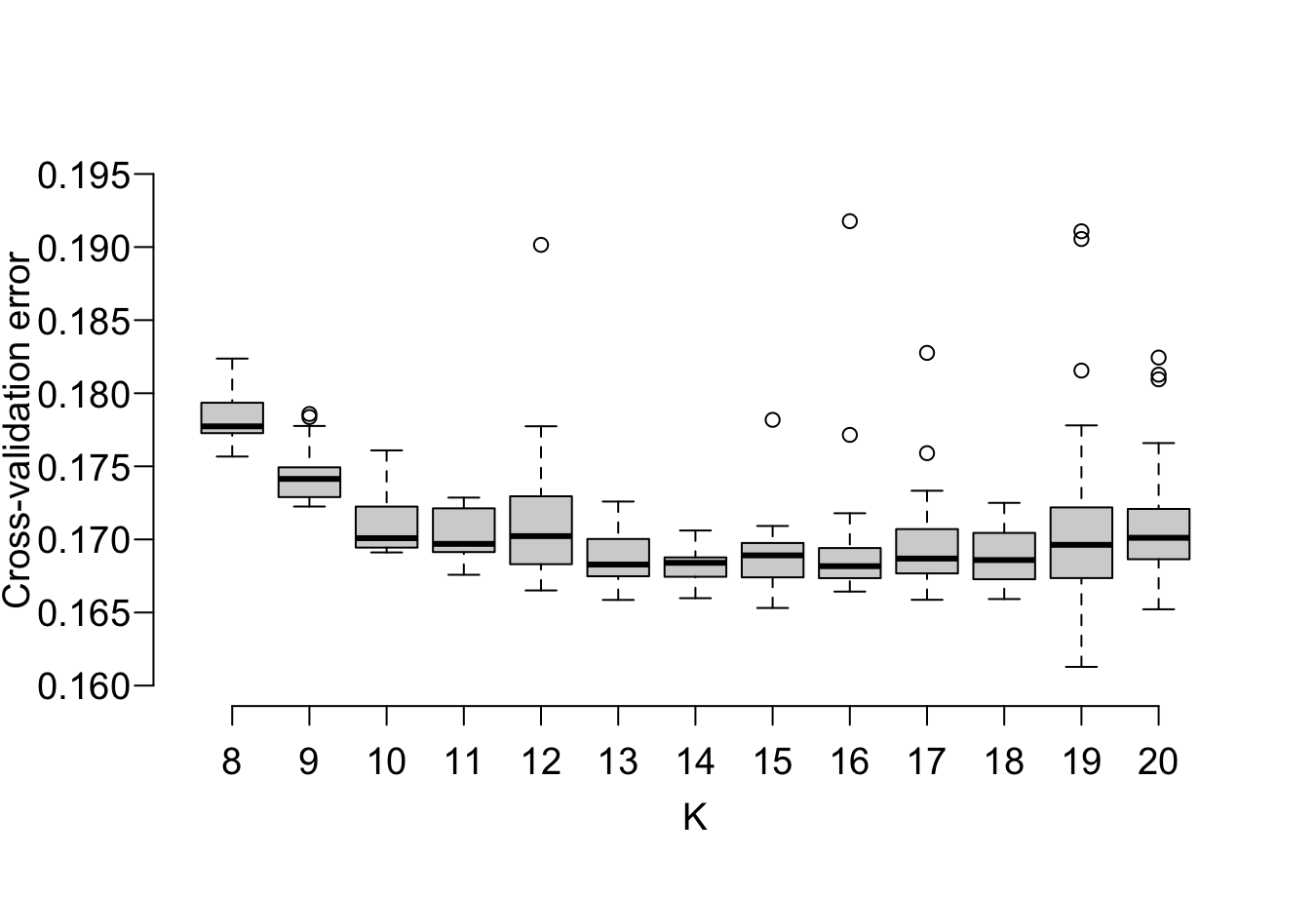
 Figure S5** Cross-validation errors for K values. **a.** Cross-validation errors for K from 2 to 20. **b.** Cross-validation errors for K from 8 to 20. Each box plot at each K indicates cross-validation errors calcualted from 20 independent runs. The median is represendted by the black line inside box plots. 25% and 75% percentiles are represnted by lower and upper hinges. Outliners are indicated by dots.

b

a

**
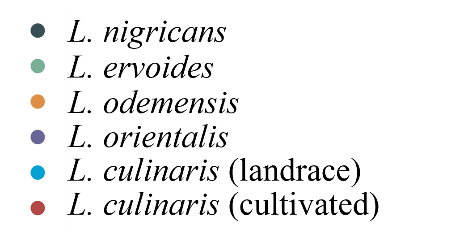

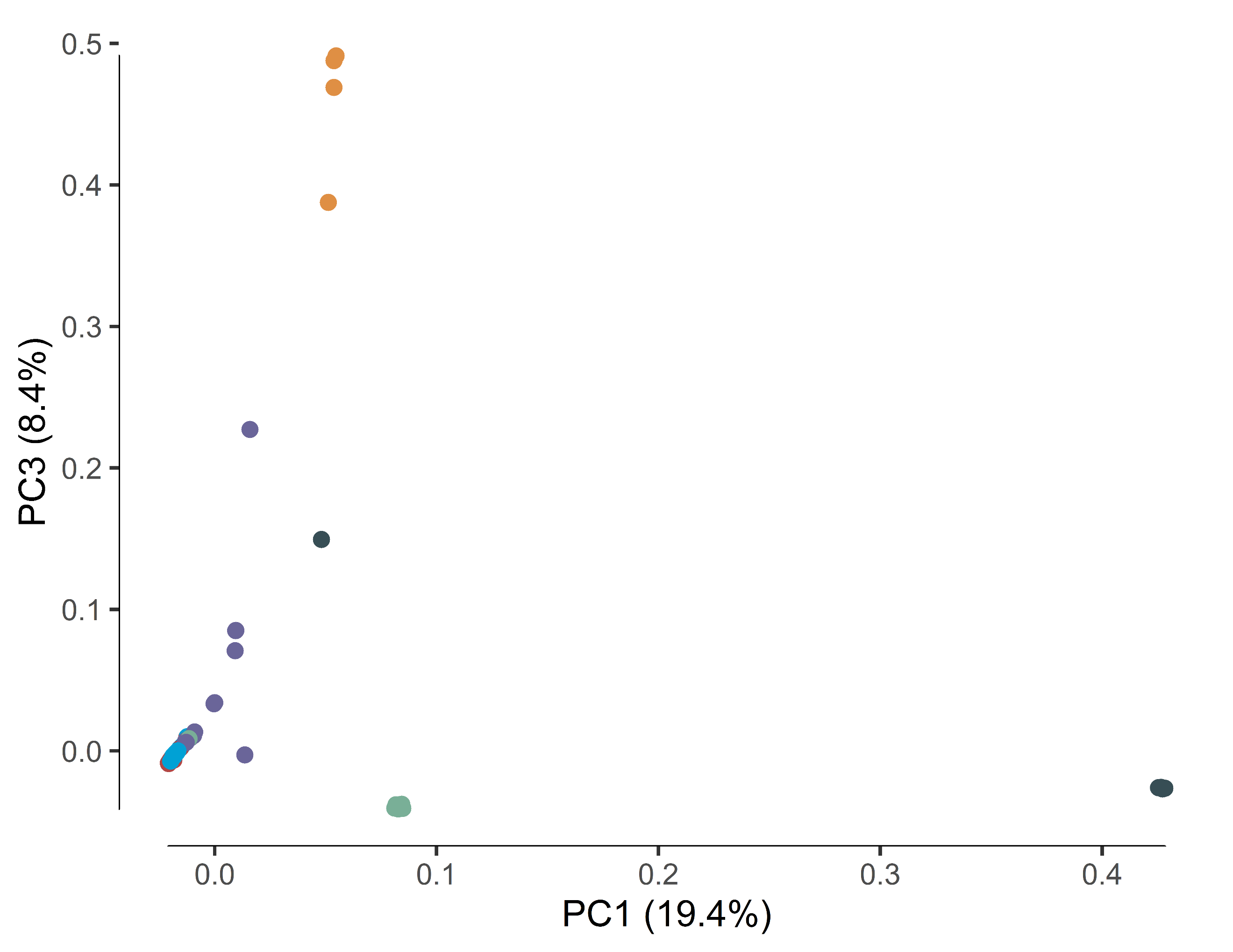

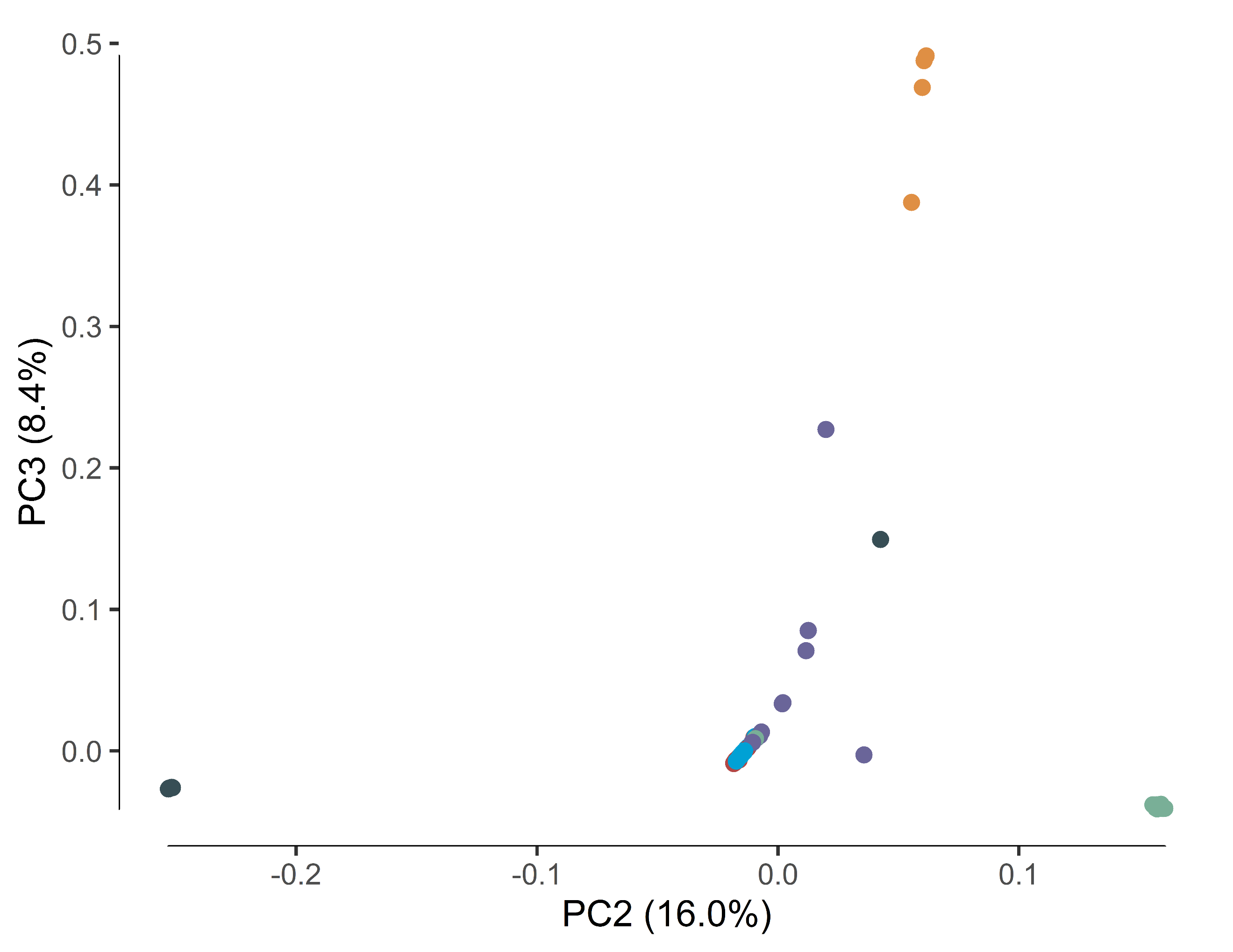
**

a

 **
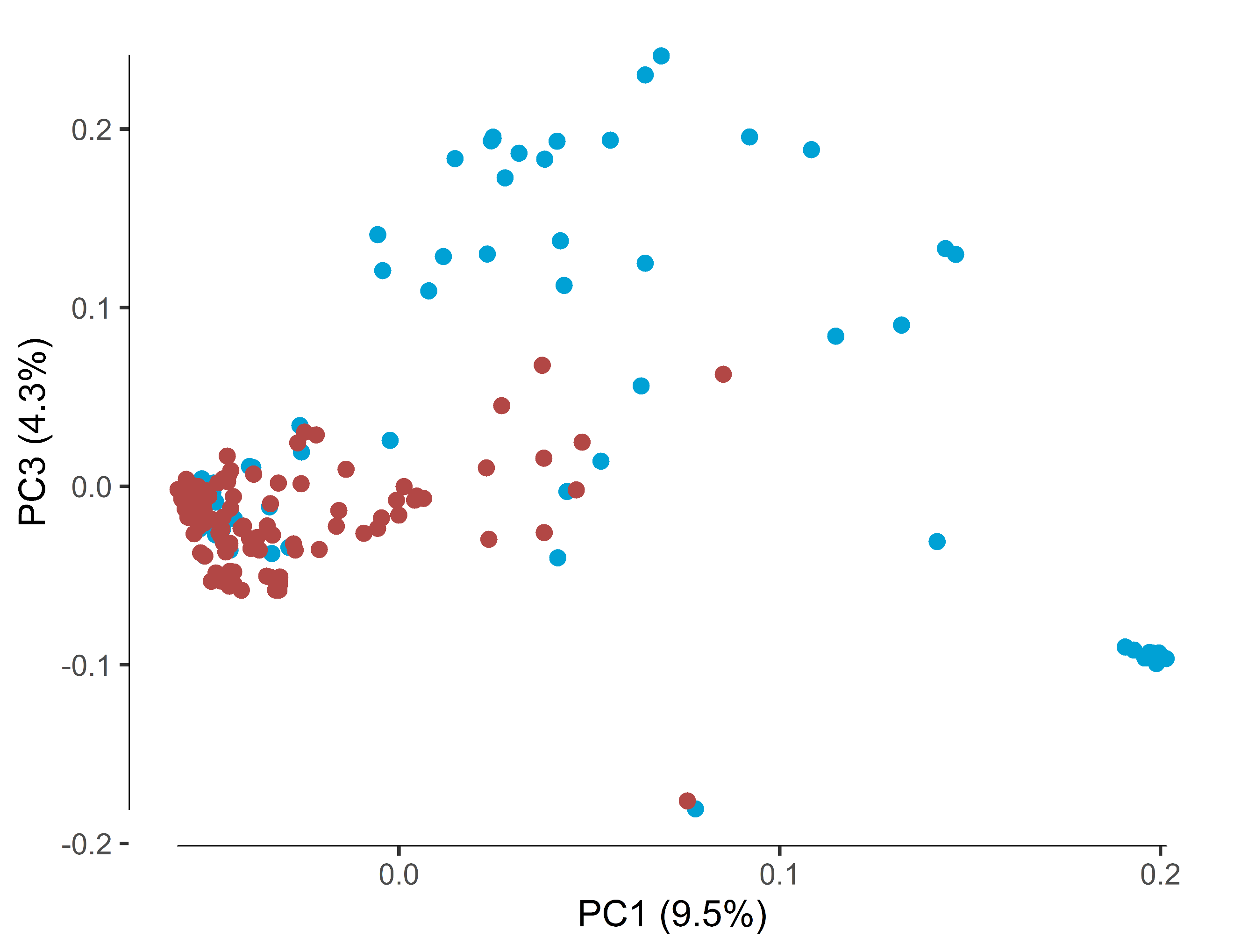

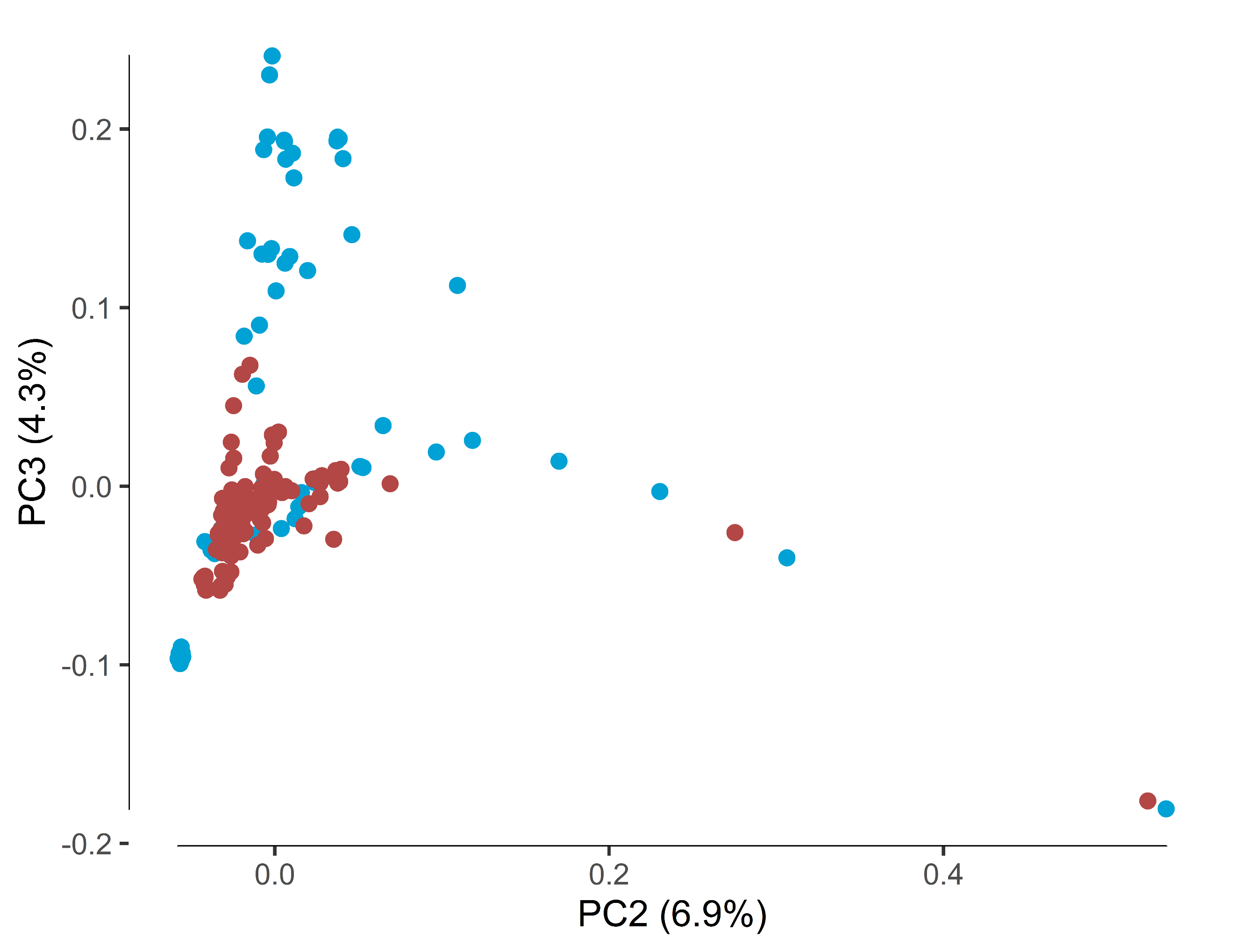
**

b

 **
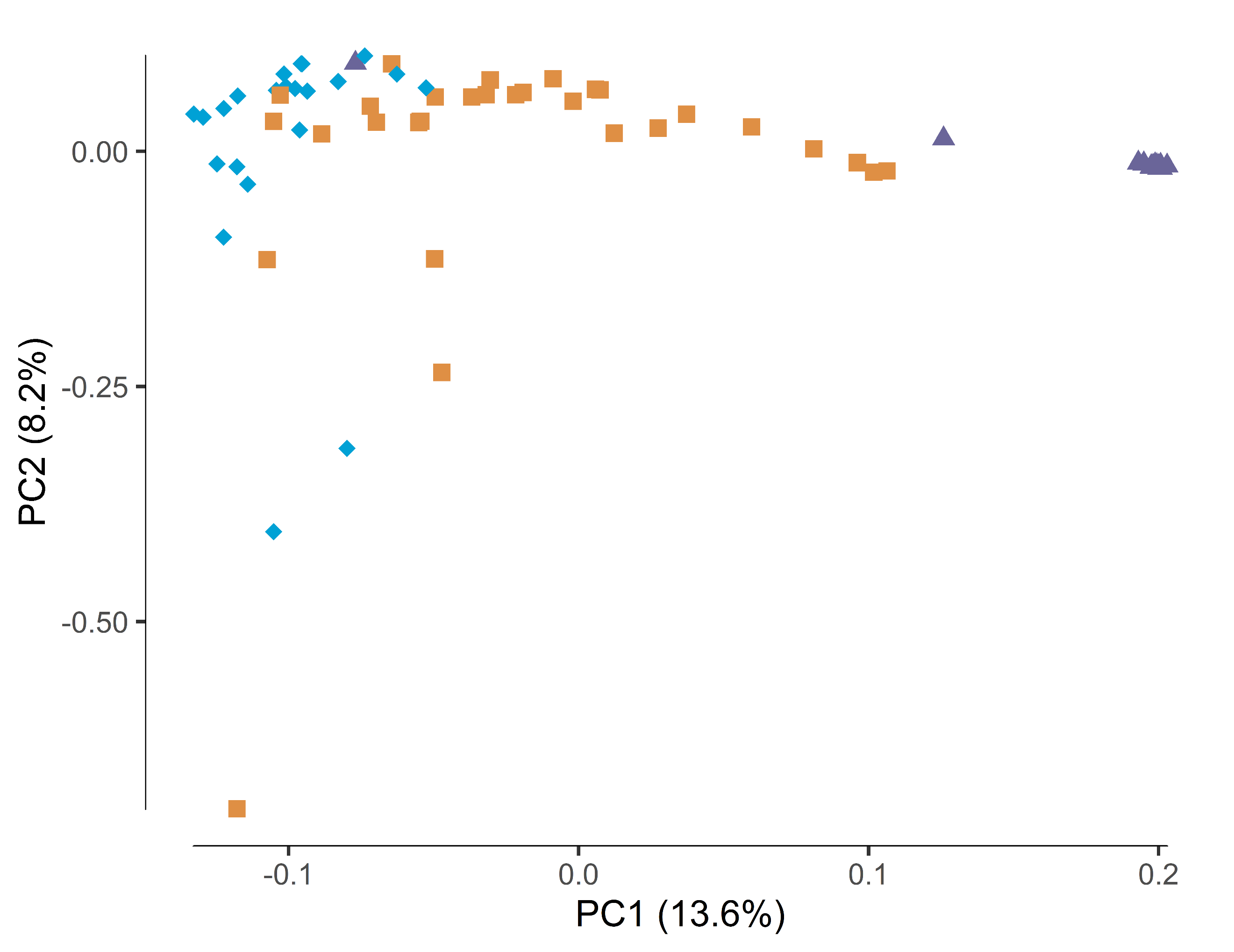

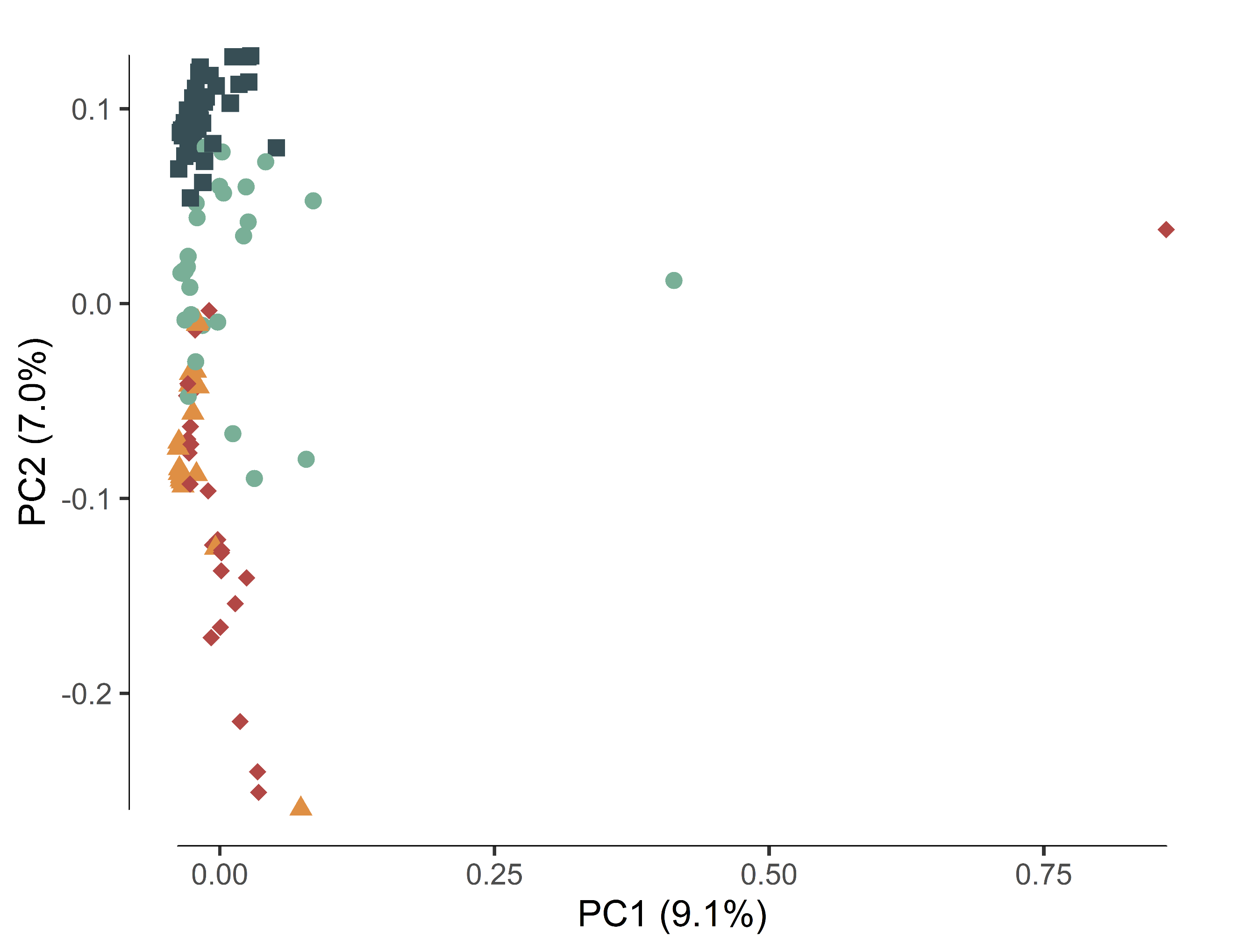
**

d

c

**Figure S6** Principle component analysis (PCA) plots for lentil accessions. **a.** PCA plots of the first three principal components for all 238 lentil accessions (PC1×PC3, PC2×PC3). **b.** PCA plots of the first three principal components for *L. culinaris* accessions (PC1×PC3, PC2×PC3). **c.** PCA plot of the first two principal components for *L. culinaris* landrace accessions. **d.** PCA plot of the first two principal components for *L. culinaris* cultivated accessions.

c

a

b

**Figure S7** Linkage disequilibrium (LD) decay among different lentil groups. **a.** four lentil groups. **b.** three phylogegraphic groups. **c.** four lentil market types.


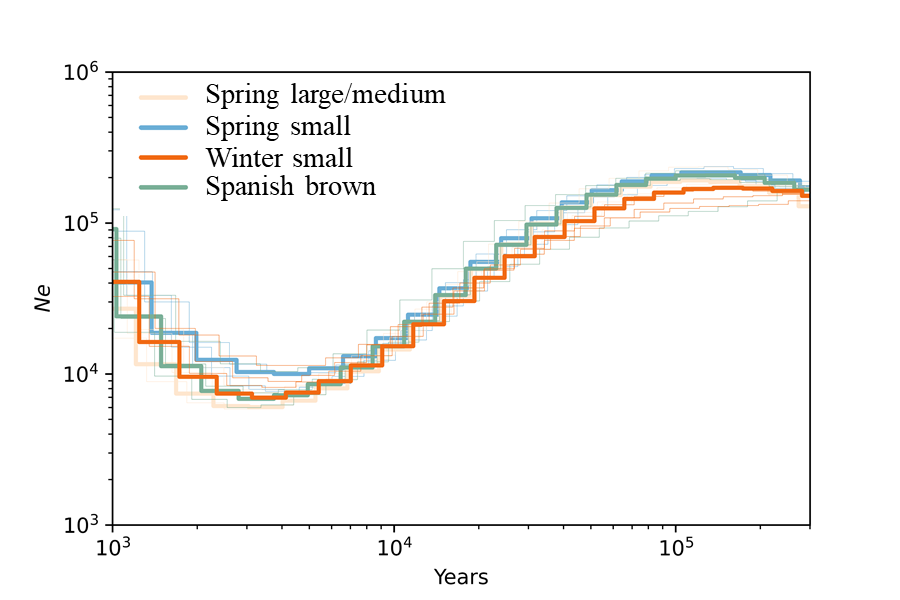

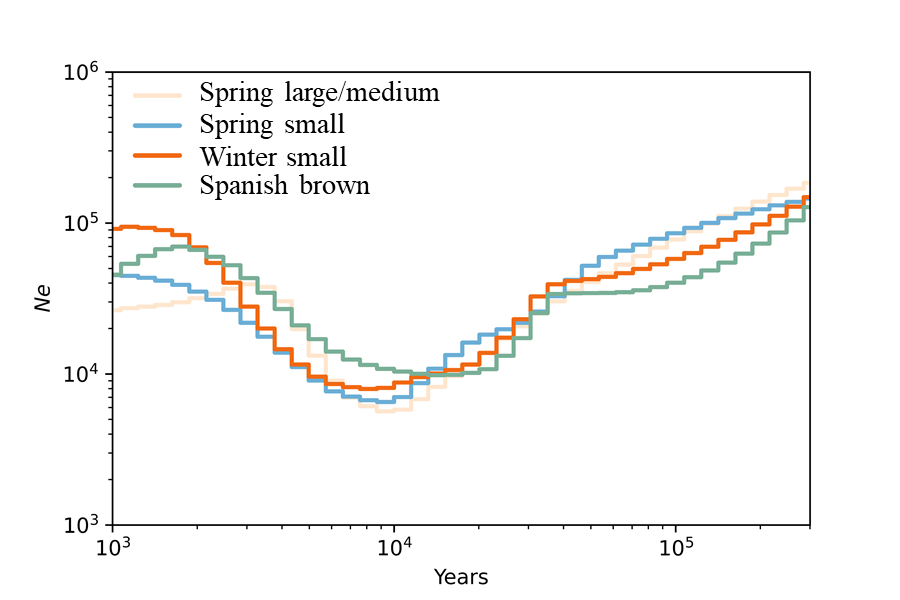


d

c

b

a

SMC++, *L. culinaris* (landrace)

MSMC2, *L. culinaris* (cultivated)

SMC++, *L. culinaris* (cultivated)

MSMC2, *L. culinaris* (landrace)

**Figure S8** Demographic history of *L. culinaris* (landrace) and *L. culinaris* (cultivated). **a.** MSMC2-derived demographic history of three phylogeographic groups. **b.** SMC++-derived demographic history of three phylogeographic groups. **c.** MSMC2-derived demographic history of four market type groups. **d.** SMC++-derived demographic history of four market type groups. MSMC2 was performed on randomly selected three samples from each group and repeated four times. SMC++ was performed on randomly selected 10 samples. The generation time was set as one year and the mutation rate per site per generation as 8.3 × 10^-9^.

**
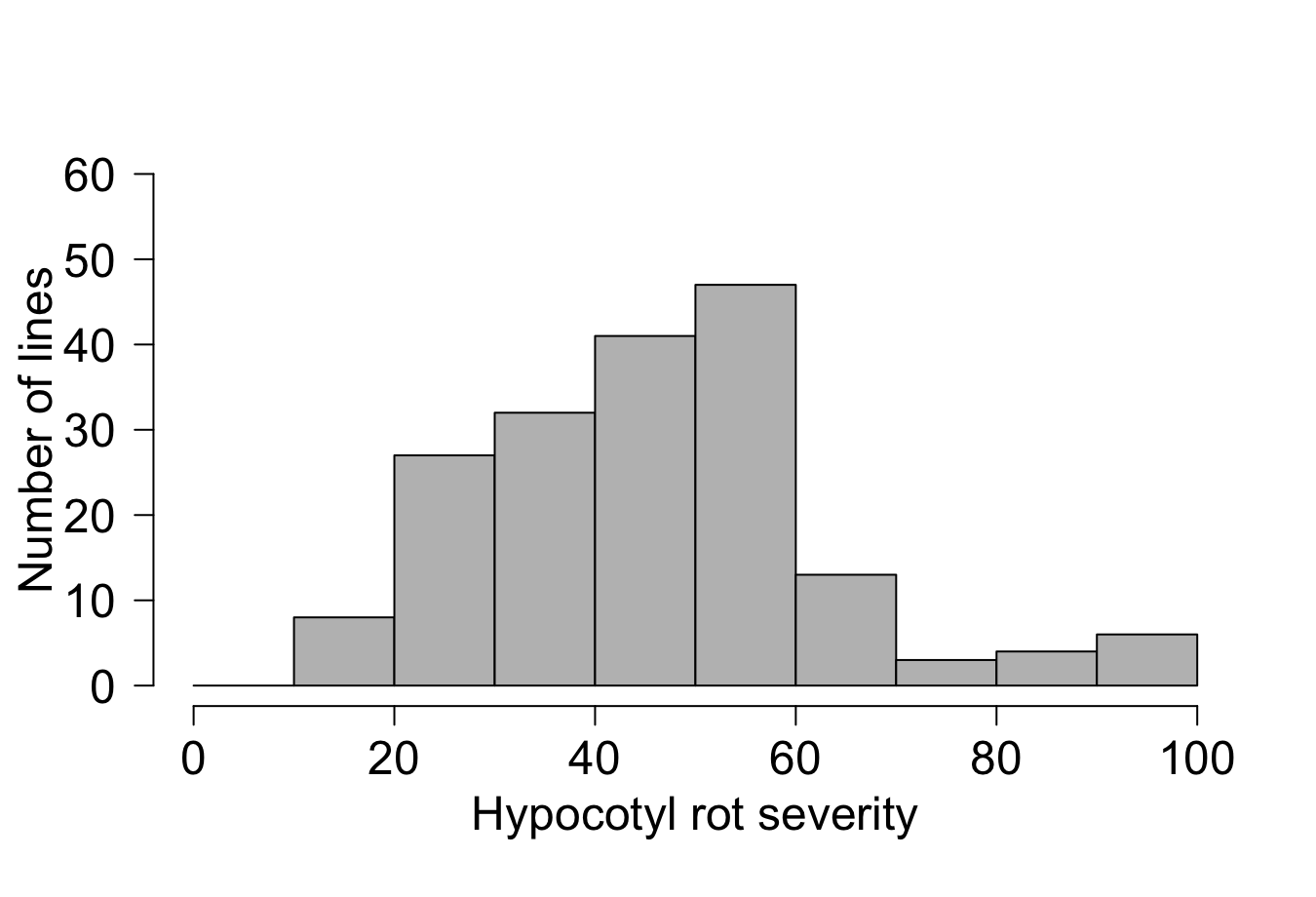

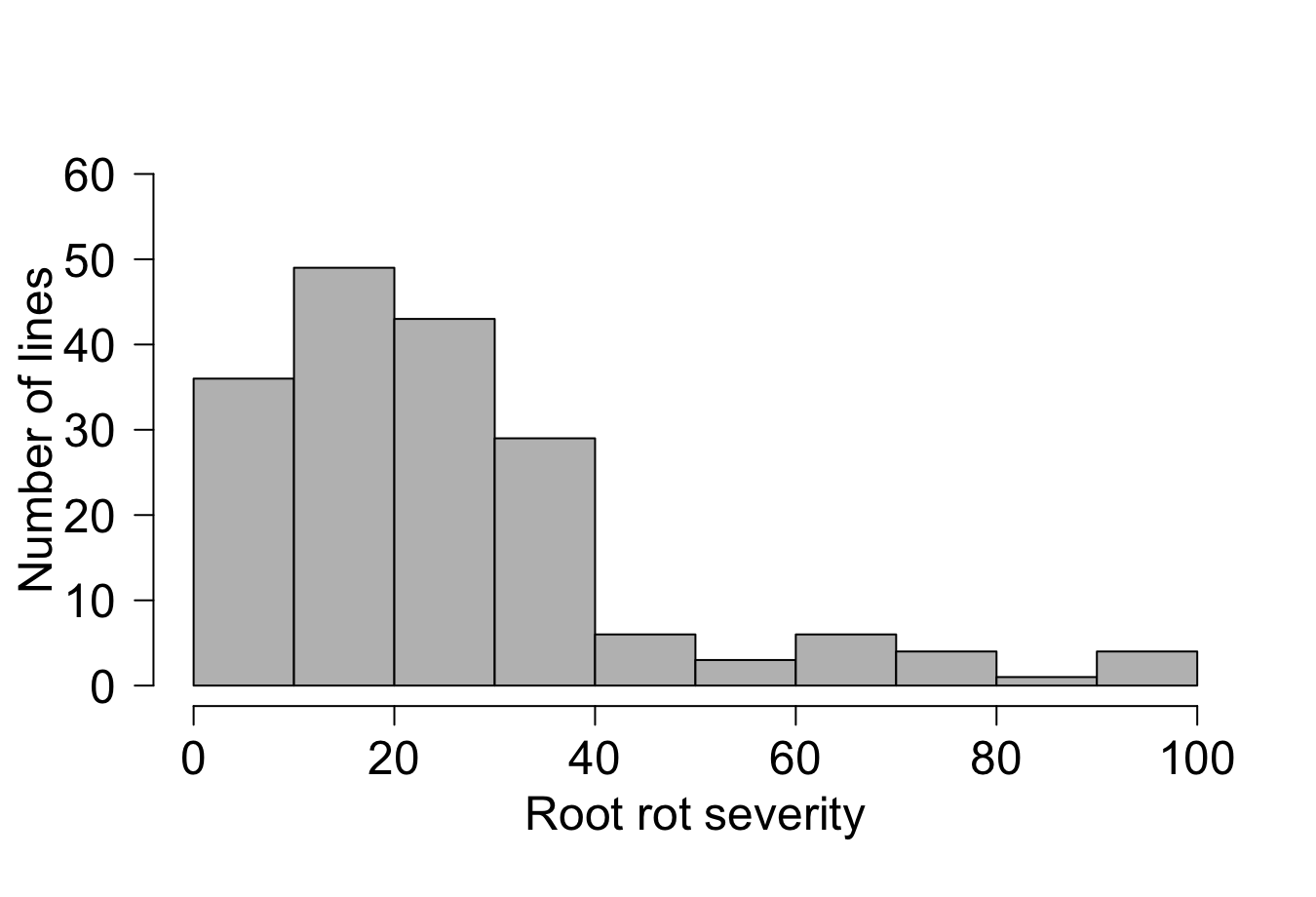
** **
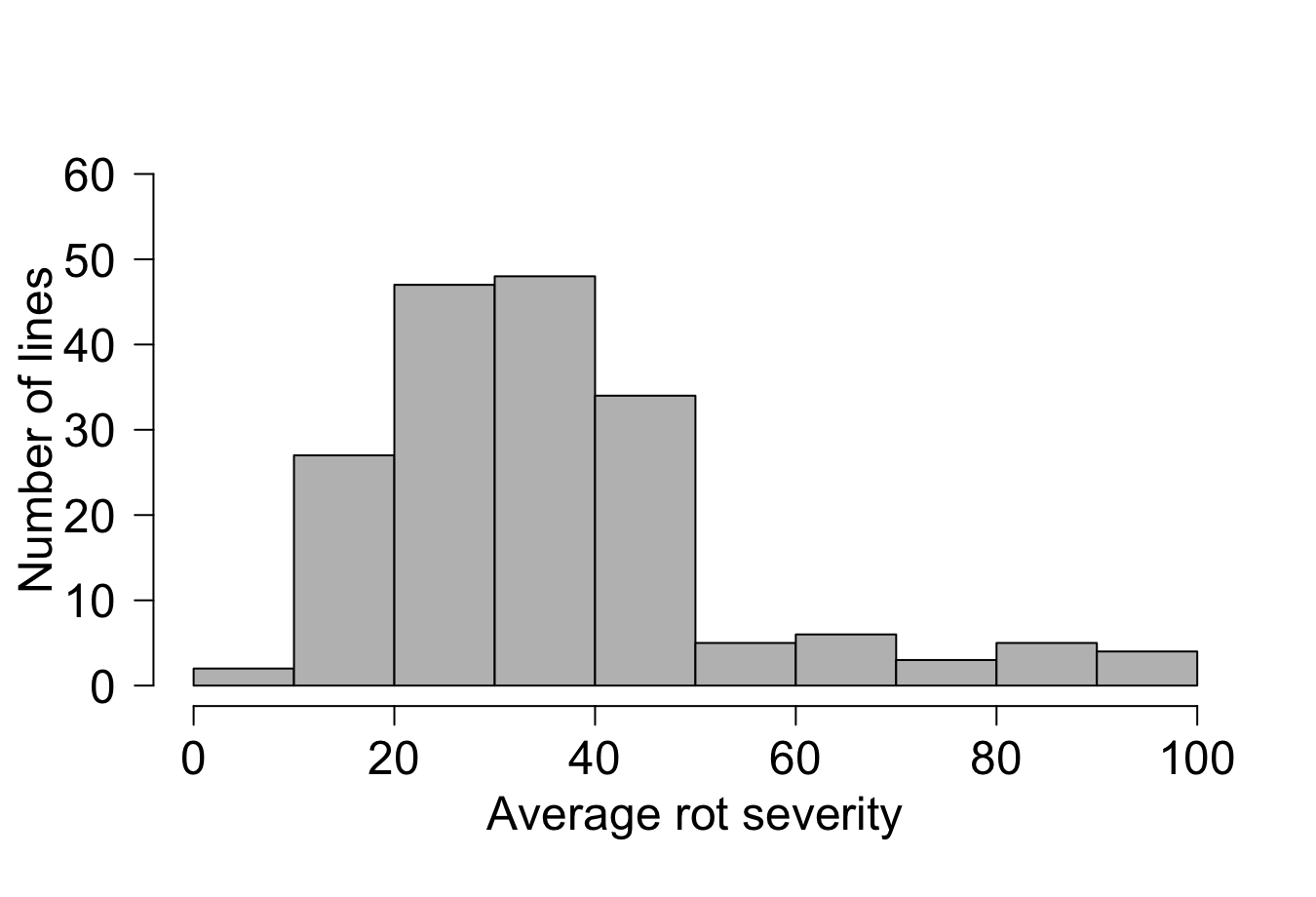
**
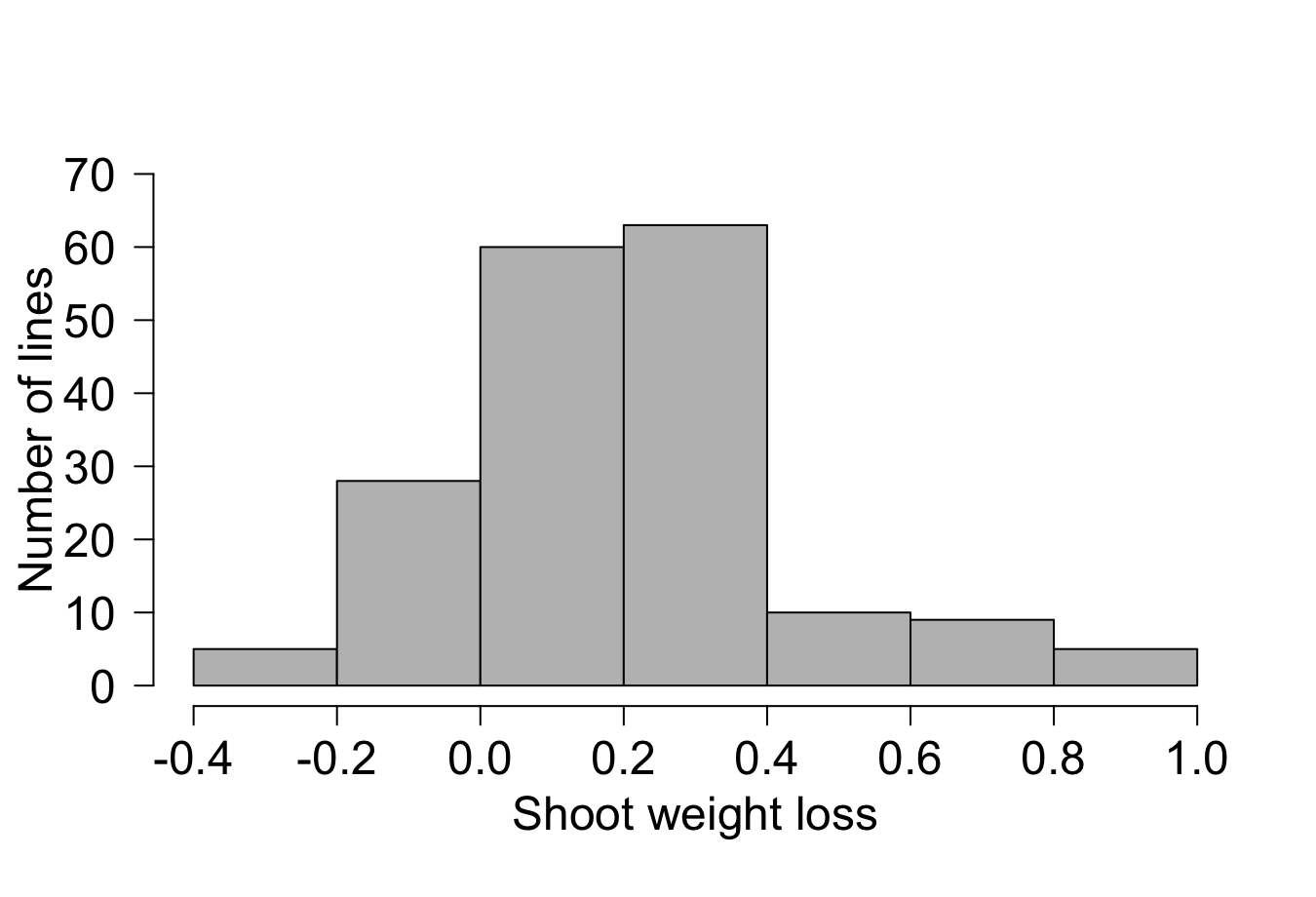


**
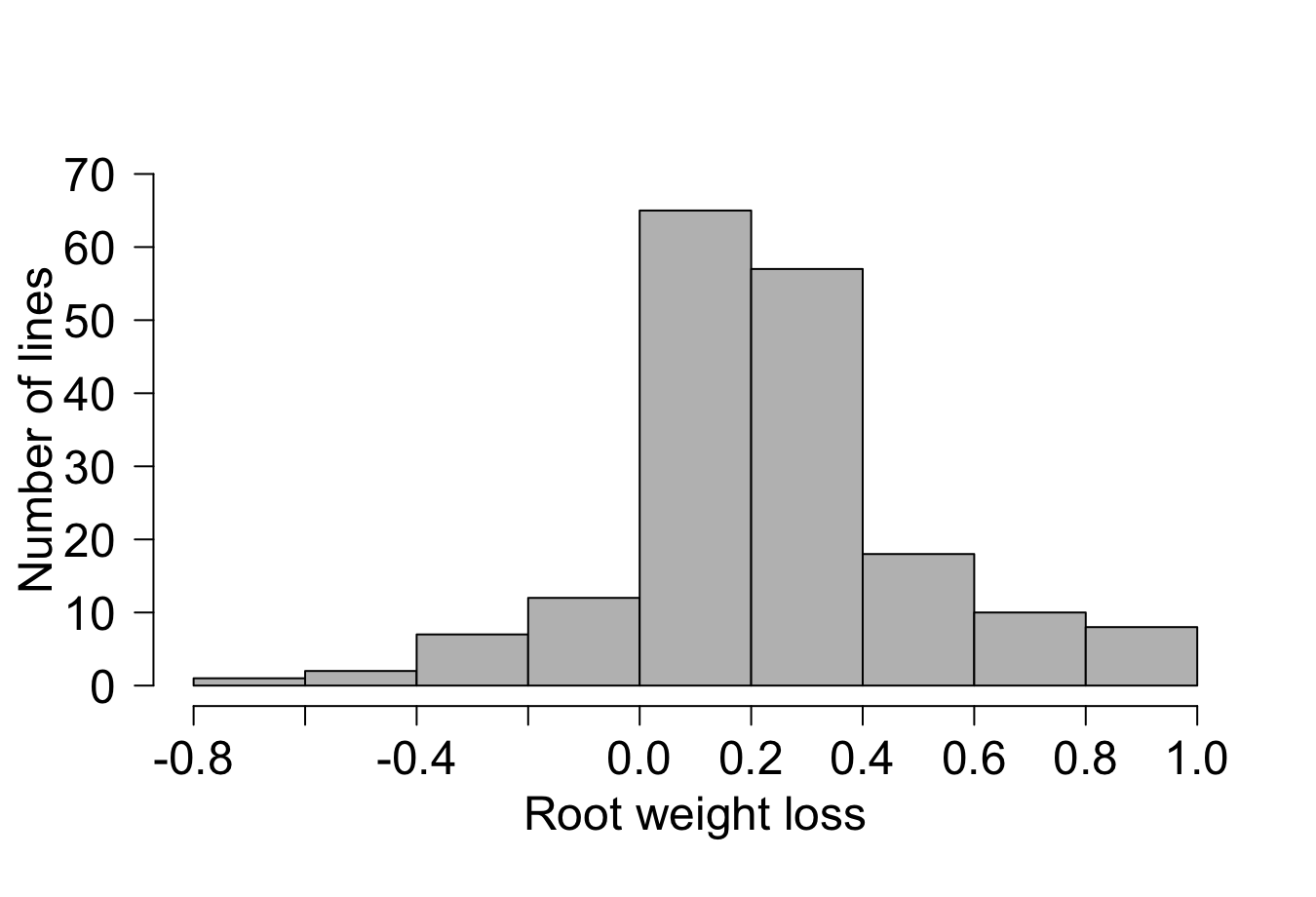
**  **
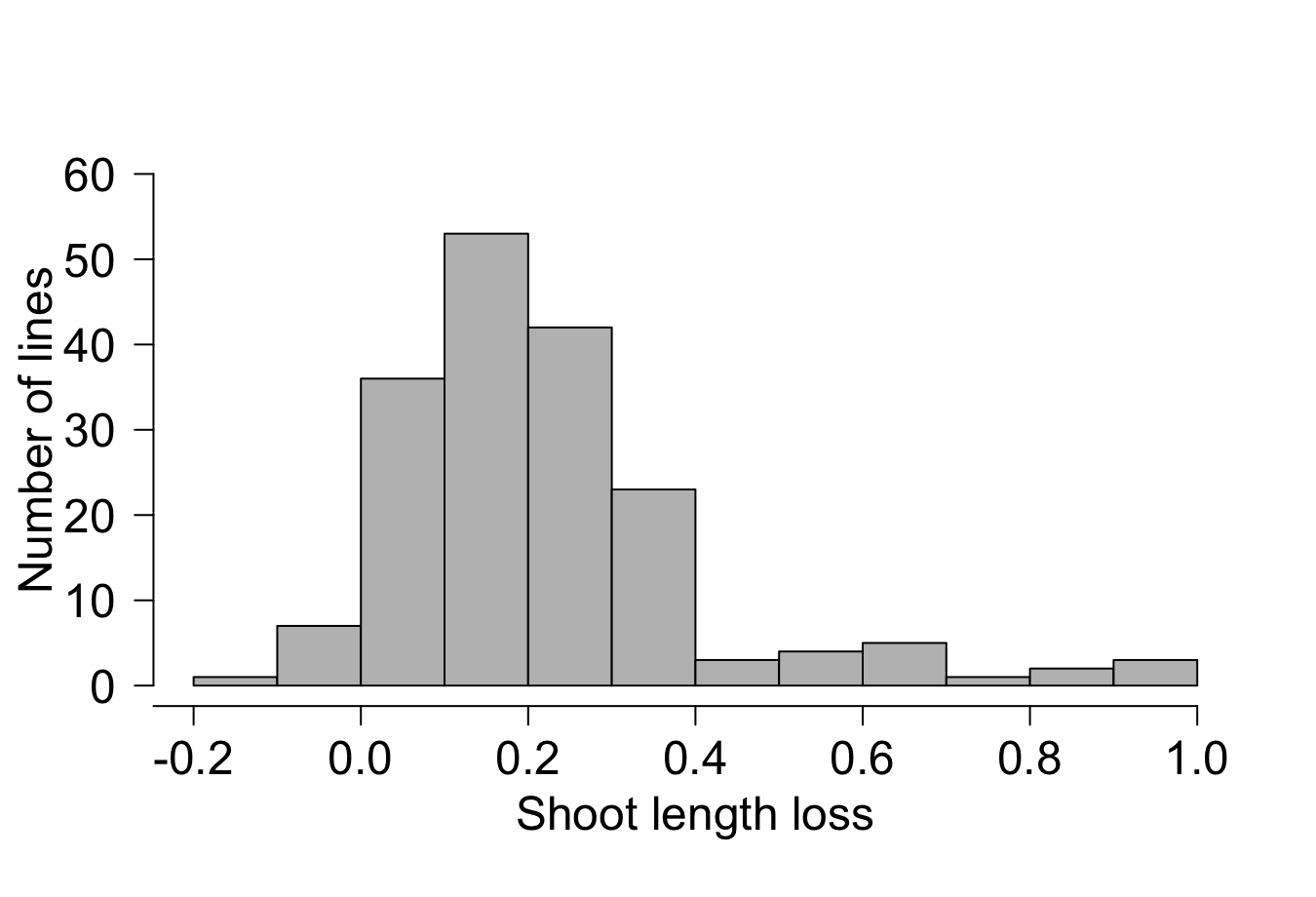
**

**Figure S9** Frequency distribution of phenotypic variation of six traits for Fusarium root rot (4 × 10^6^ spores/mL) in 183 *L. culinaris* landrace and cultivated accessions.


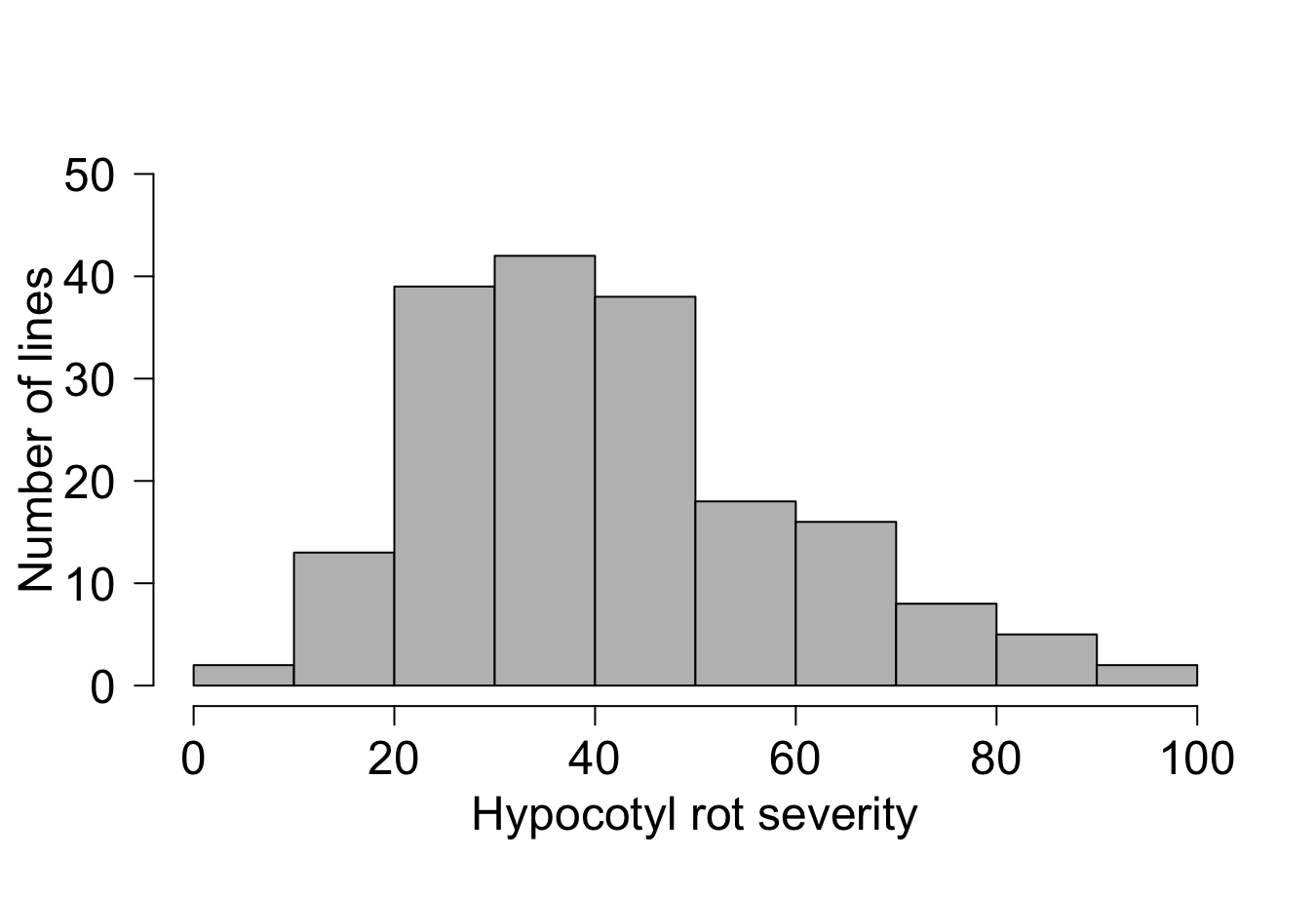

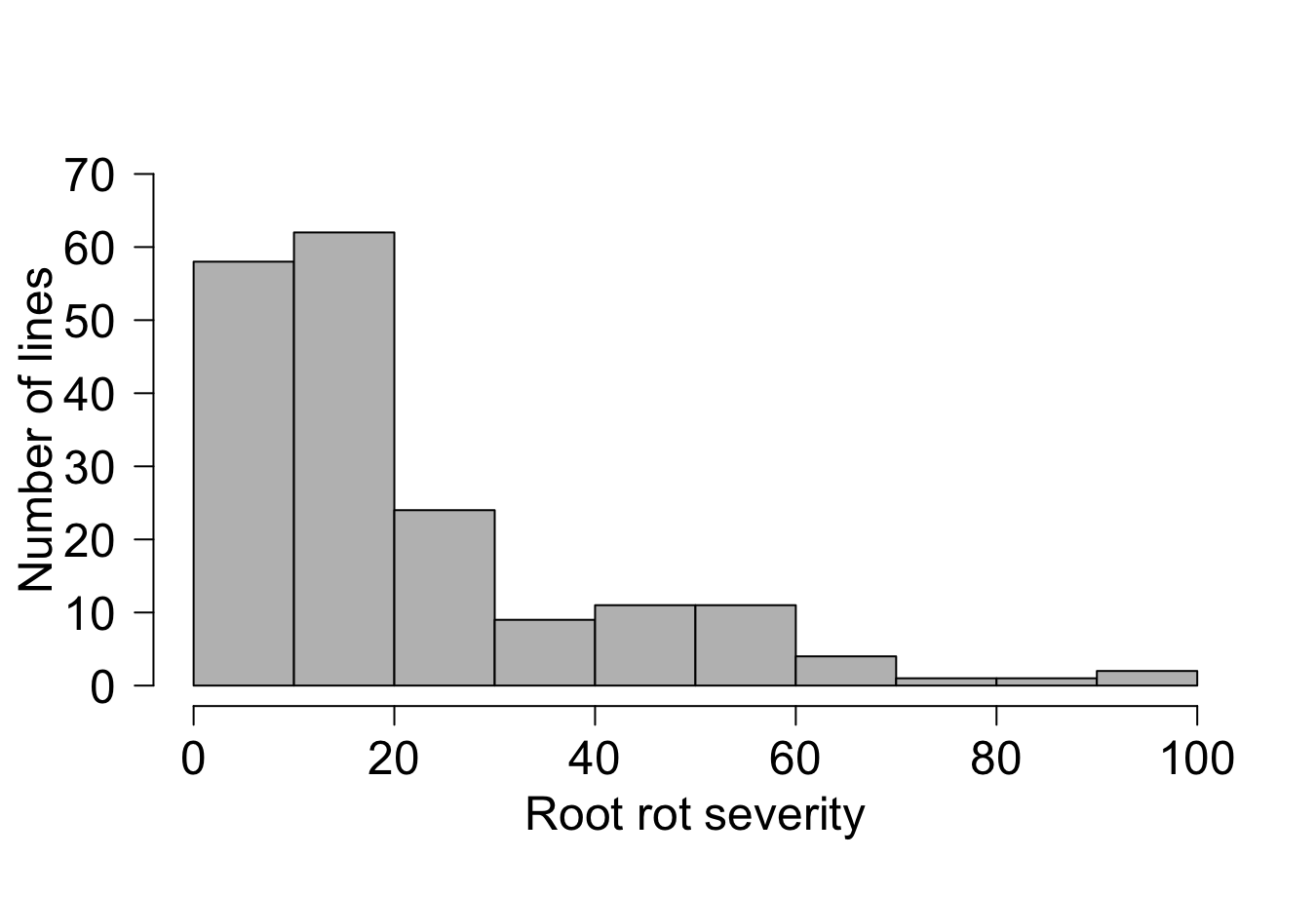


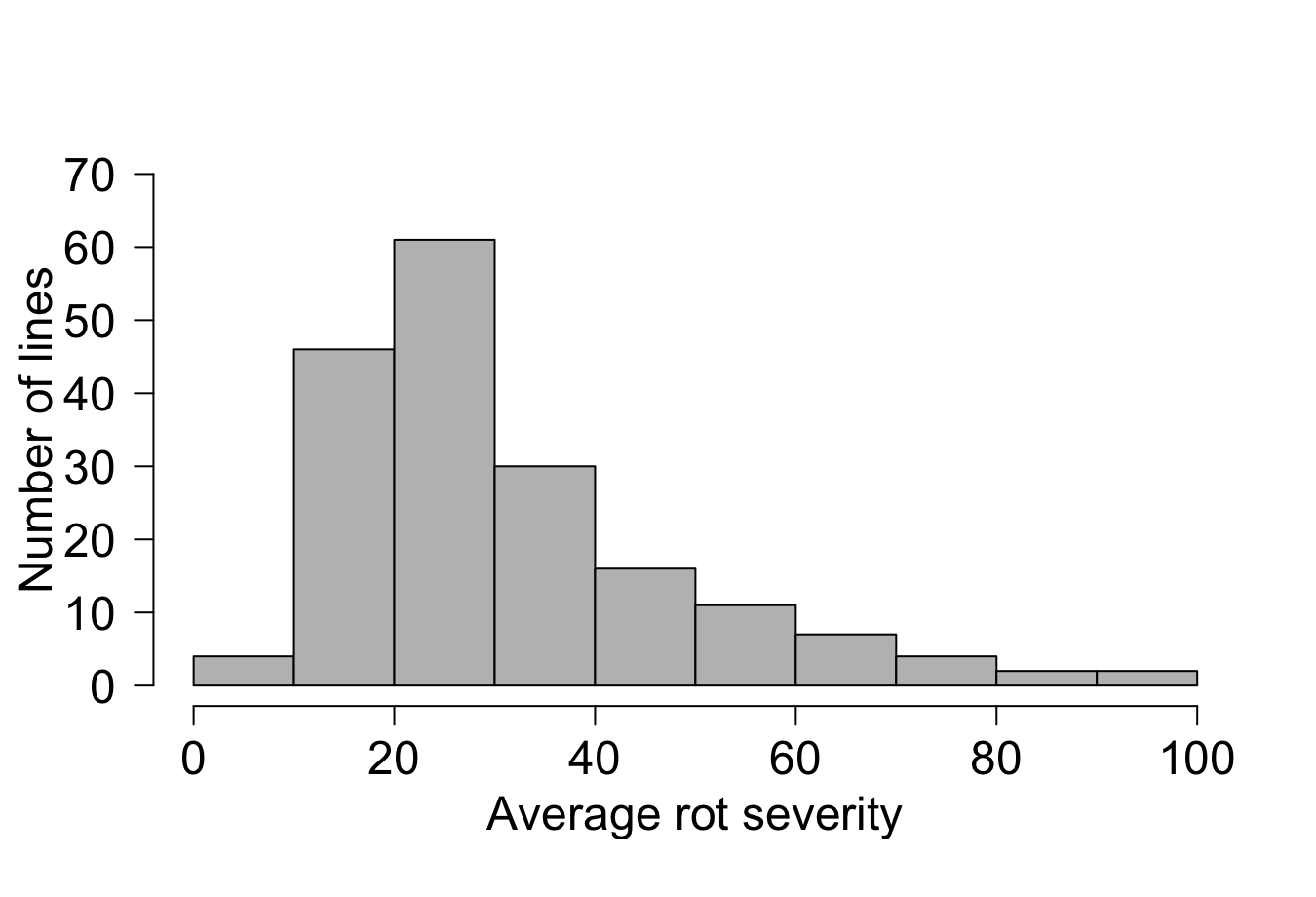

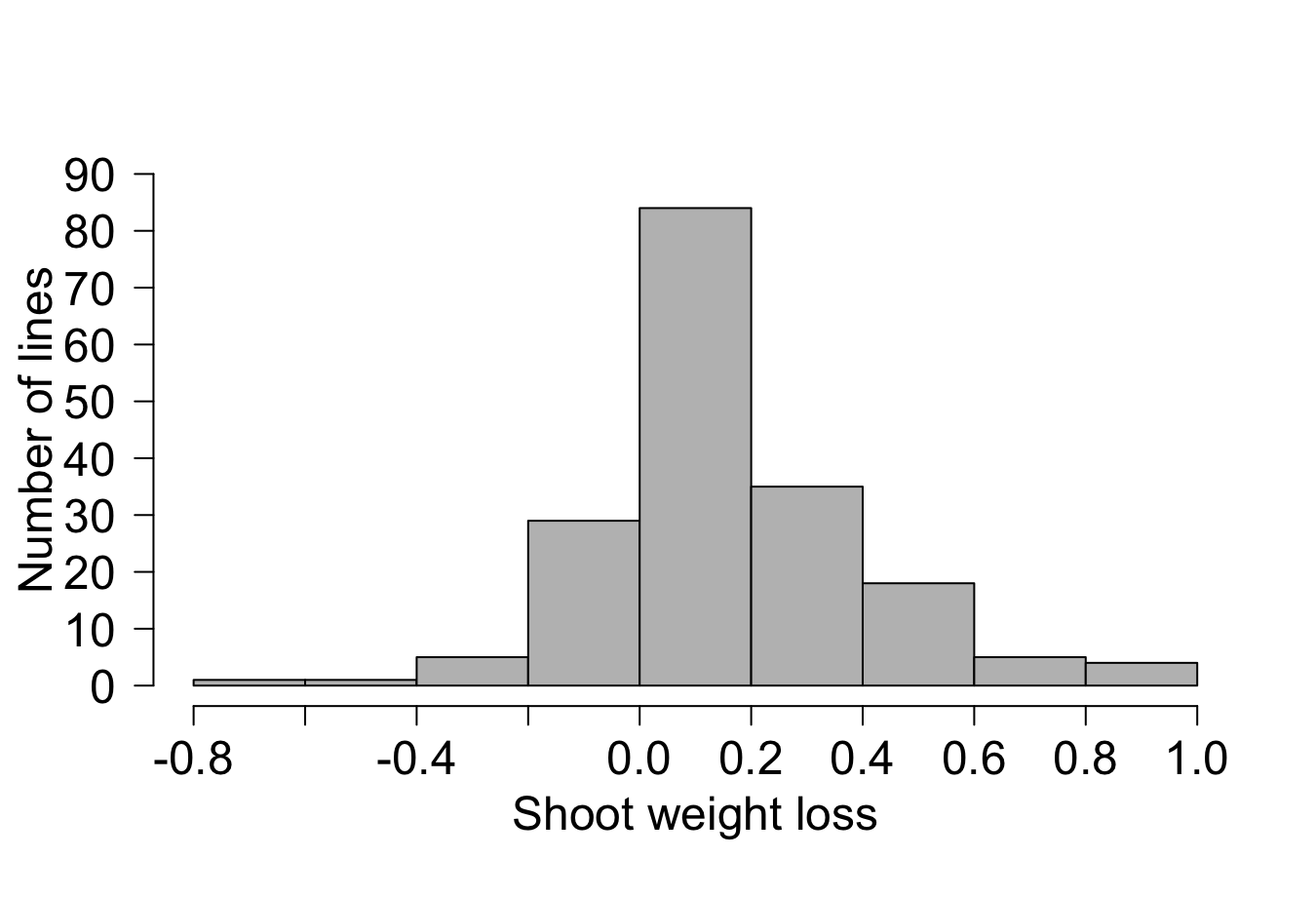


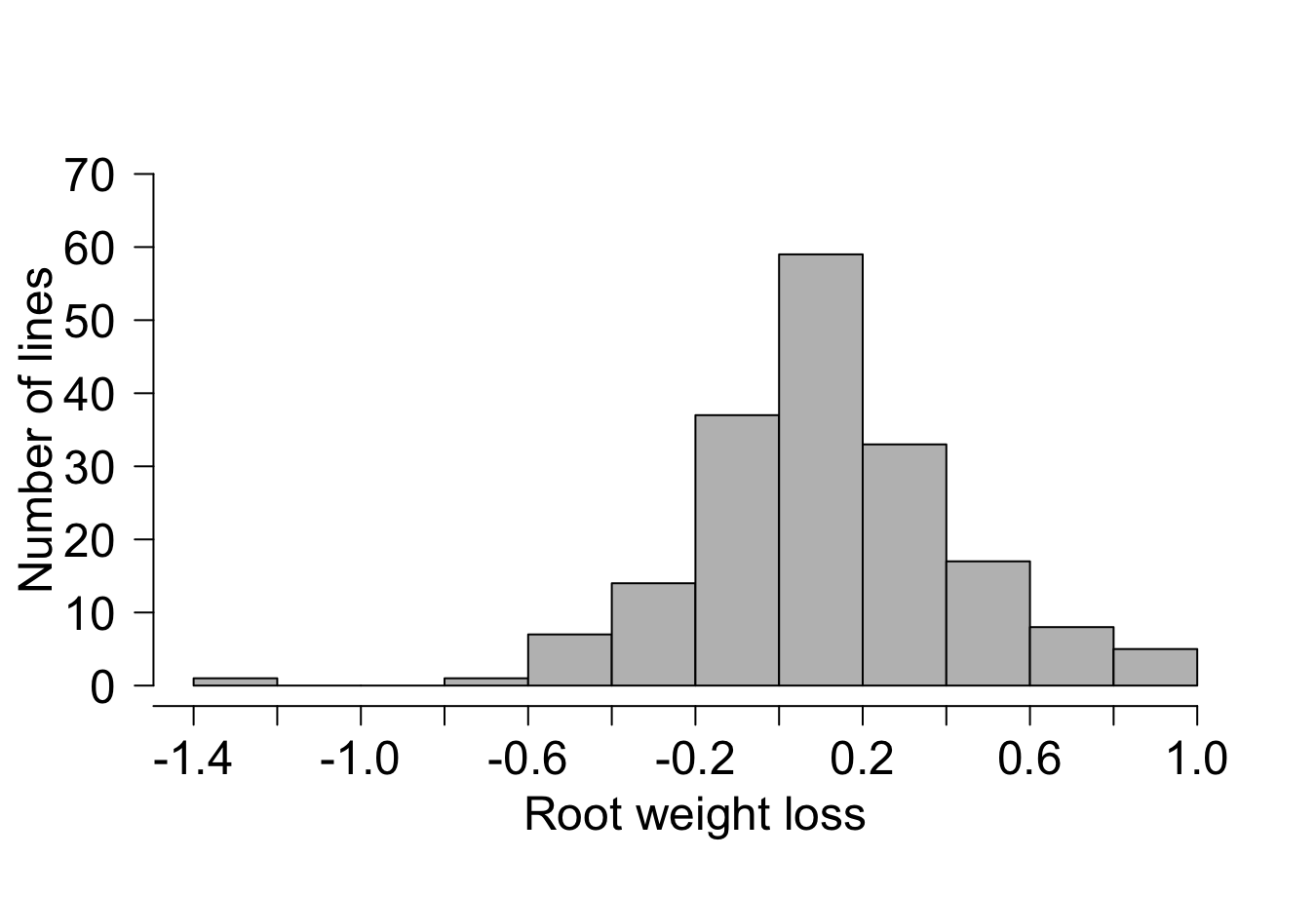

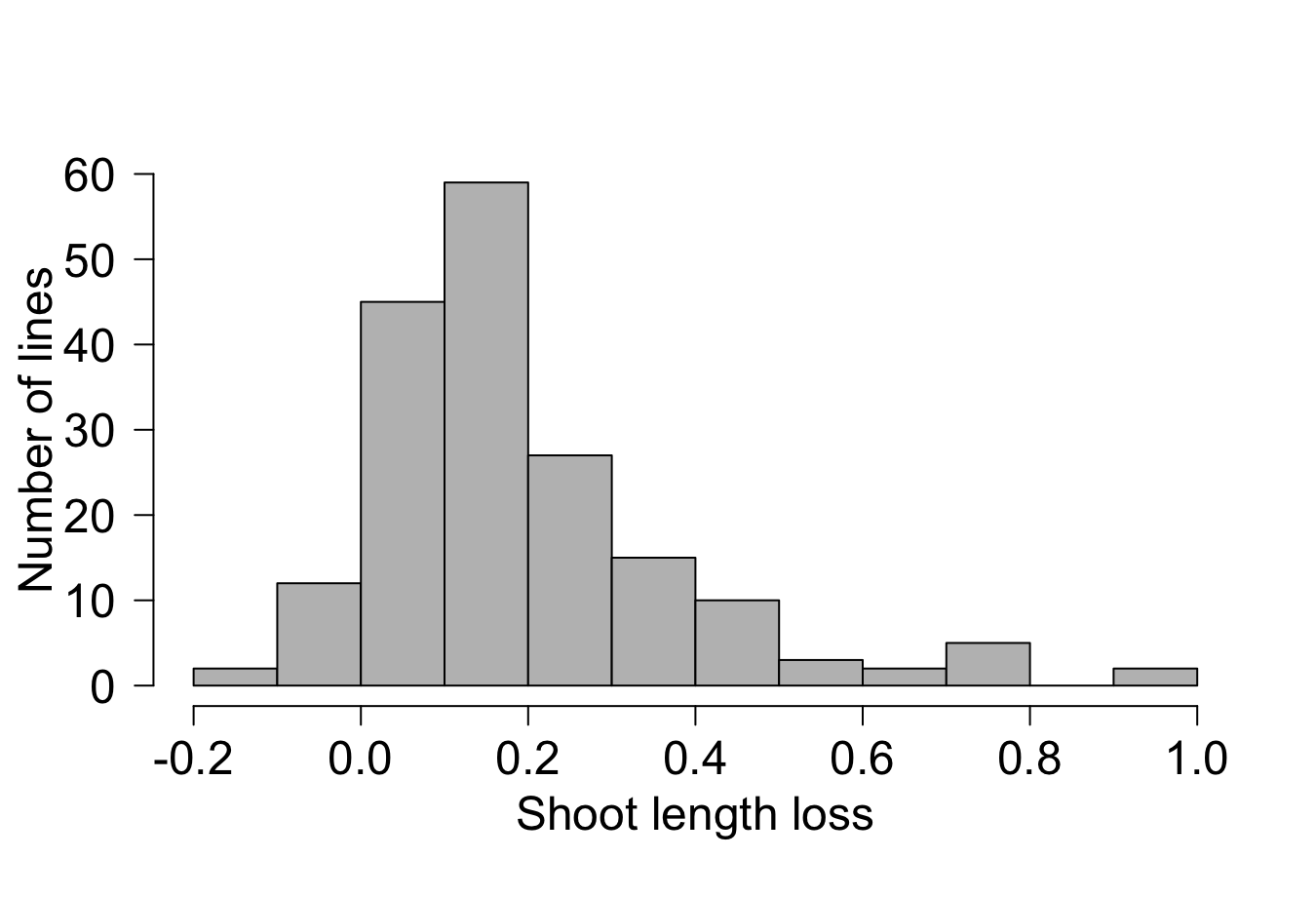


**Figure S10** Frequency distribution of phenotypic variation of six traits for Fusarium root rot (2 × 10^6^ spores/mL) in 183 *L. culinaris* landrace and cultivated accessions.


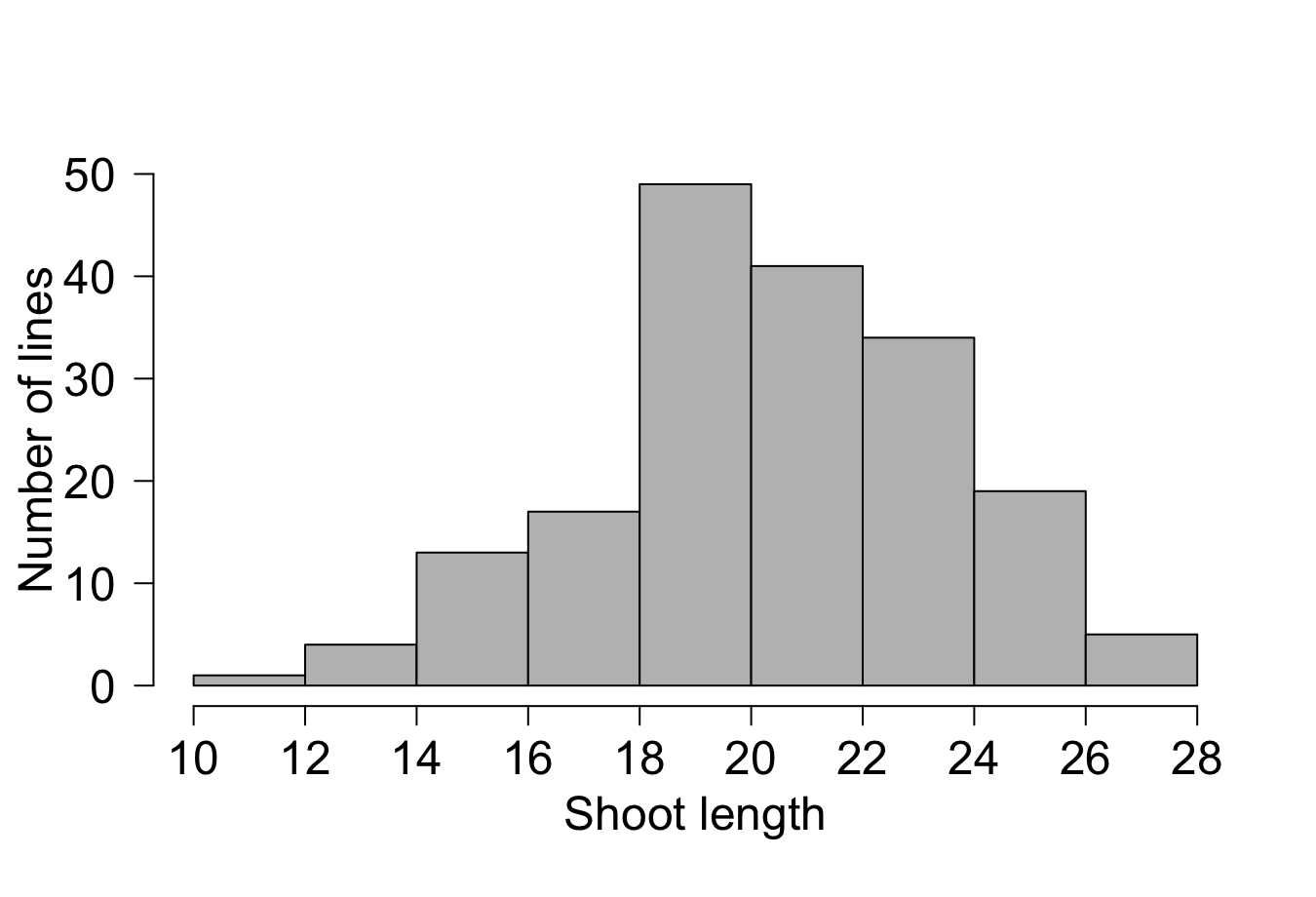

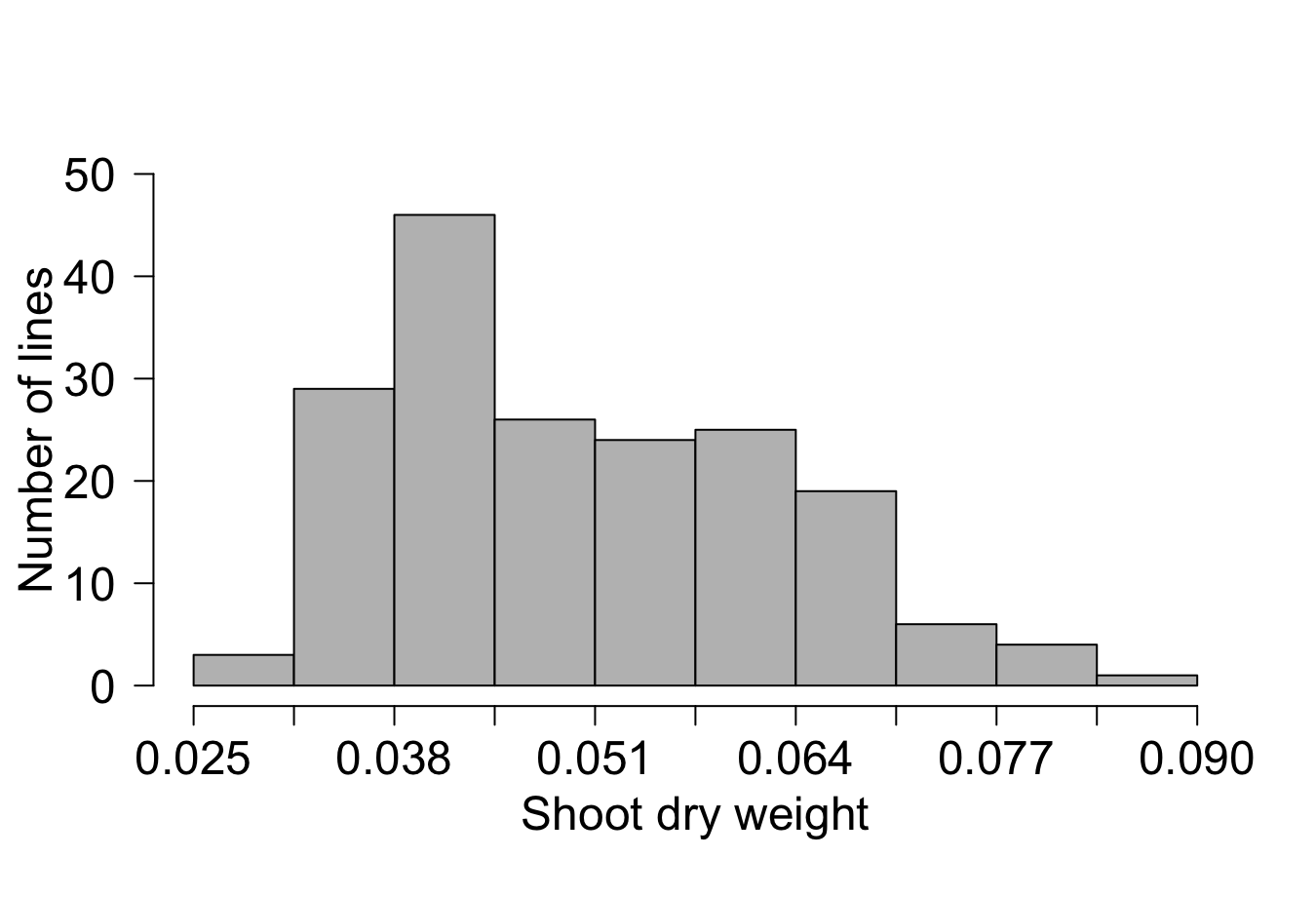

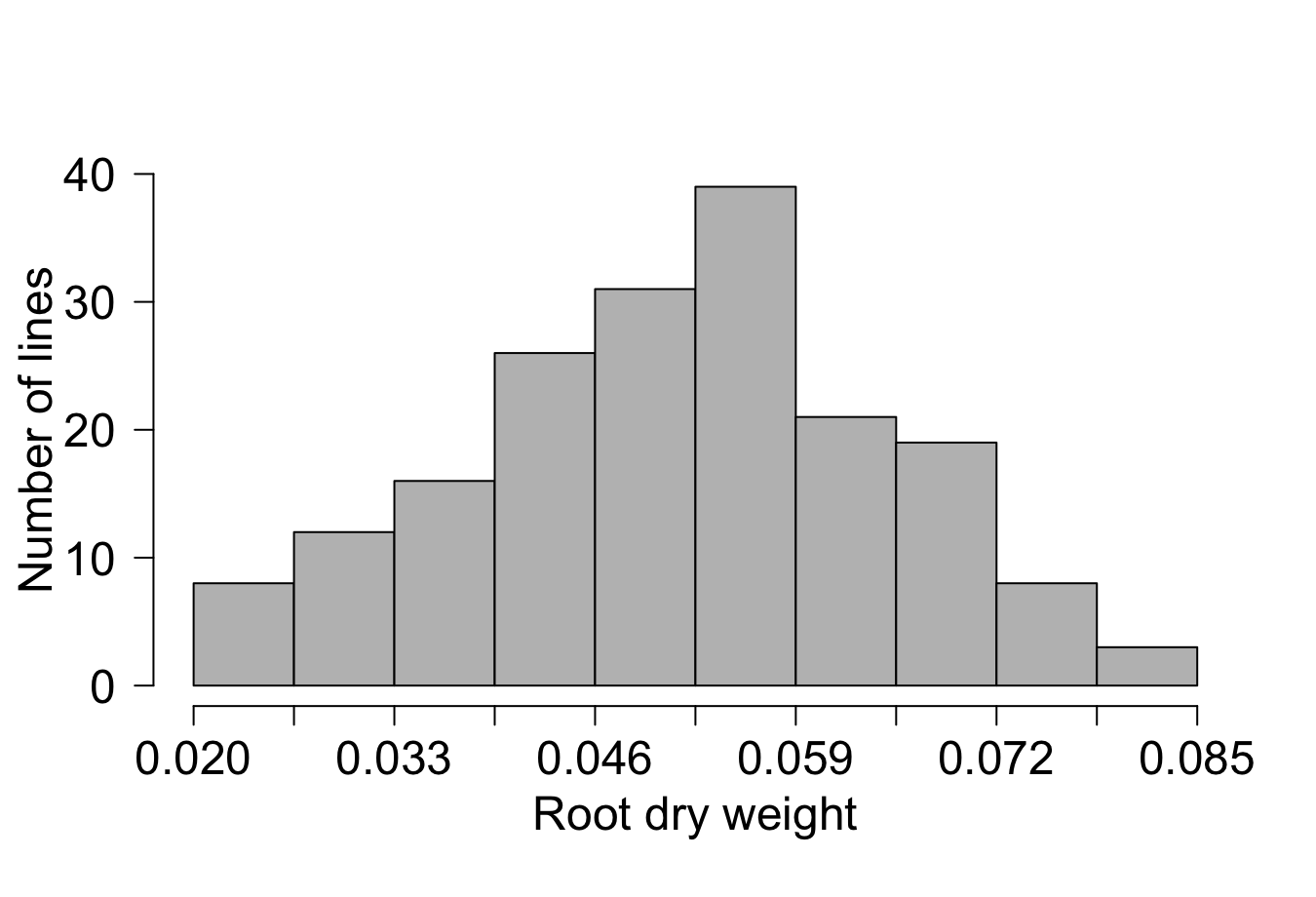


**Figure S11** Frequency distribution of phenotypic variation of shoot length, shoot/root dry weight in 183 *L. culinaris* landrace and cultivated accessions.

**
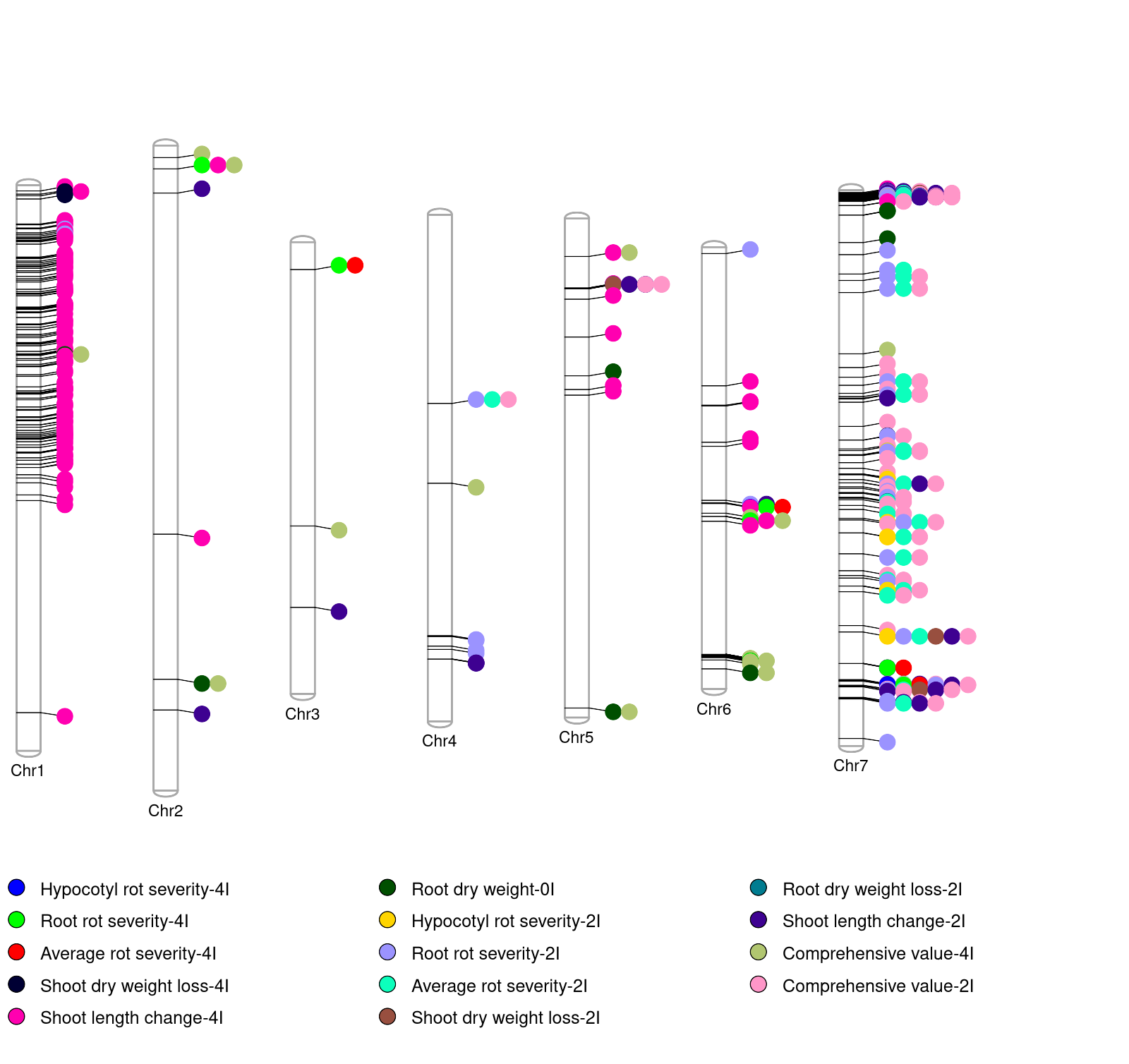
**

**Figure S12** The distribution of associated loci identified for Fusarium root rot (FRR). The colors indicate different FRR traits. 2I and 4I represent two different treatments, 2 × 10^6^ spores/mL and 4 × 10^6^ spores/mL respectively.


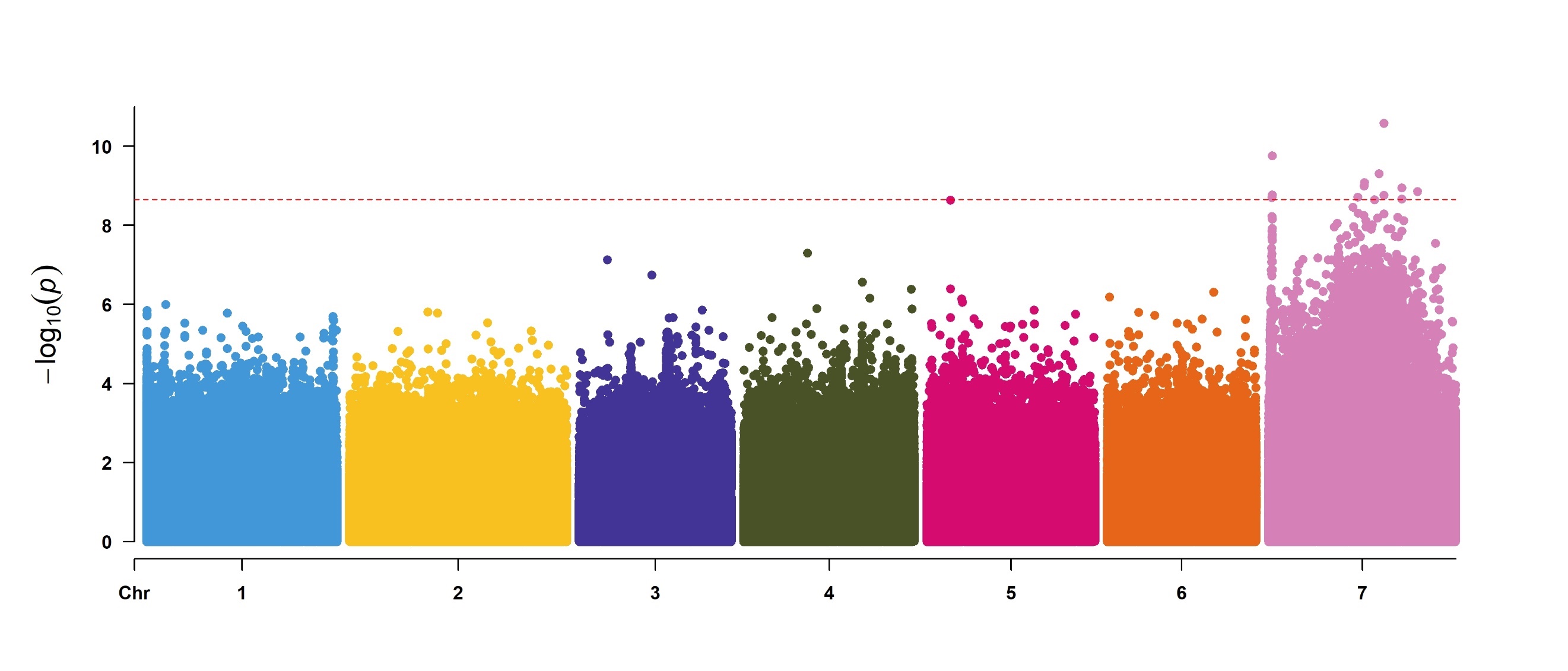

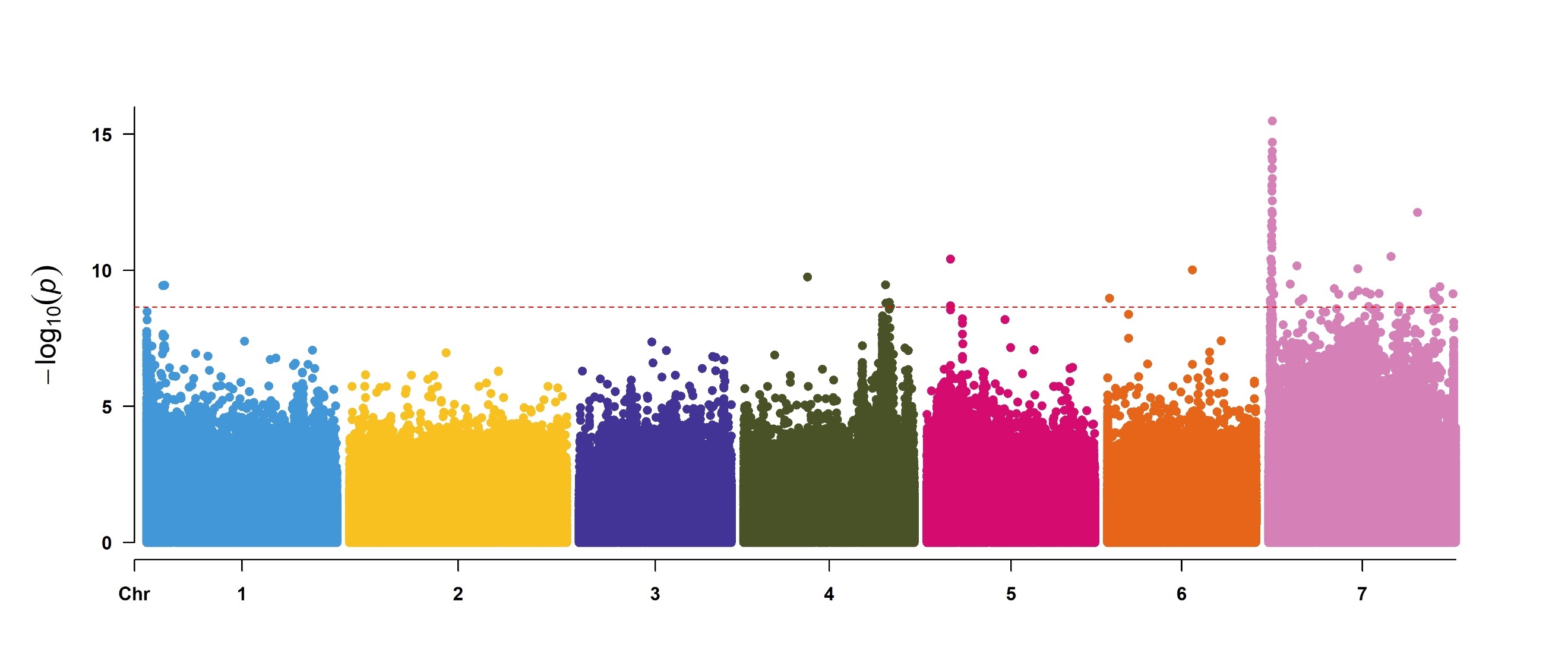

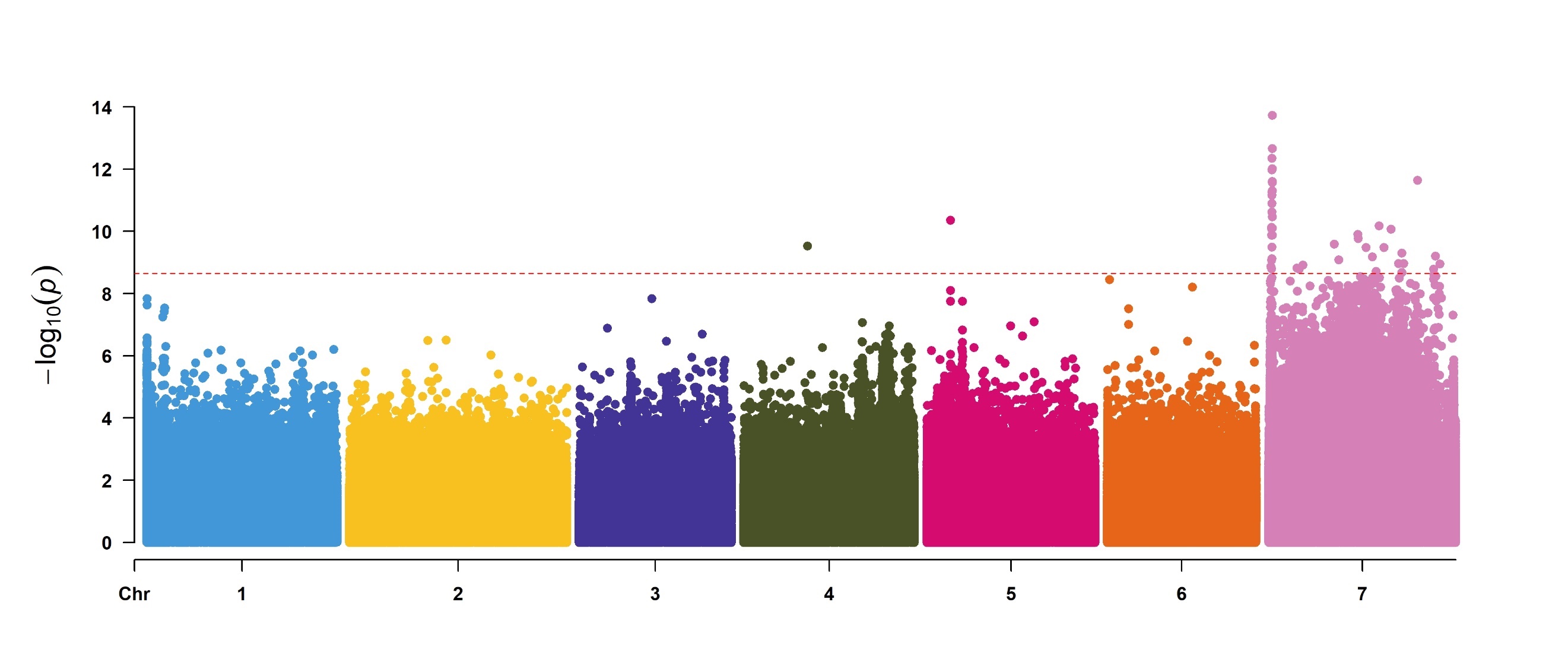


b

c

a

Root rot severity

Average rot severity

Hypocotyl rot severity


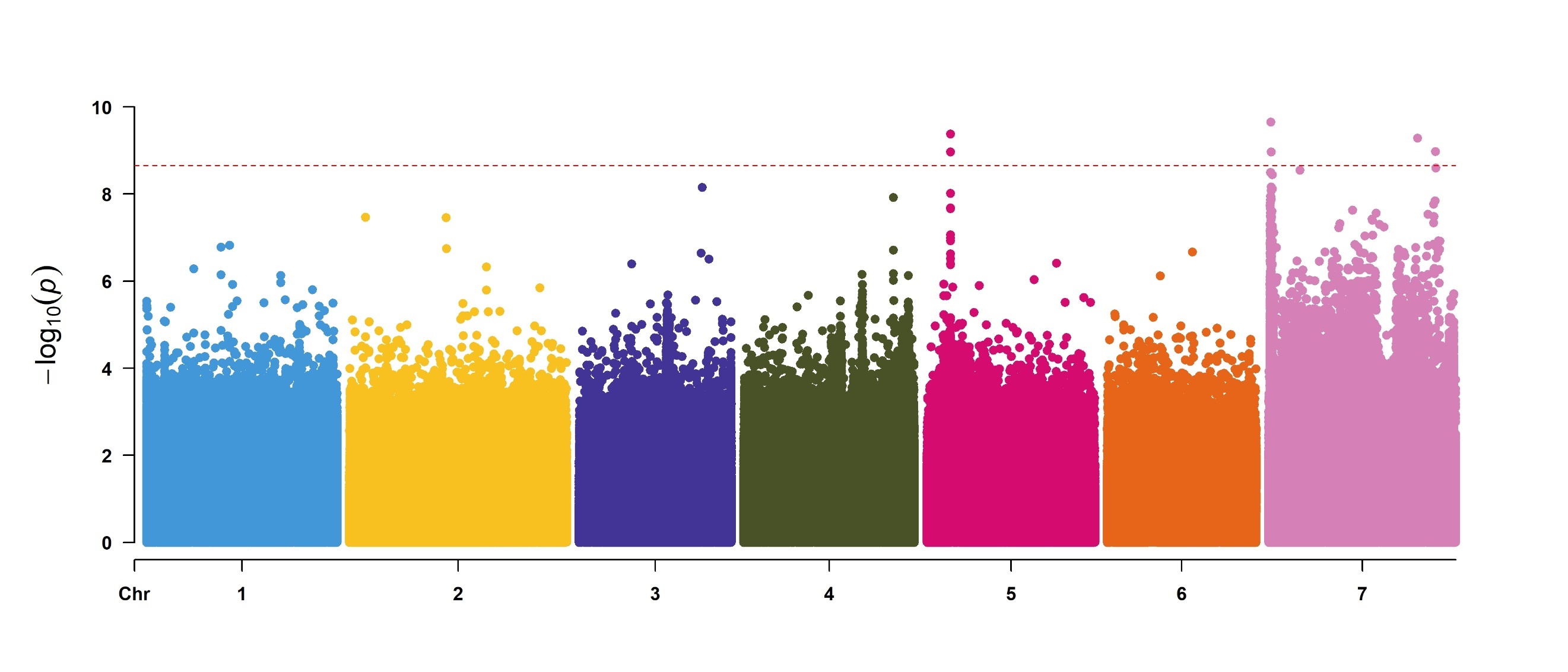

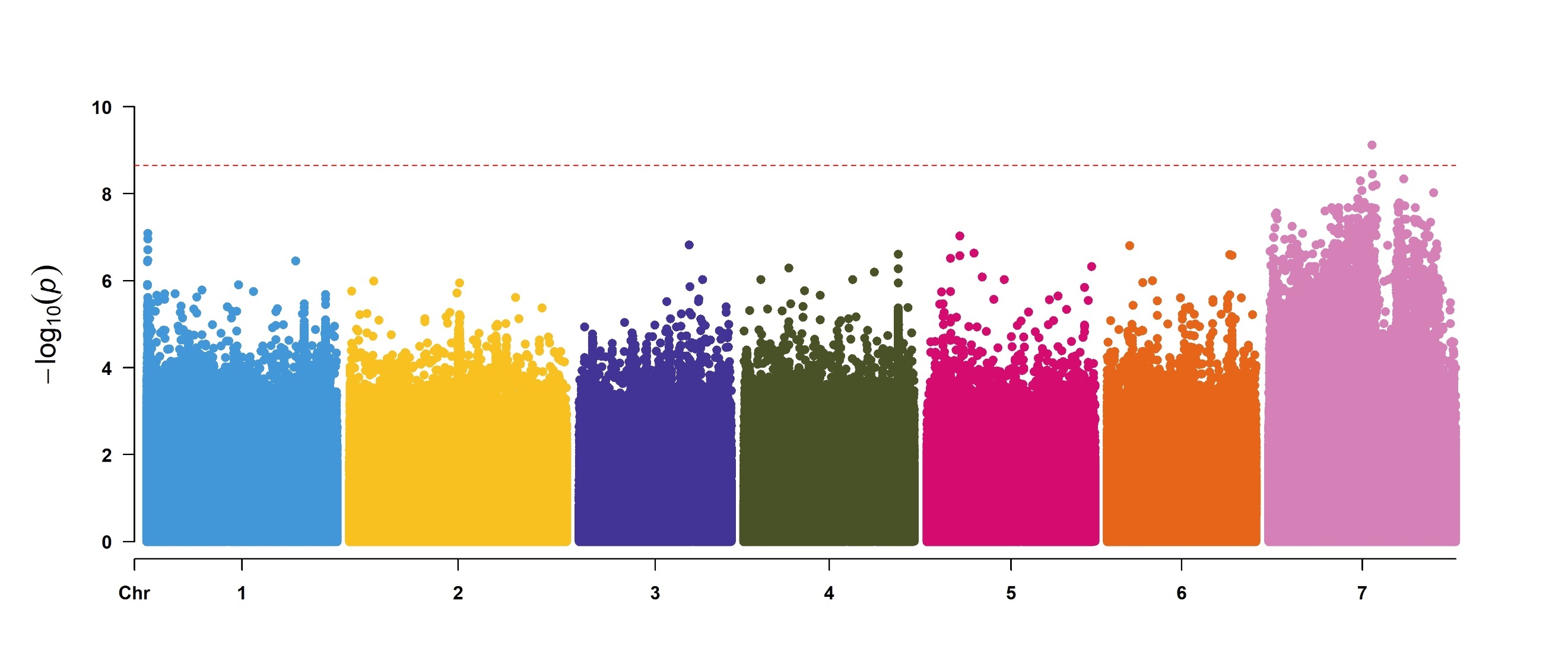


e

d

Root dry weight loss

Shoot dry weight loss


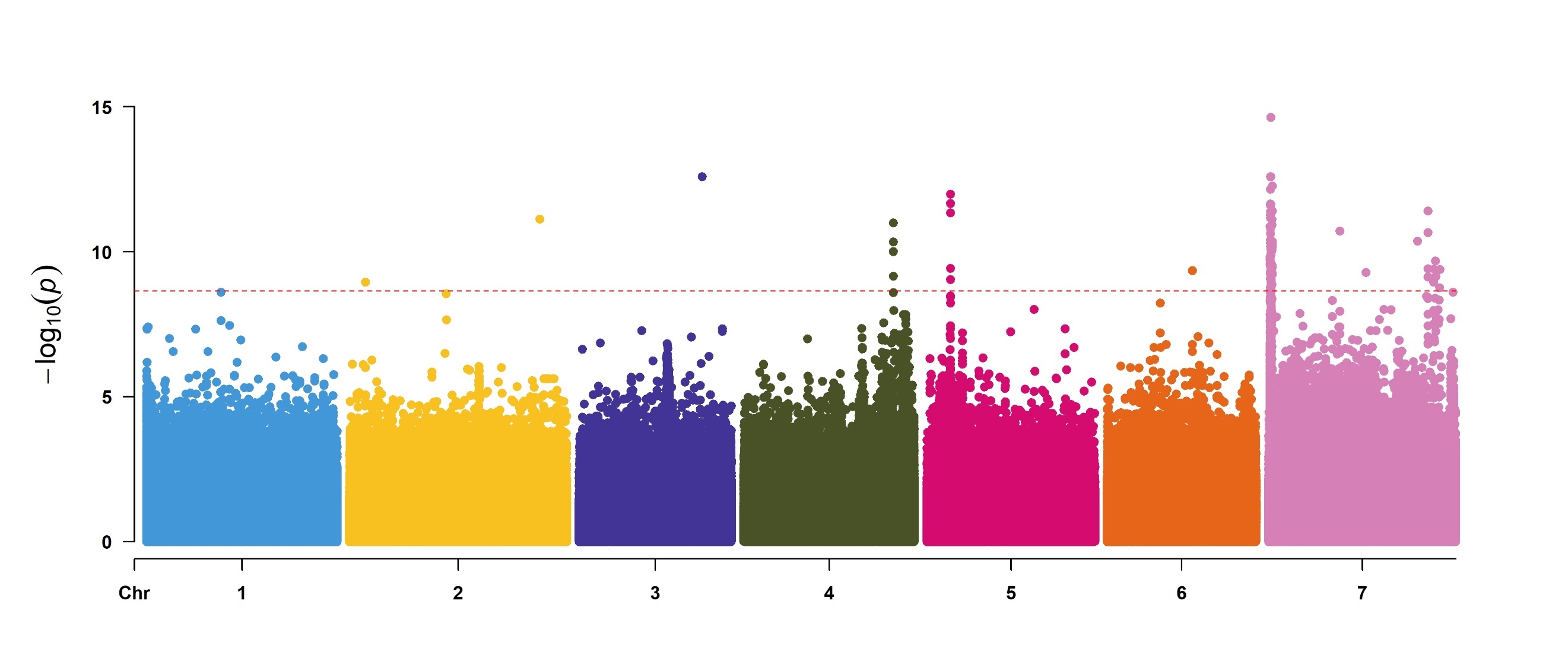

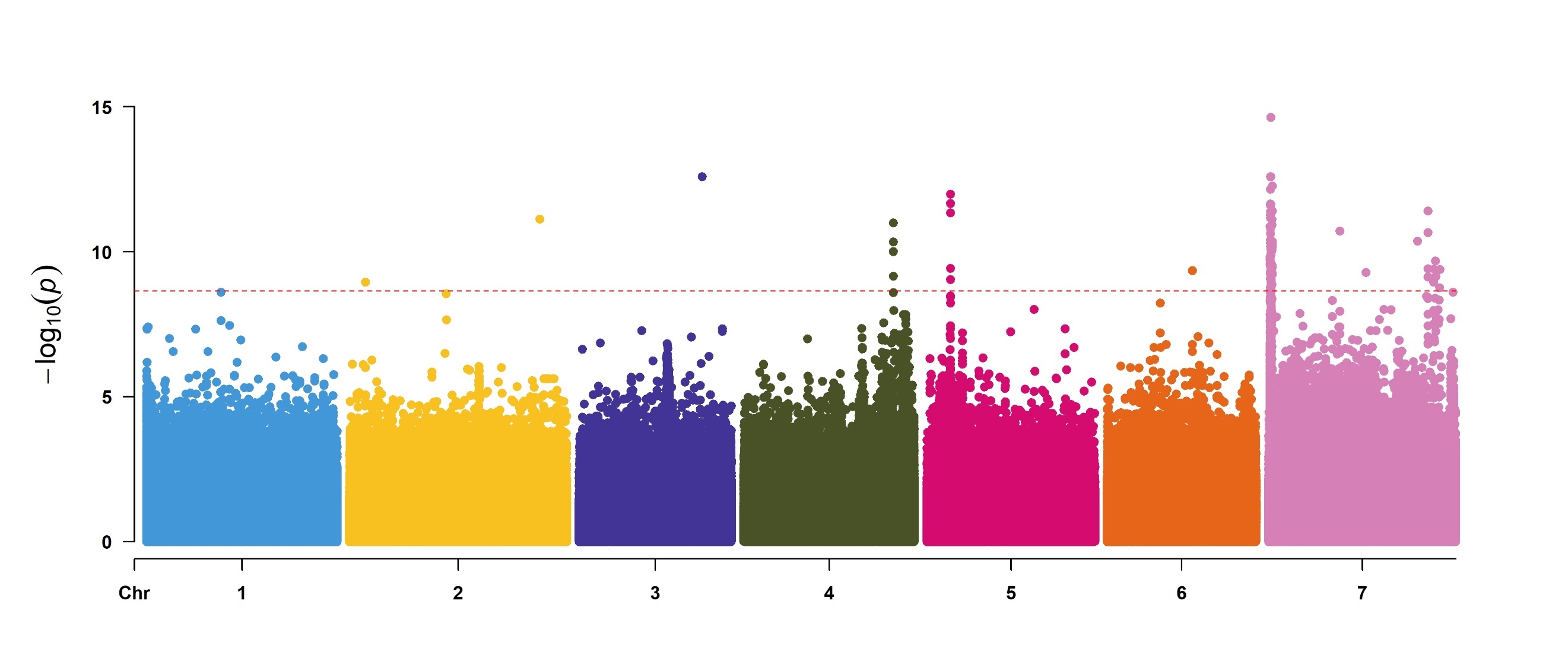

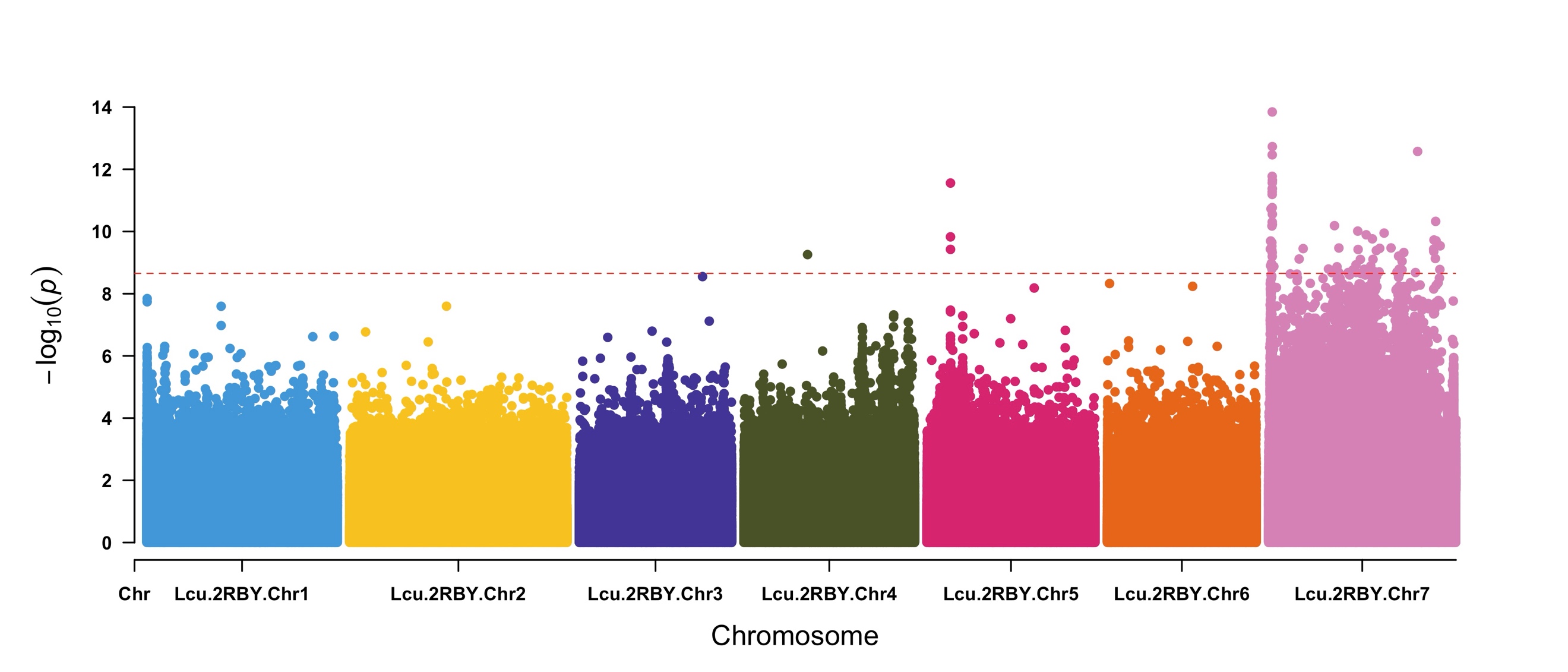


g

Comprehensive value

f

Shoot length change

**Figure S13** Genome-wide association study (GWAS) for Fusarium root rot (FRR) resistance under the 2I treatment. Manhattan plots for hypocotyl rot severity (a), root rot severity (b), average rot severity (c), shoot dry weight loss (d), root dry weight loss (e), shoot length change (f), comprehensive value (g). Red horizontal dashed lines represent the significant thresholds used for GWAS

**
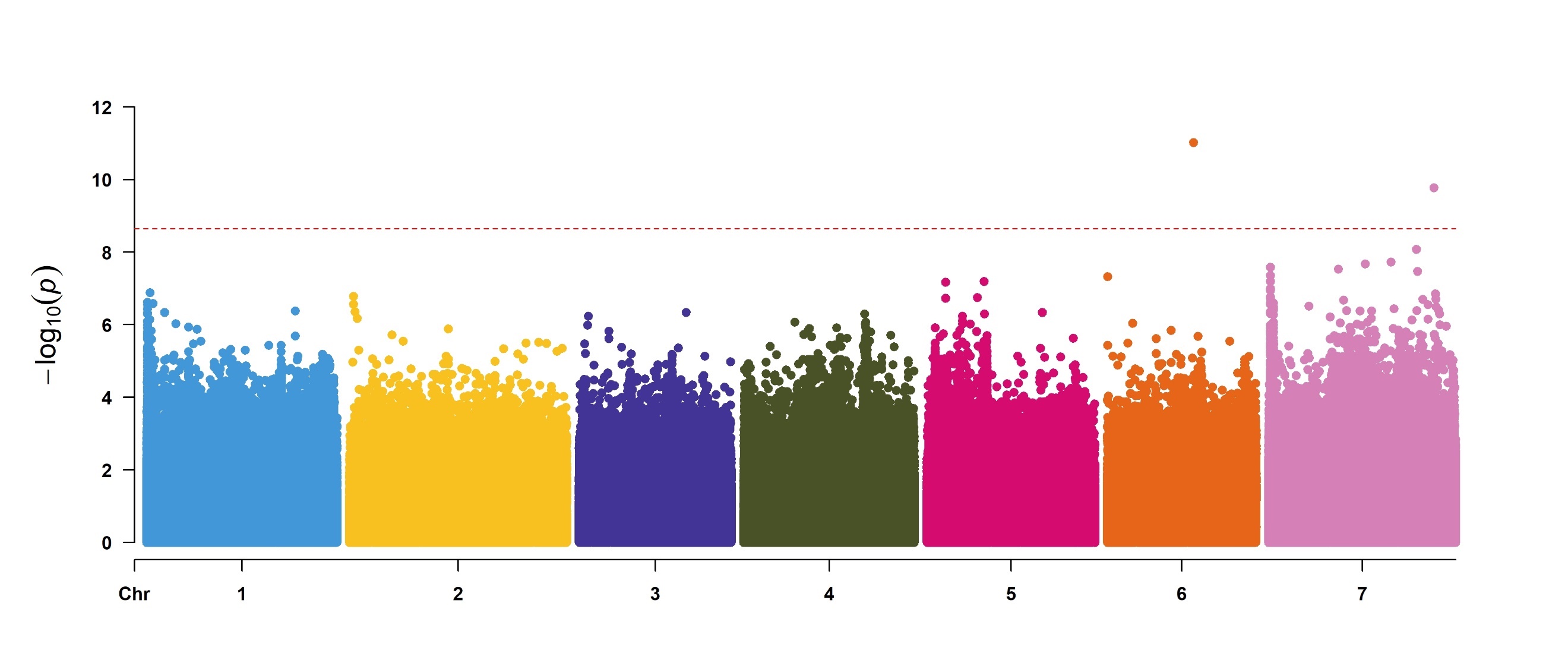
**

a

Hypocotyl rot severity

**
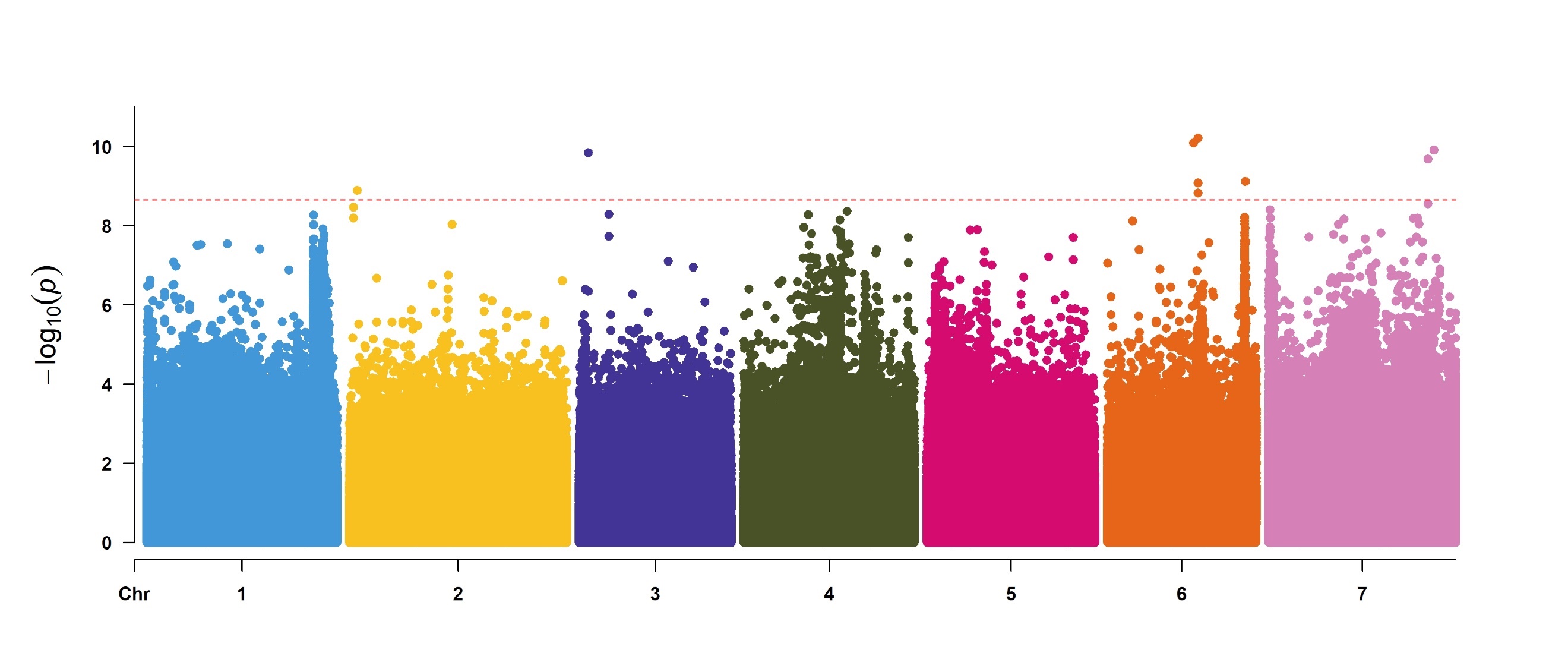
**

b

Root rot severity

**
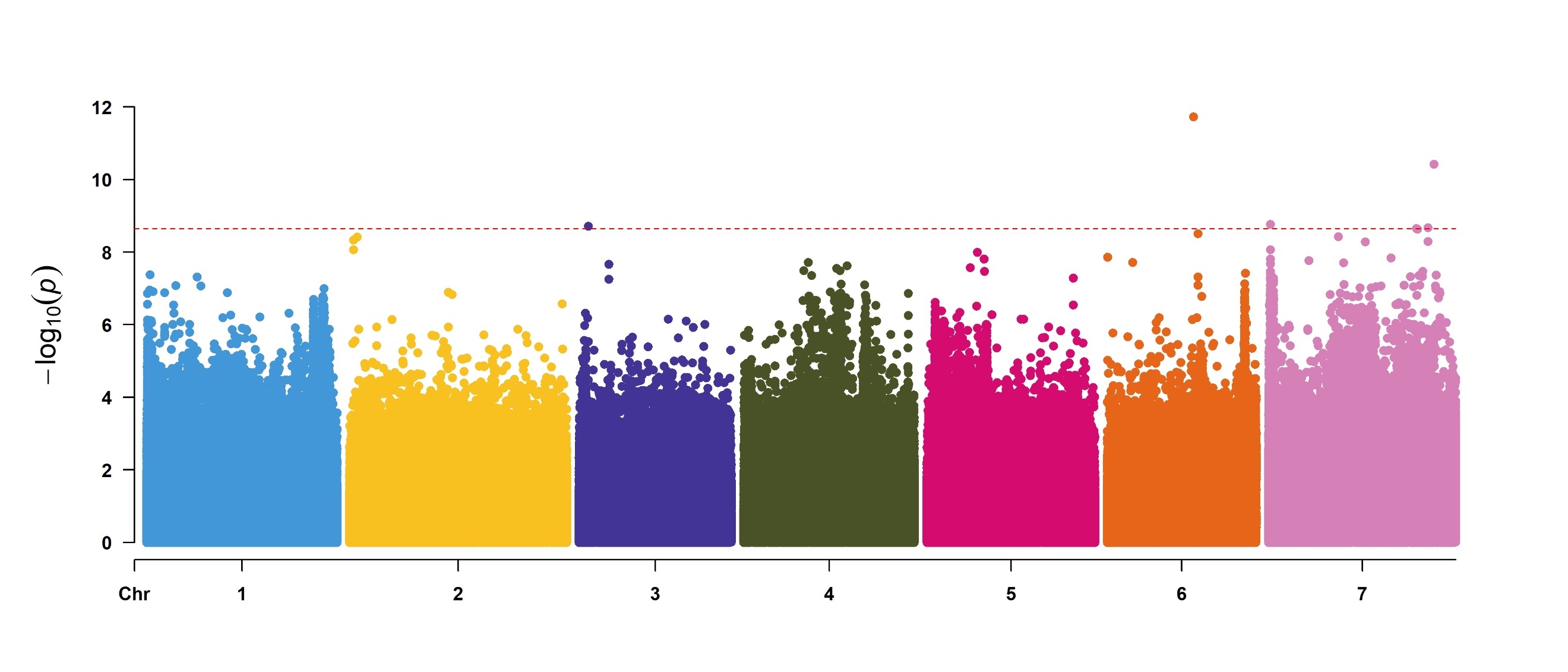
**

c

Average rot severity

**
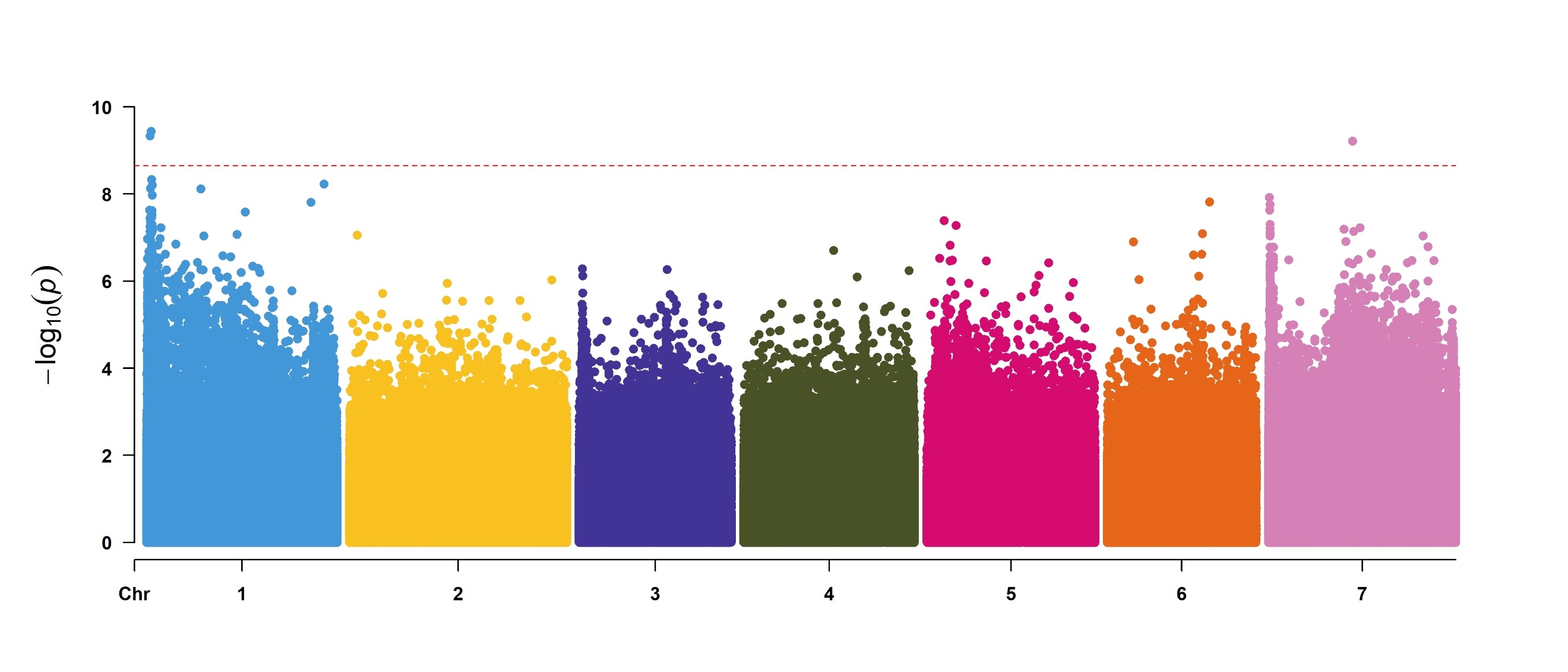

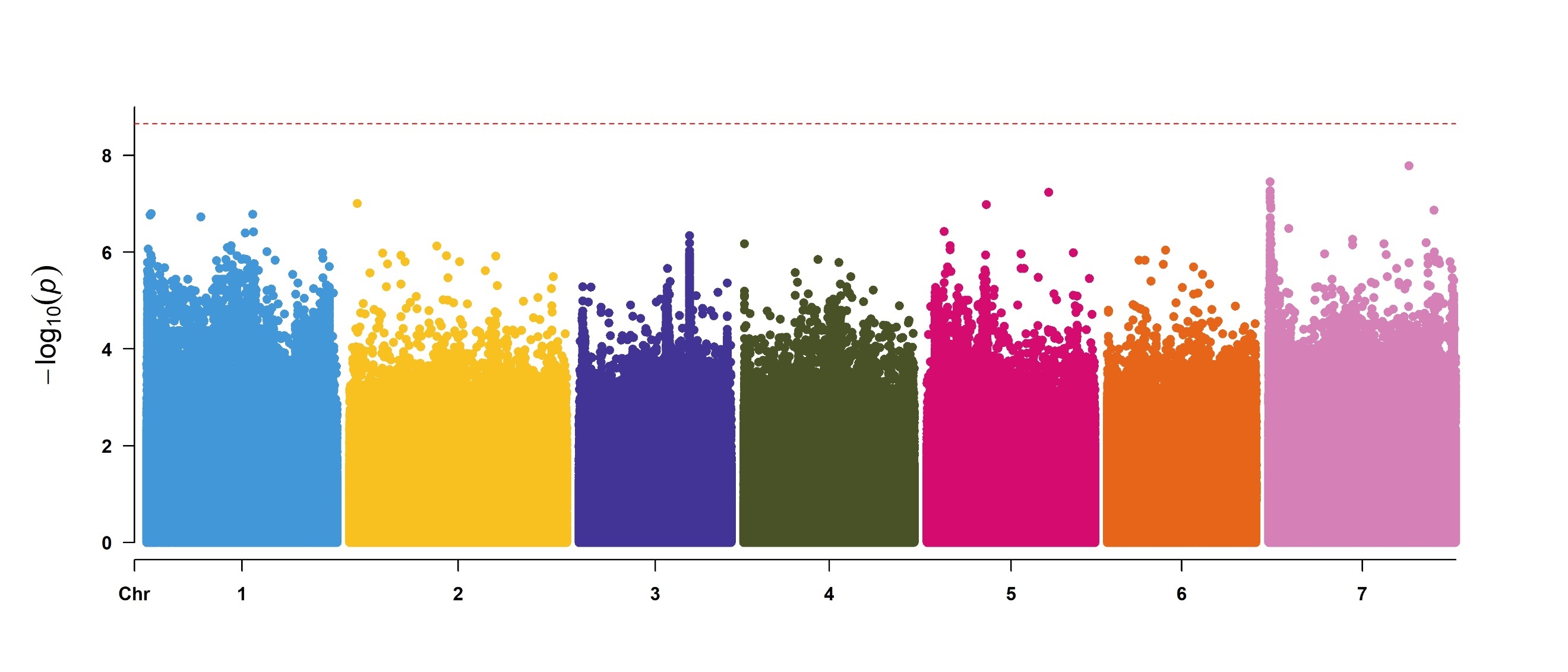
**

e

d

Root dry weight loss

Shoot dry weight loss


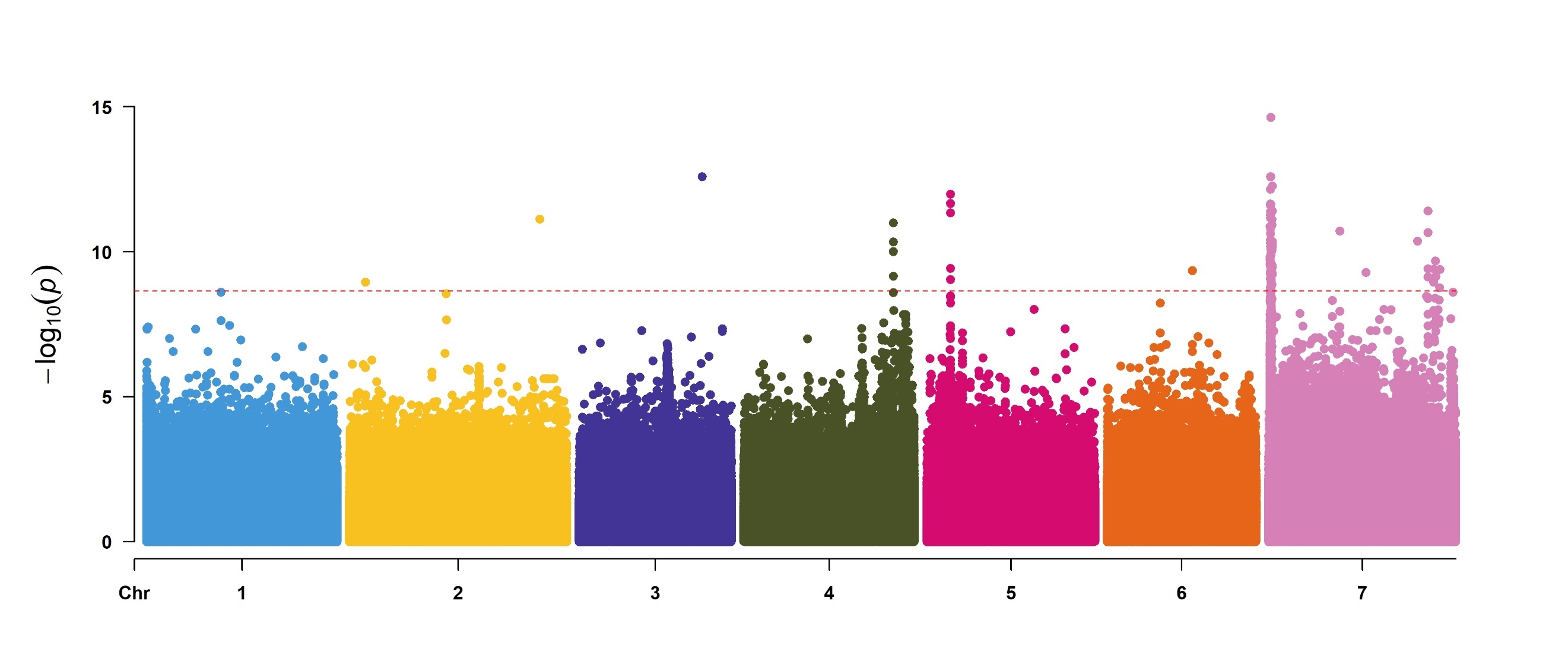
**
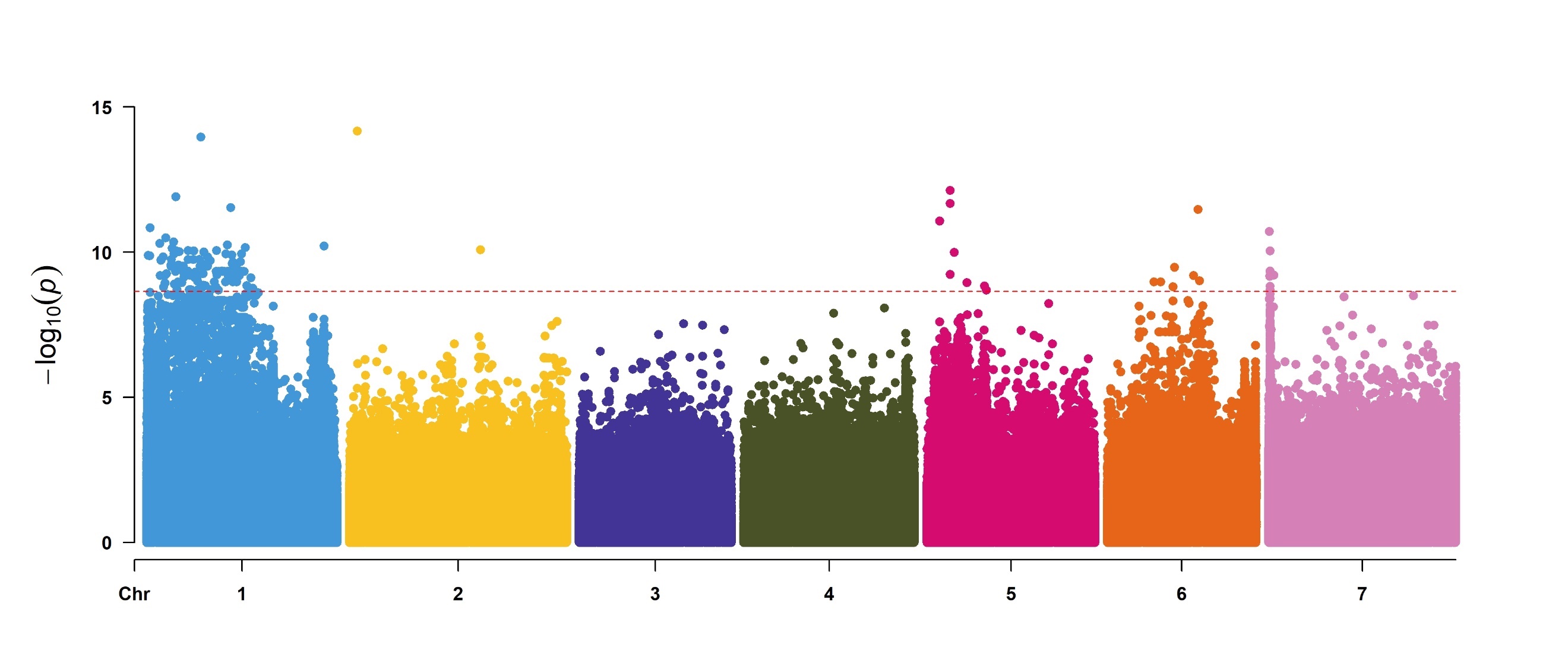

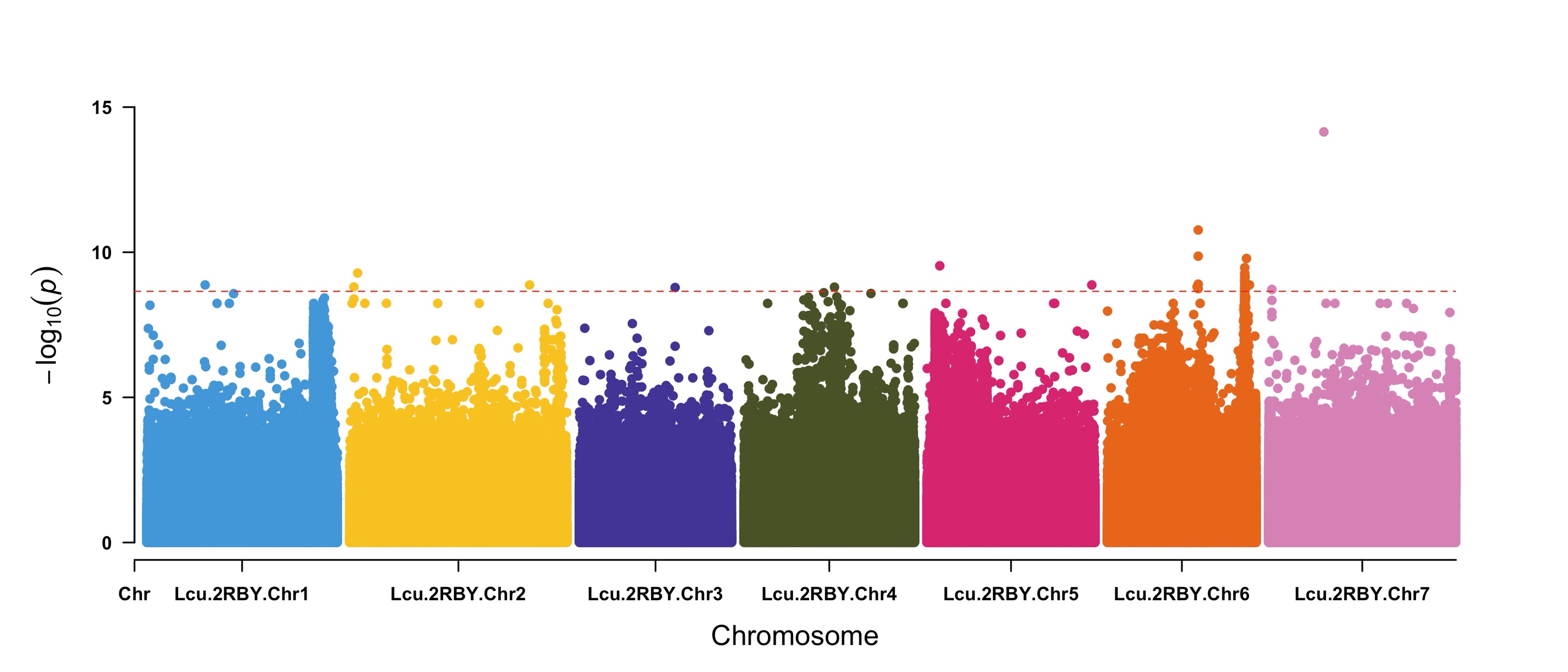
**

g

Comprehensive value

f

Shoot length change

**Figure S14** Genome-wide association study (GWAS) for Fusarium root rot (FRR) resistance under the 4I treatment. Manhattan plots for hypocotyl rot severity (a), root rot severity (b), average rot severity (c), shoot dry weight loss (d), root dry weight loss (e), shoot length change (f), comprehensive value (g). Red horizontal dashed lines represent the significant thresholds used for GWAS

**
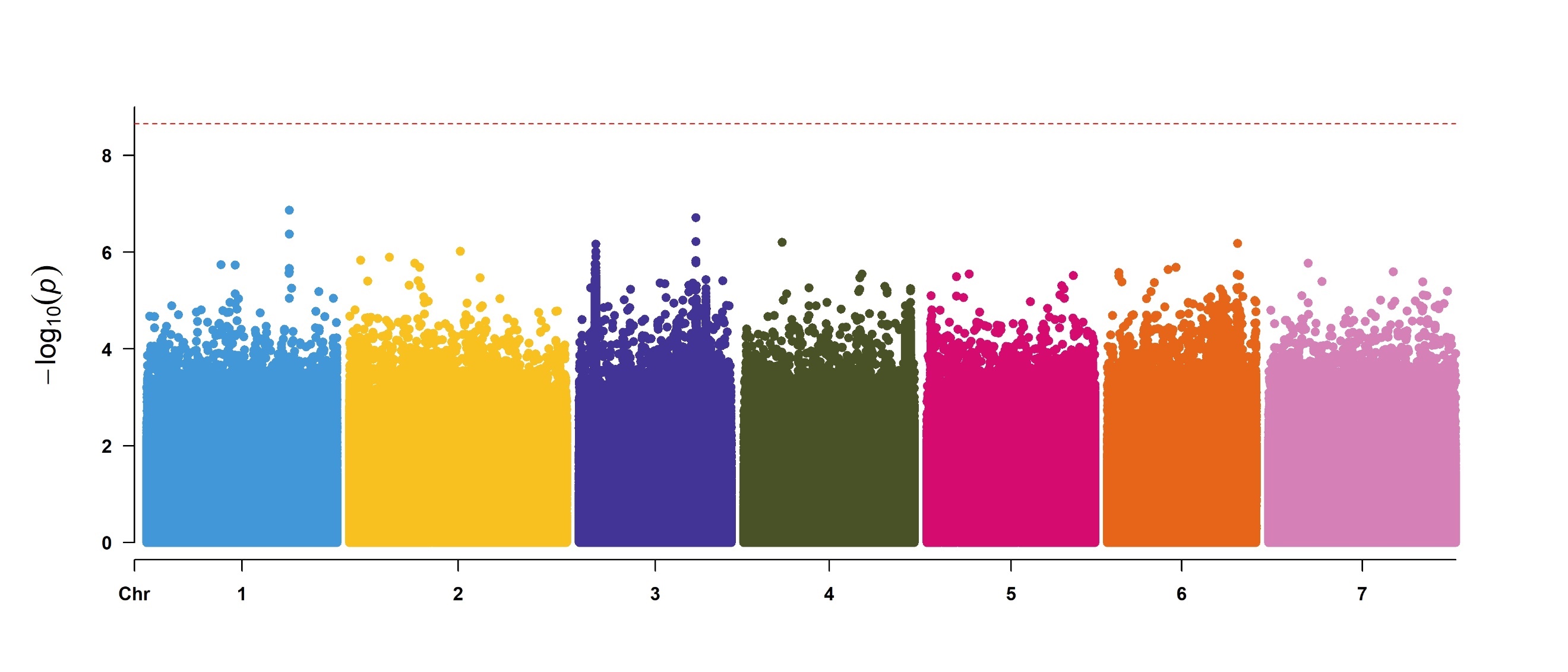

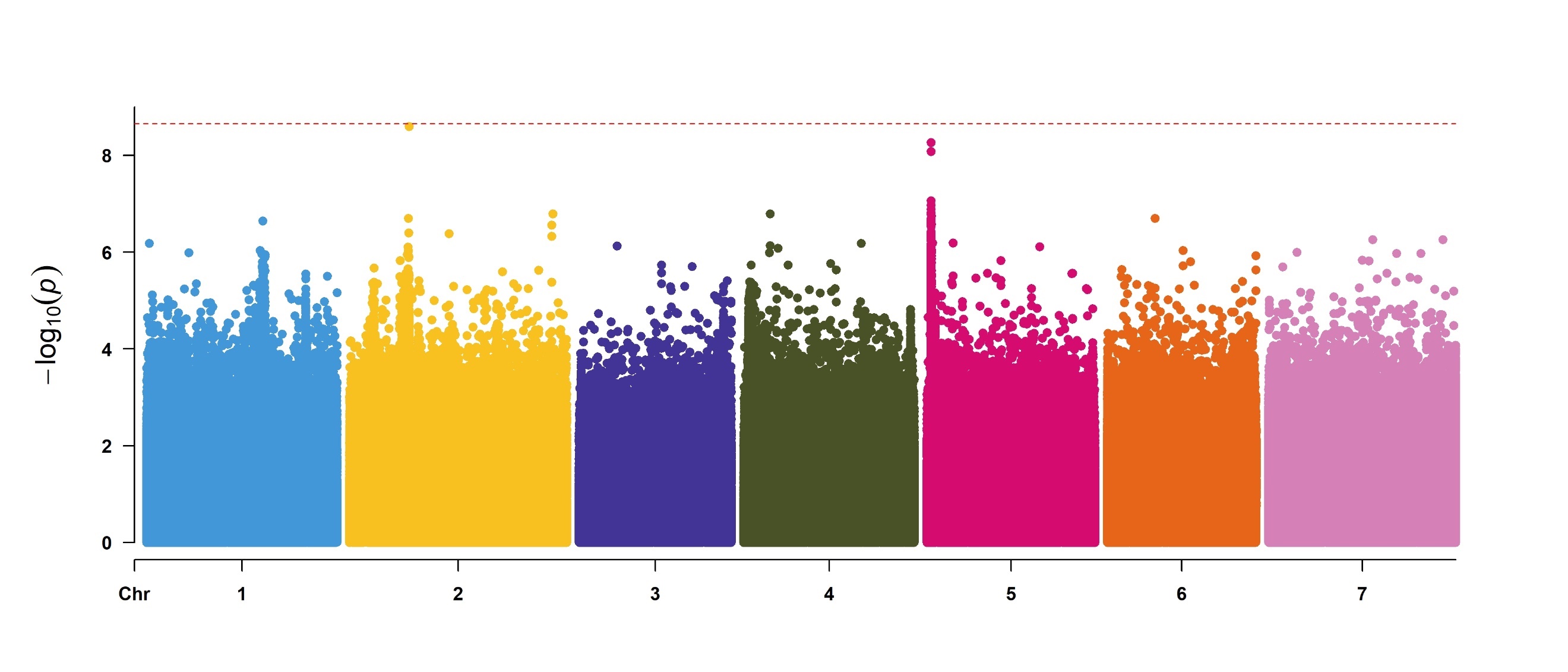
**

b

a

Shoot dry weight

Shoot length

**
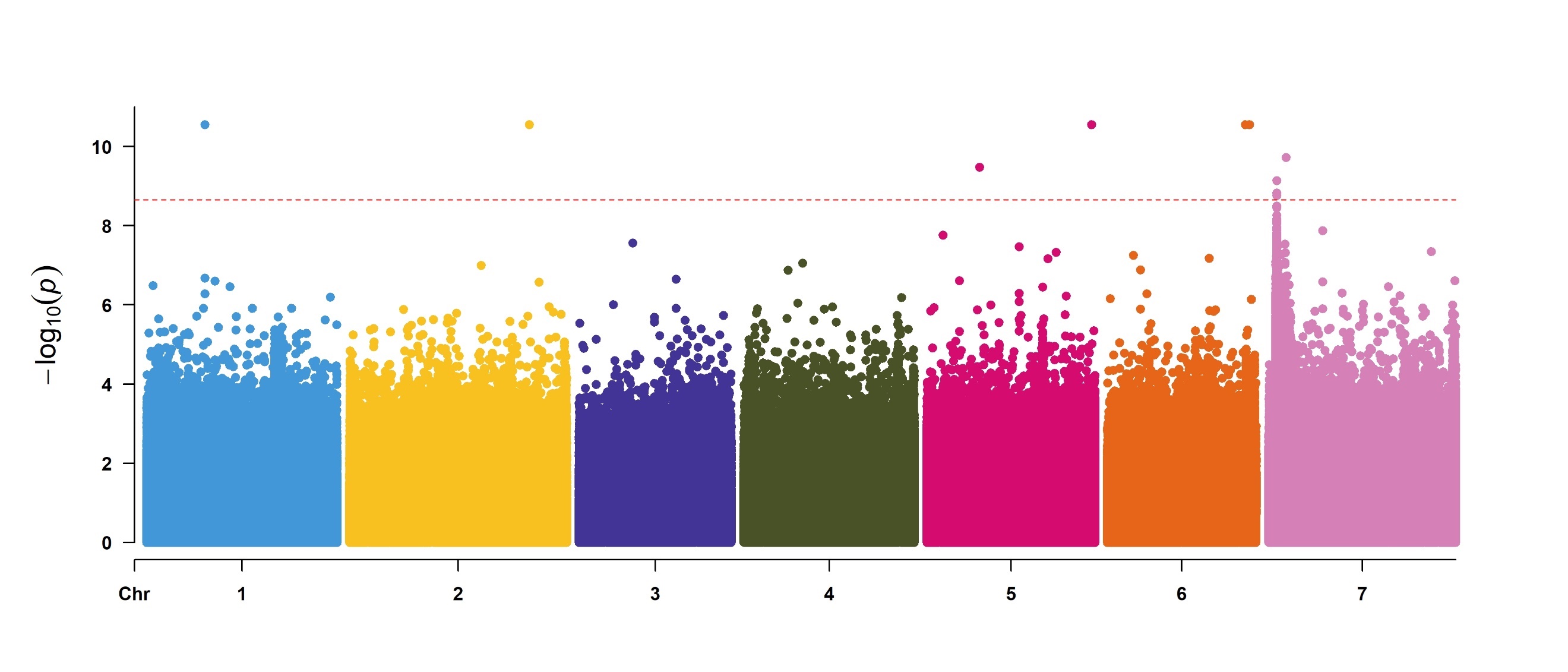
**

c

Root dry weight

**Figure S15** Genome-wide association study (GWAS) for shoot length and dry matters. Manhattan plots for shoot length (a), root dry weight (b), shoot dry weight (c). Red horizontal dashed lines represent the significant thresholds used for GWAS.


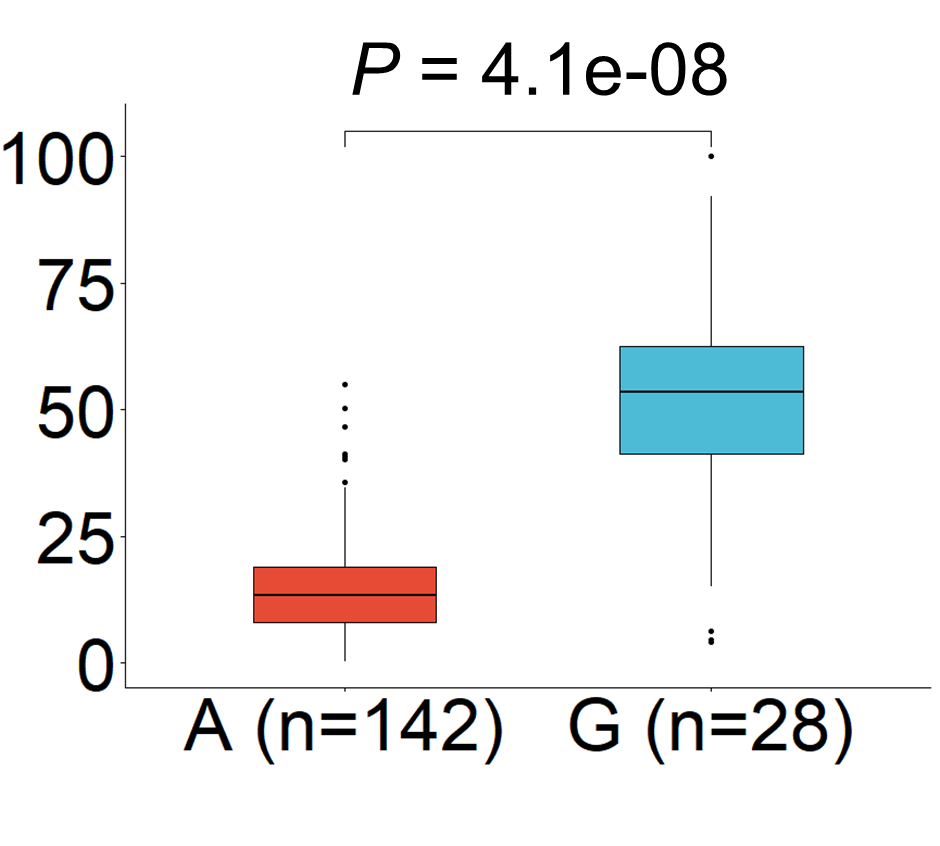

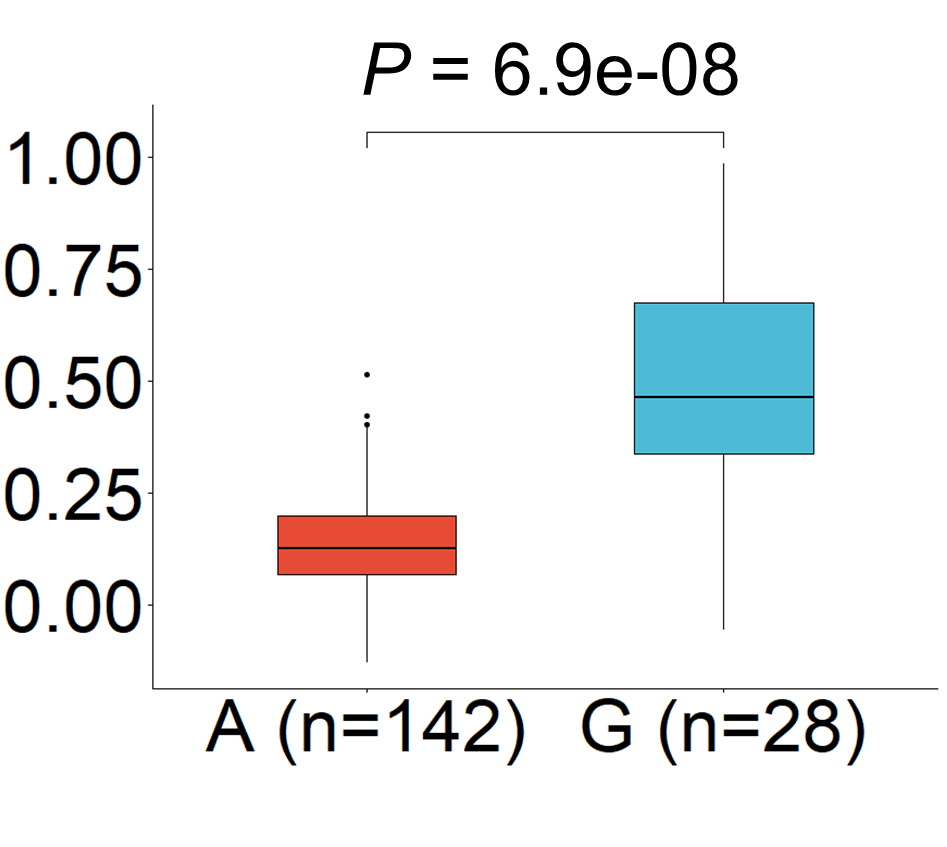
 **
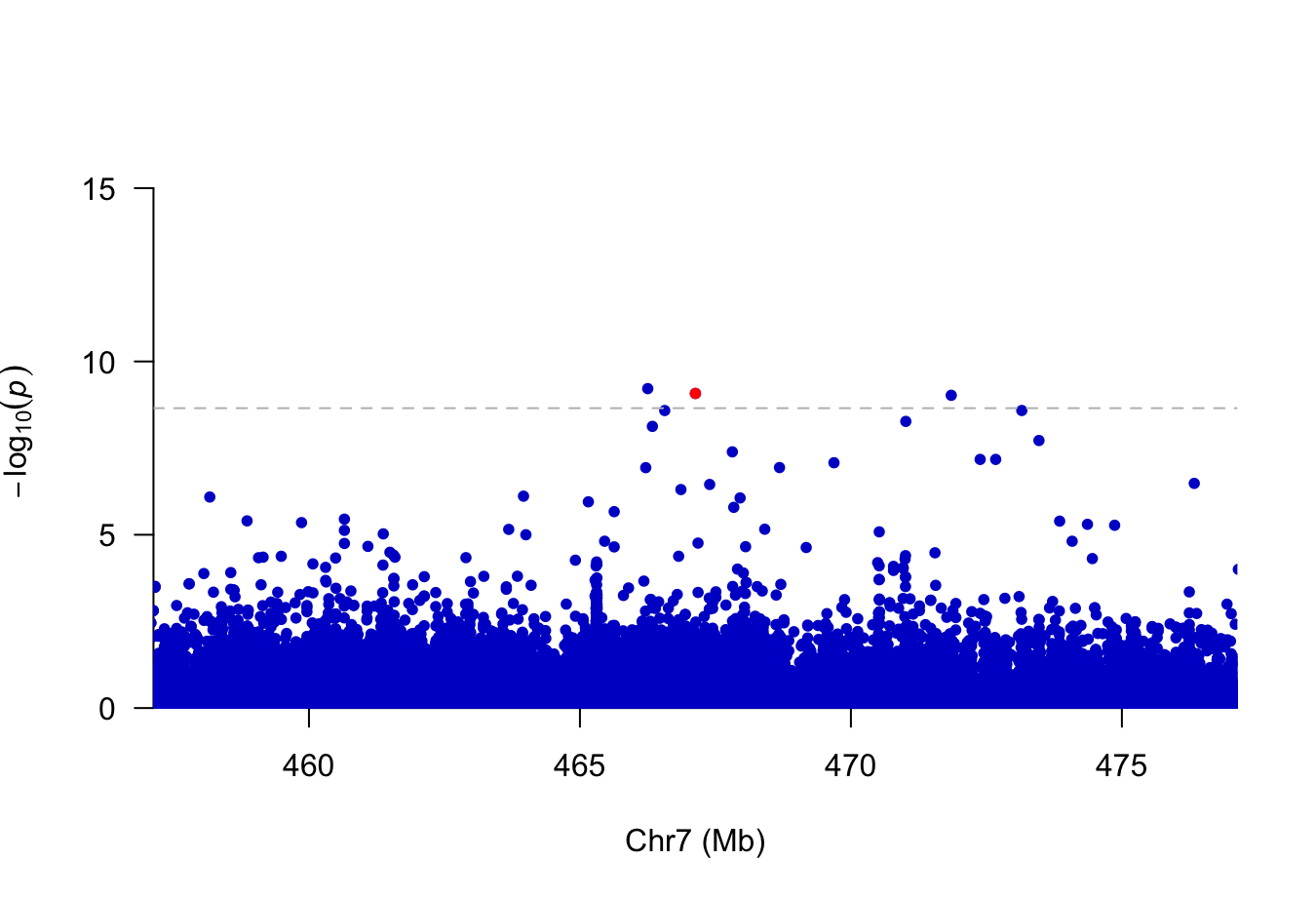
**
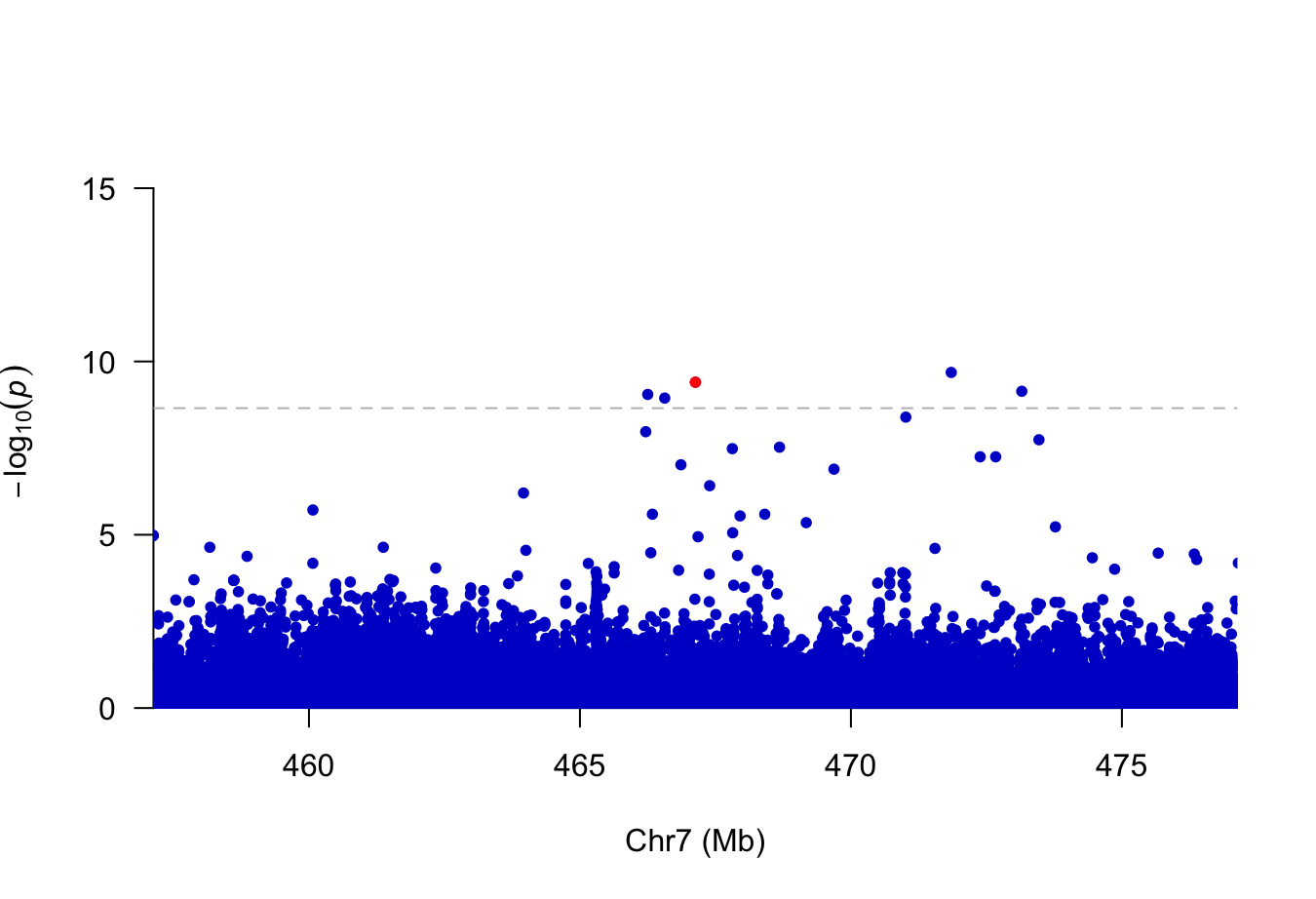

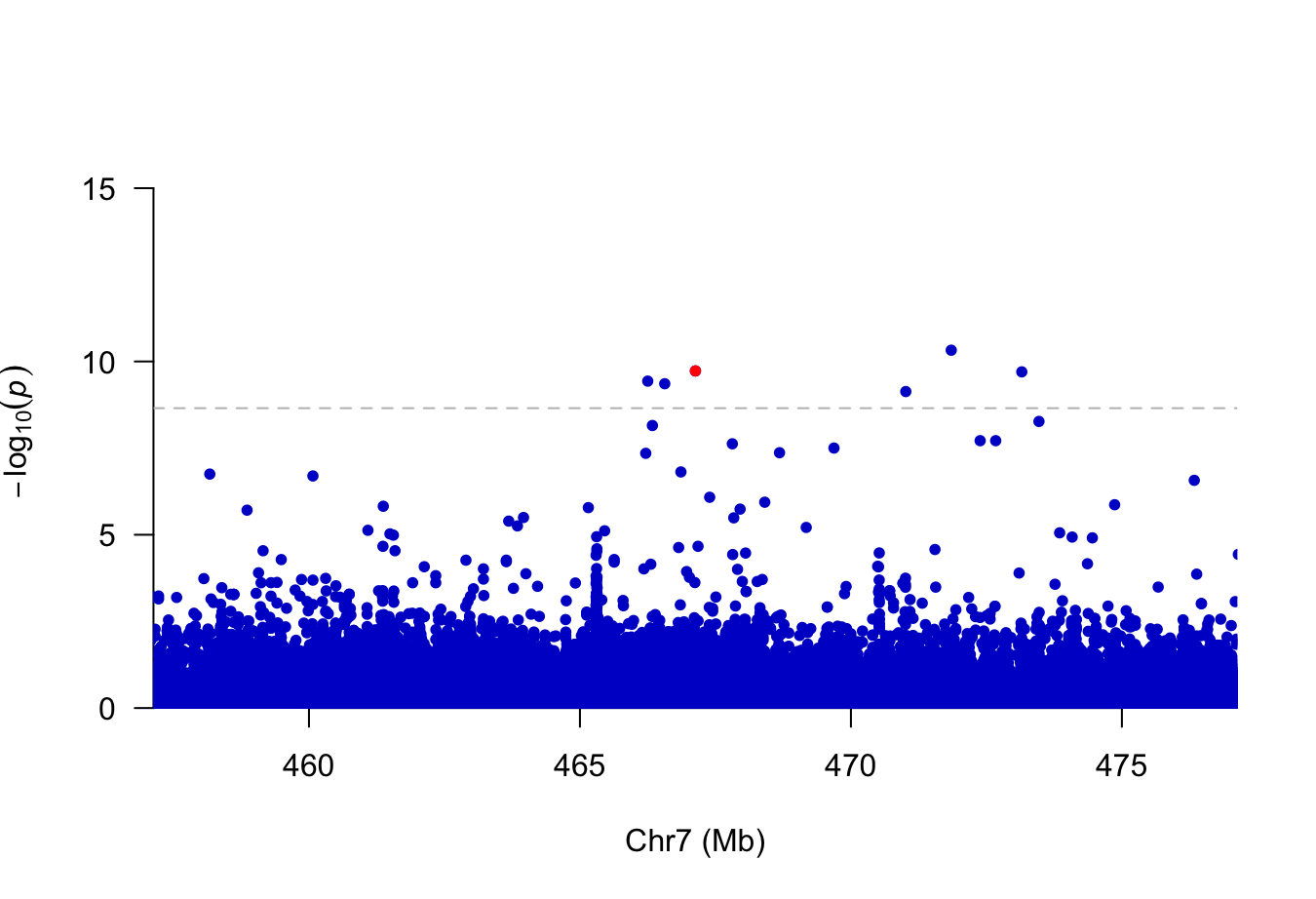


b

f

c

Comprehensive value (2I)

Shoot length change (2I)

Root rot severity (2I)

e

d

a

**
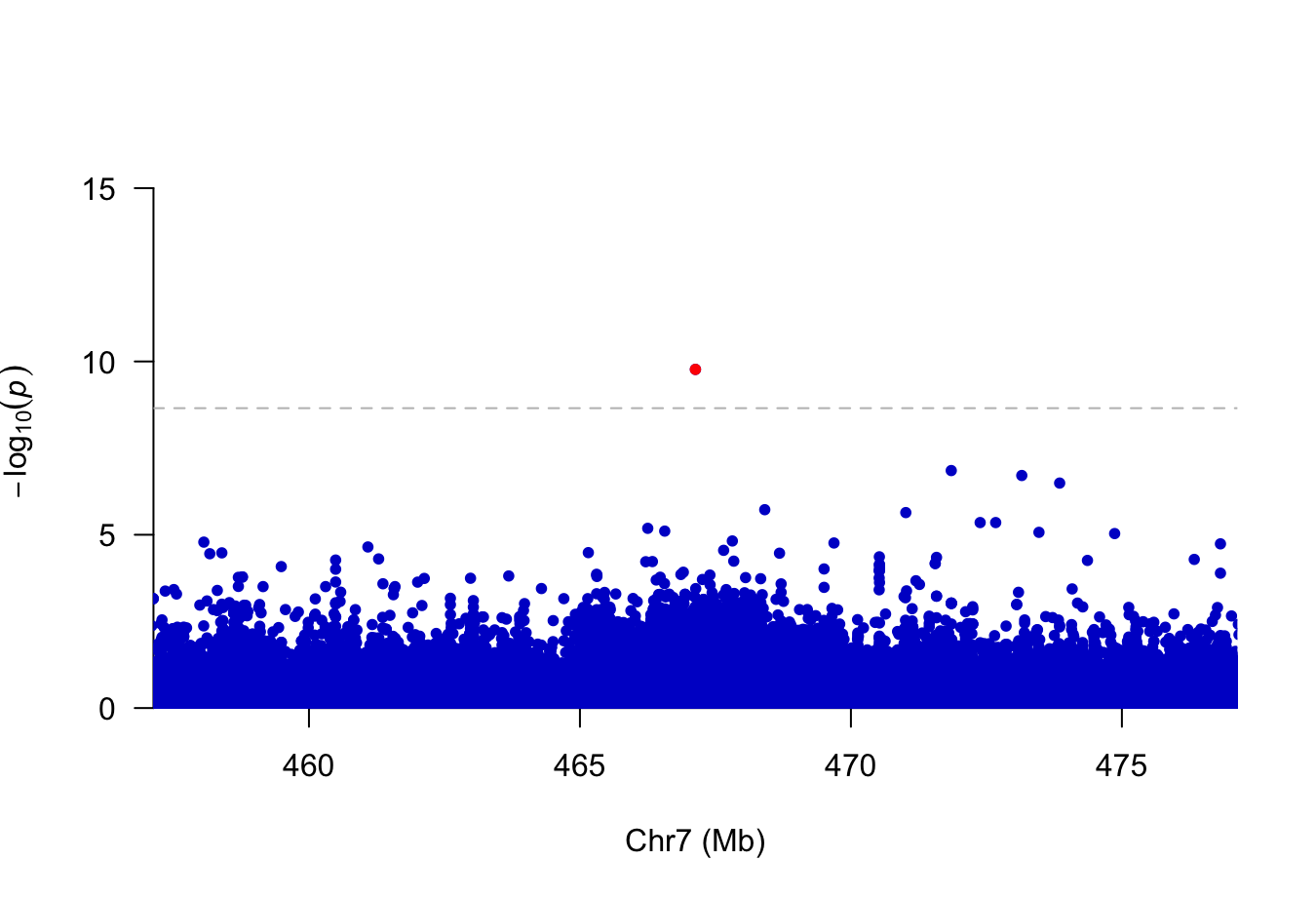
** **
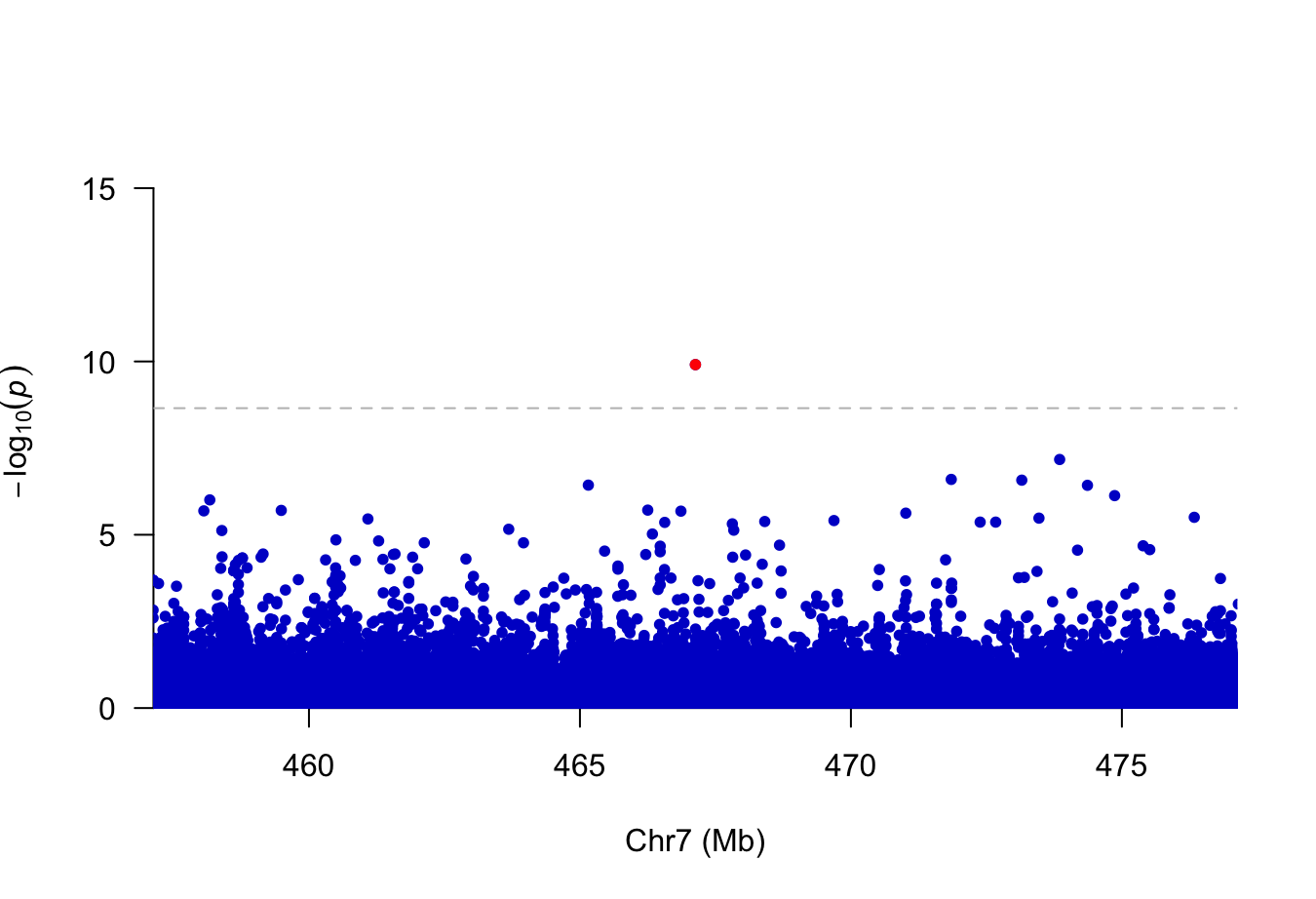
**
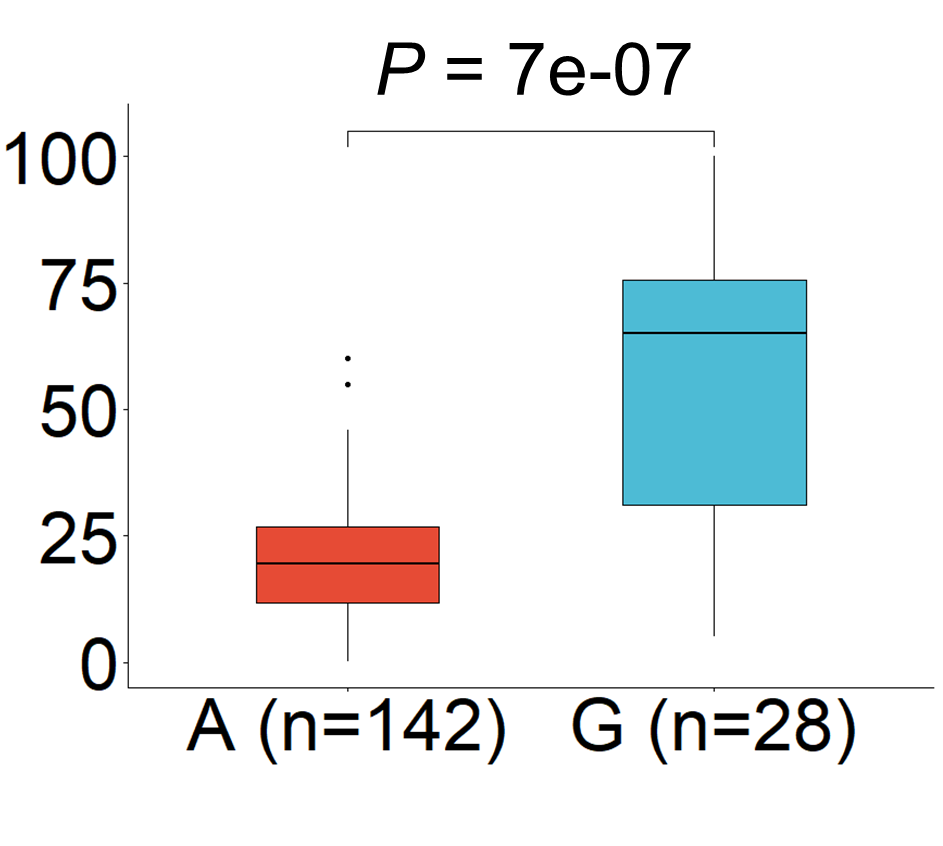

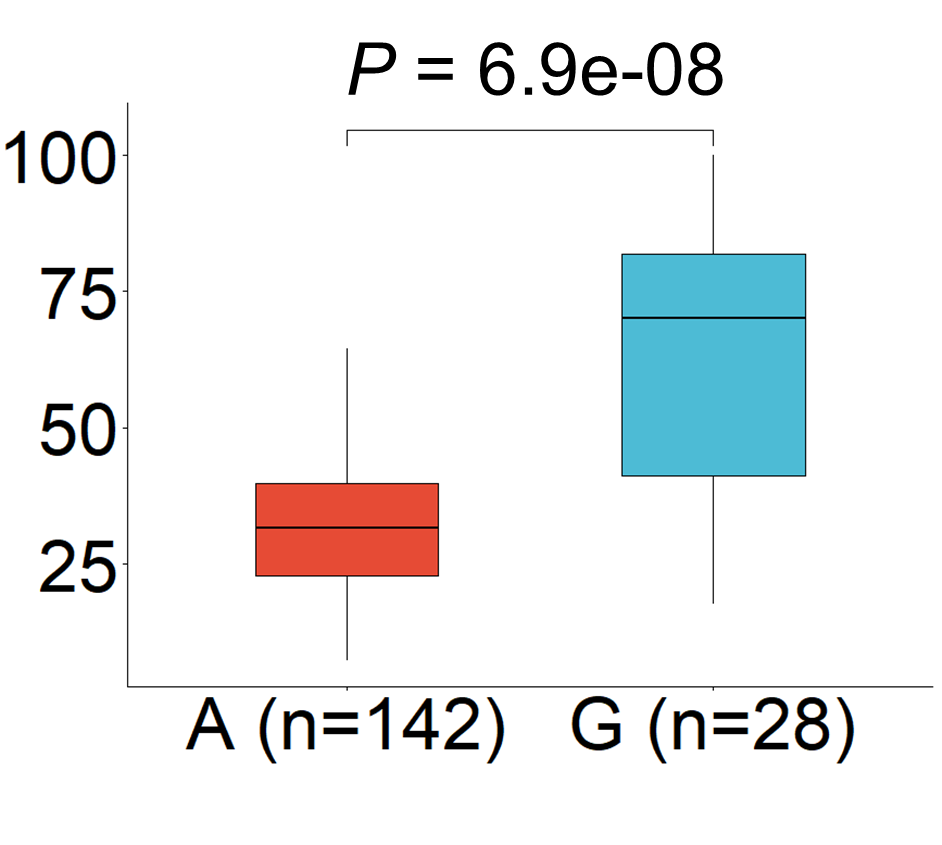

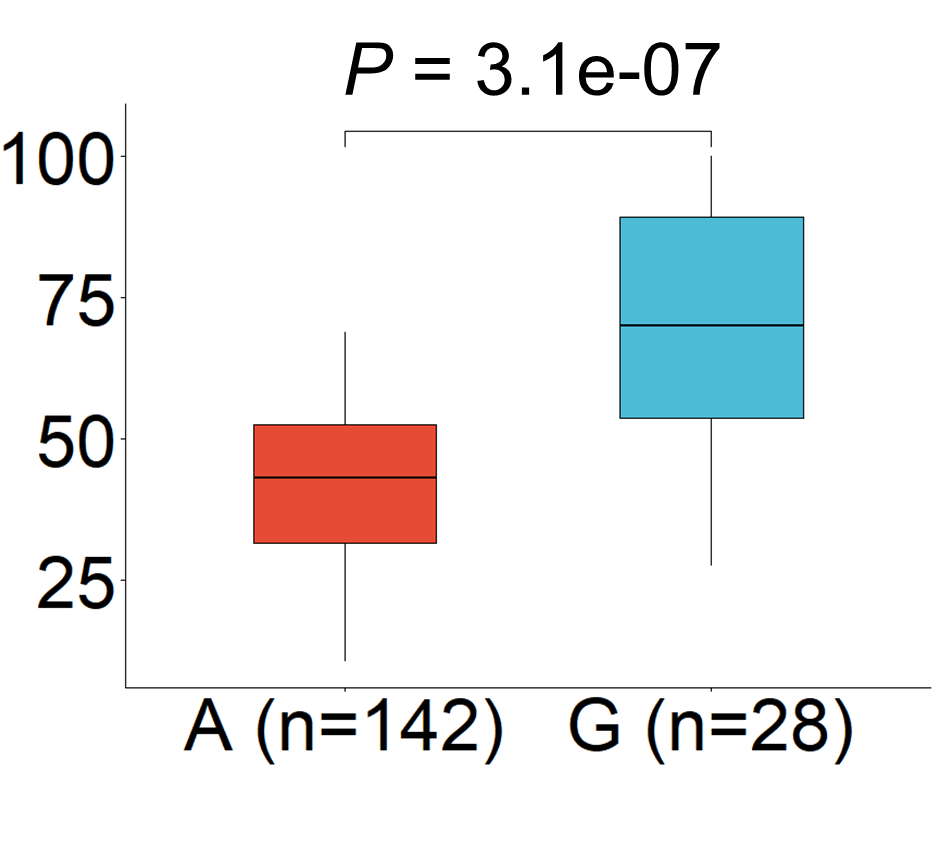

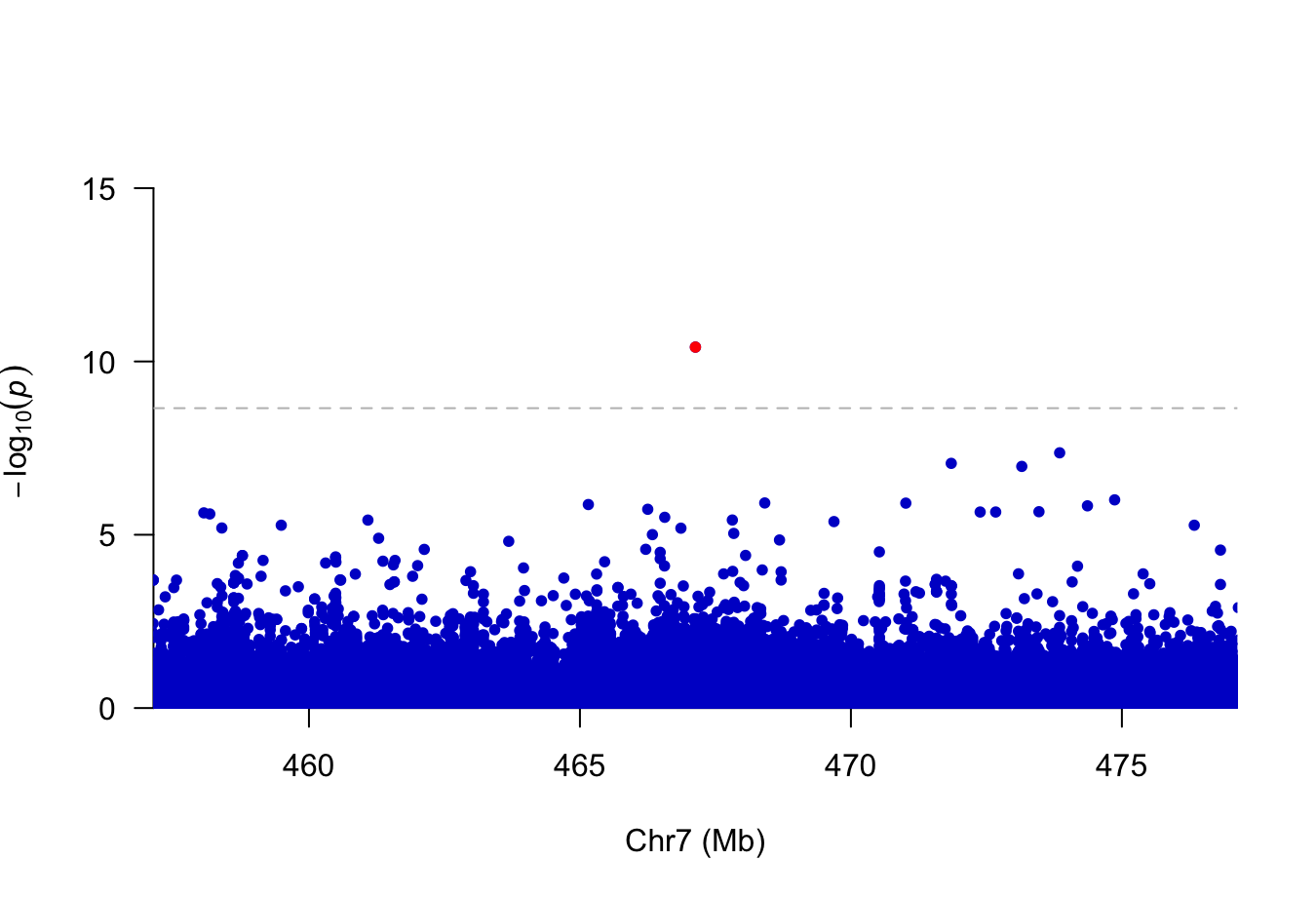


Average rot severity (4I)

Root rot severity (4I)

Hypocotyl rot severity (4I)

**Figure S16** Local Manhattan plots of genome-wide association on chromosome 7 (Lcu.2RBY.Chr7: 467,130,974) discovered under both treatments (2I and 4I) for Fusarium root rot (FRR). The grey horizontal dashed line represents the Bonferroni-corrected significance thresholds of GWAS (α = 0.05). The significant peak is indicated by the black arrow. The bar plots show FRR resistance levels of the lentil accessions carrying each allele of the identified association. n shows the number of accessions carrying each allele.


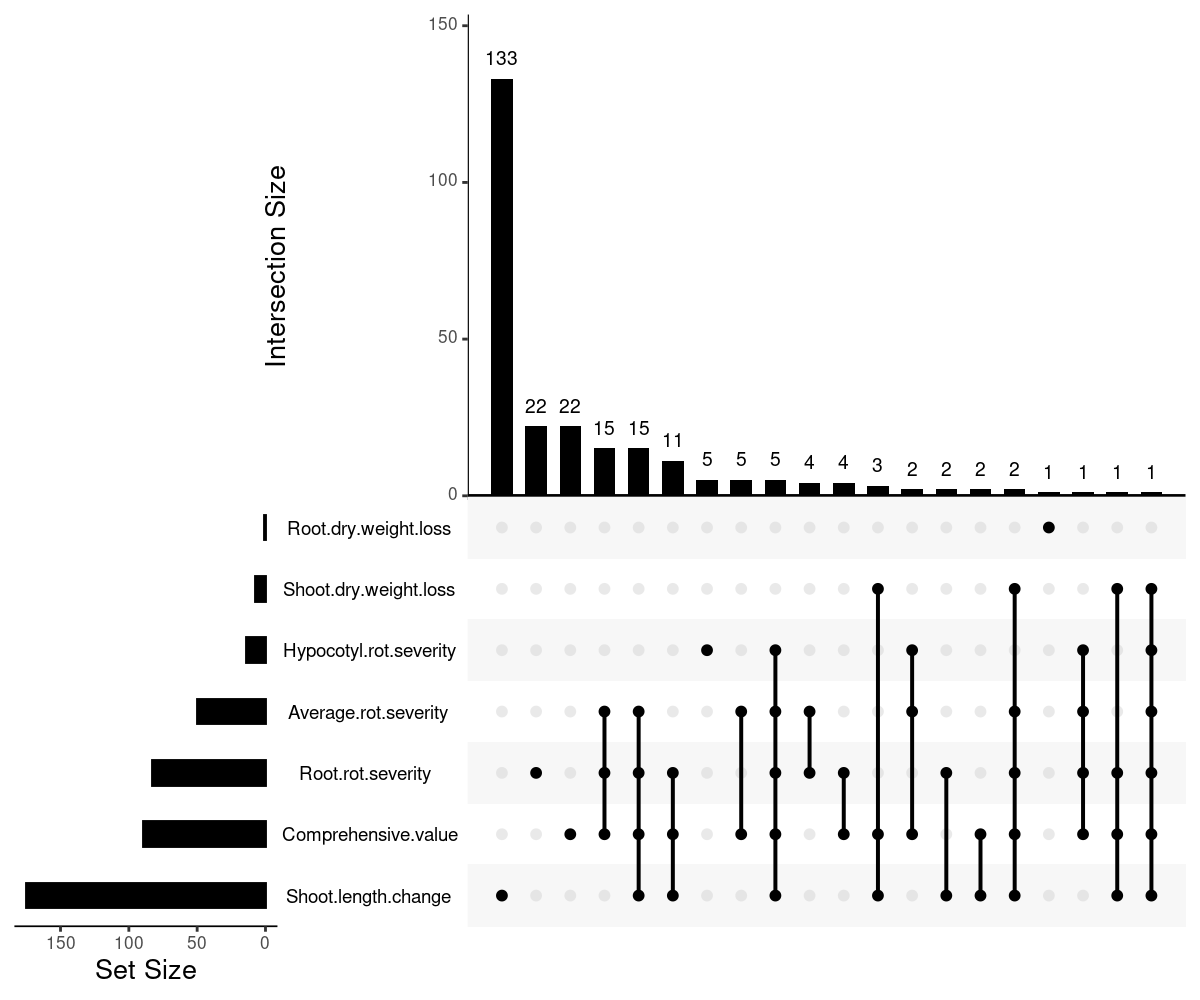

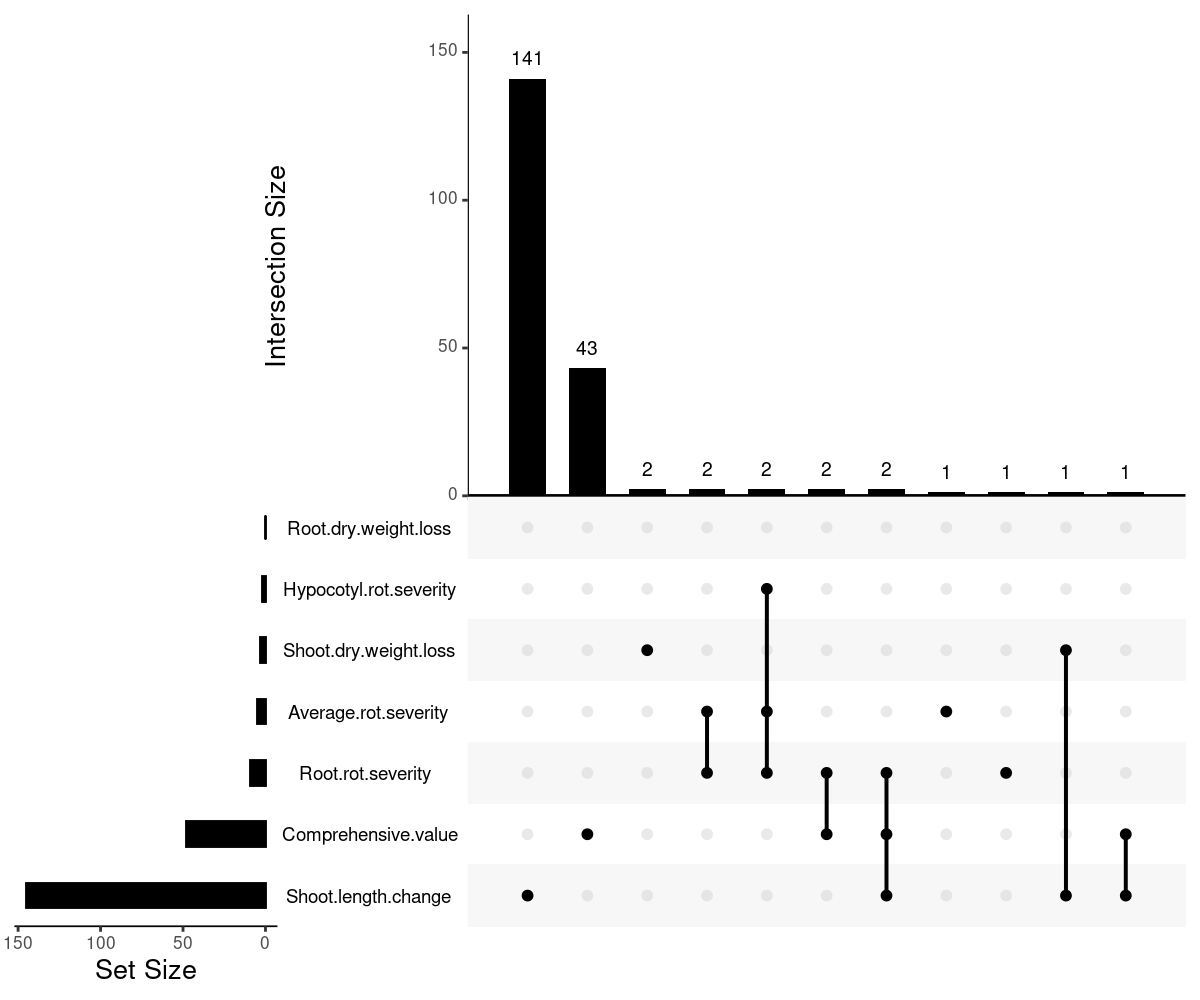


b

a

**Figure S17** UpSet plots of Genome-wide association study (GWAS) loci for Fusarium root rot (FRR) resistance under the 2I treatment (a) and the 4I treatment (b). The bar plots on the left represent the total numbers of associated signals identified for each FRR trait. The bar plots on the top indicate the numbers of unique and overlapping associated signals among these FRR traits.


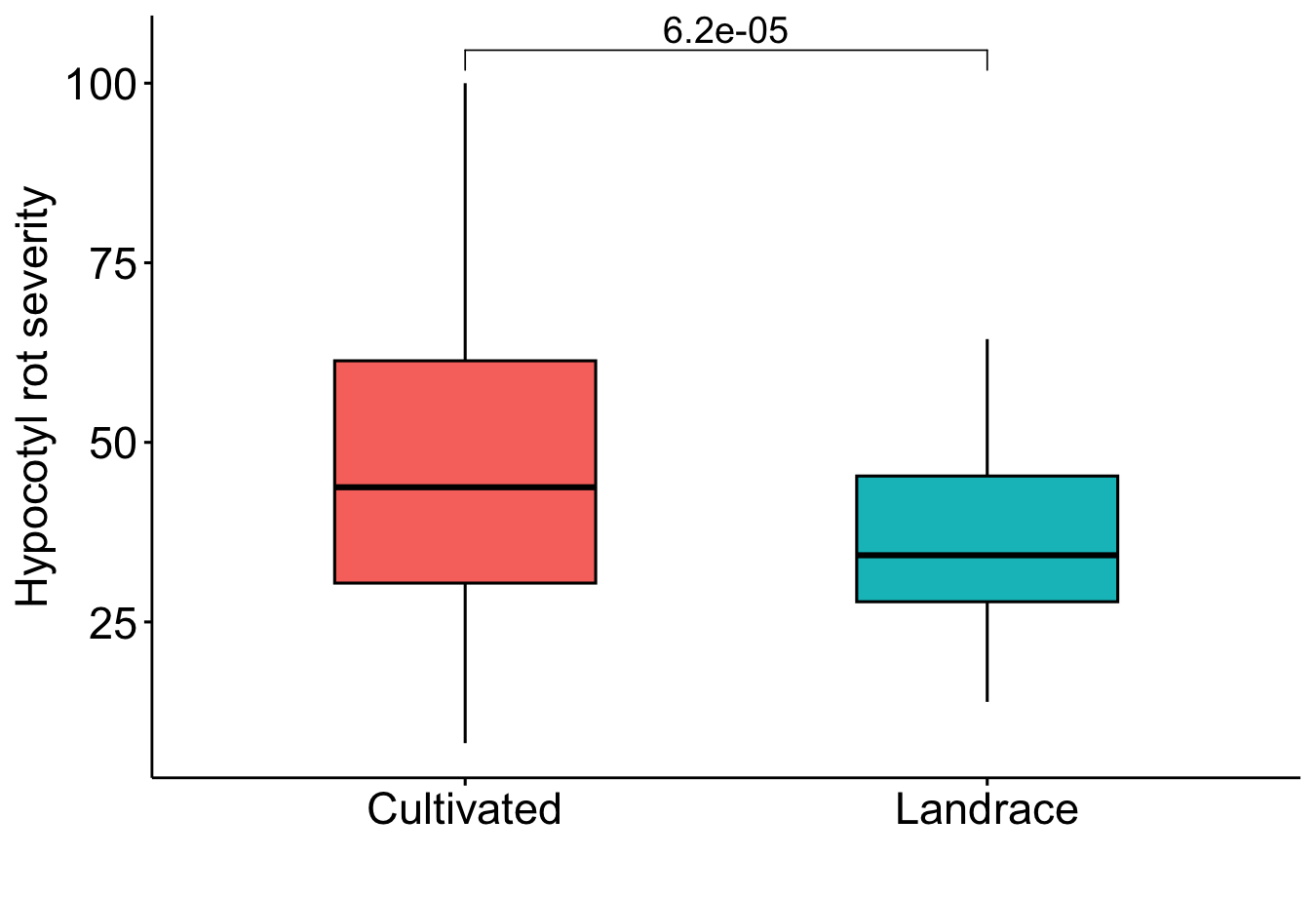

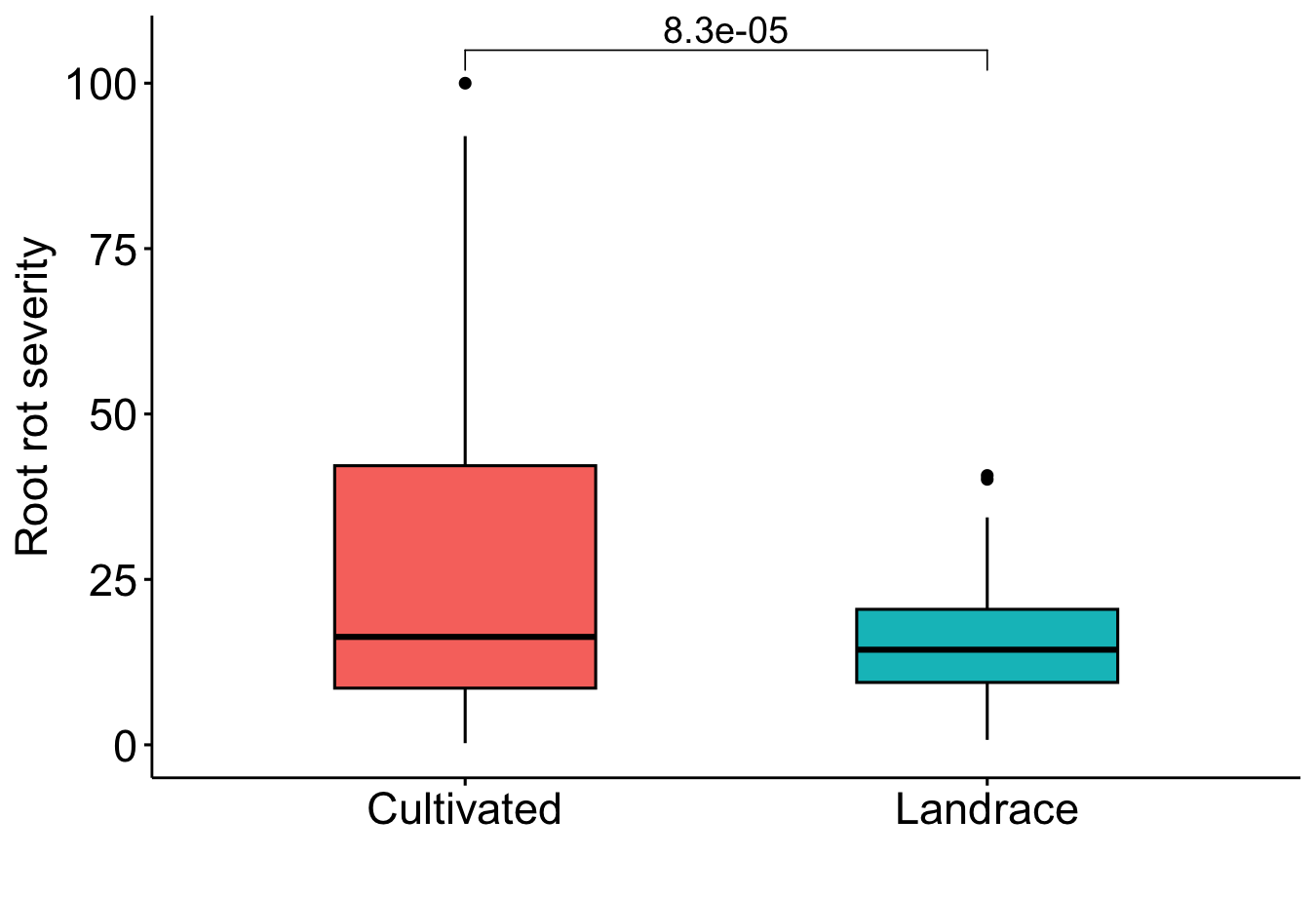

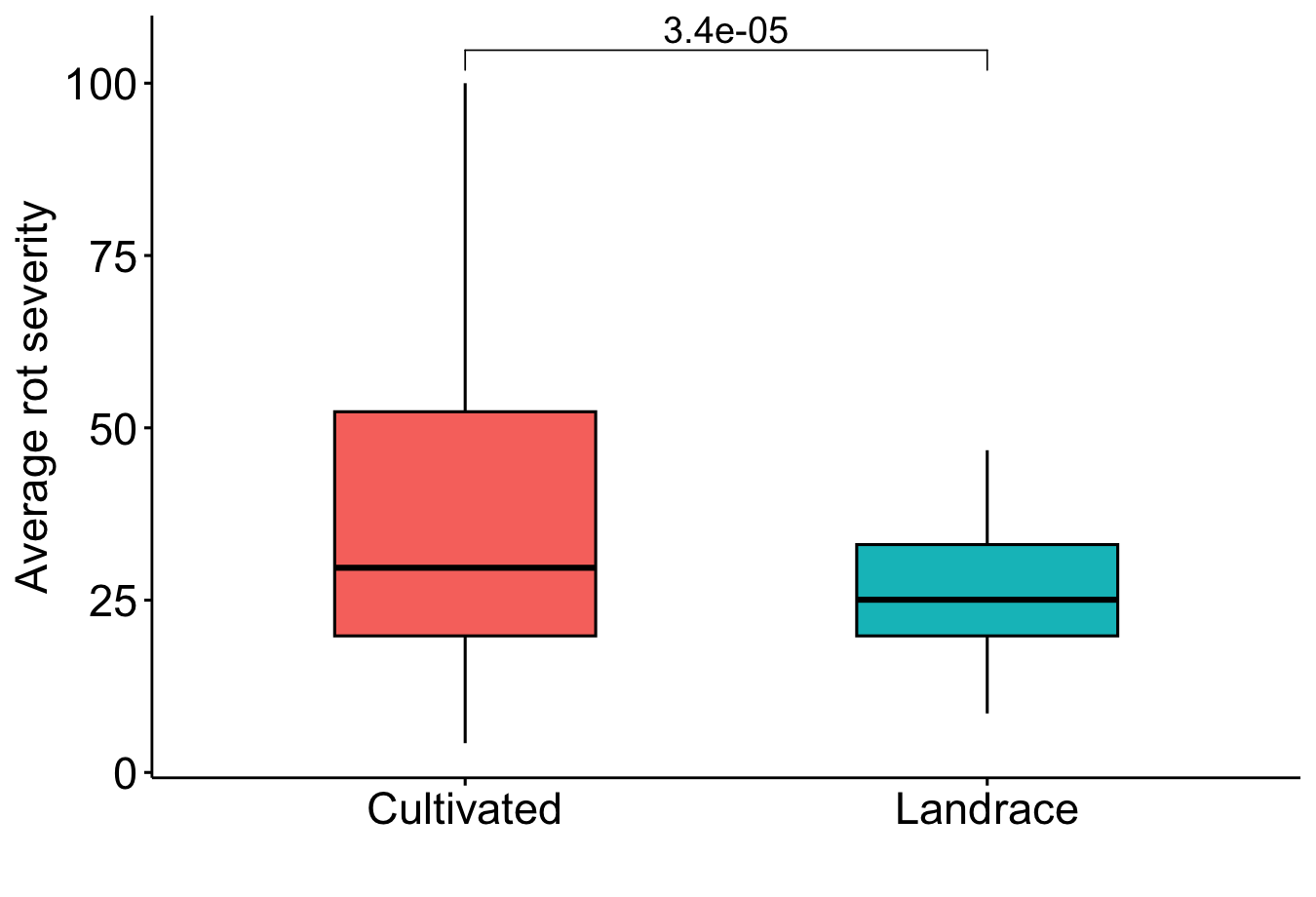

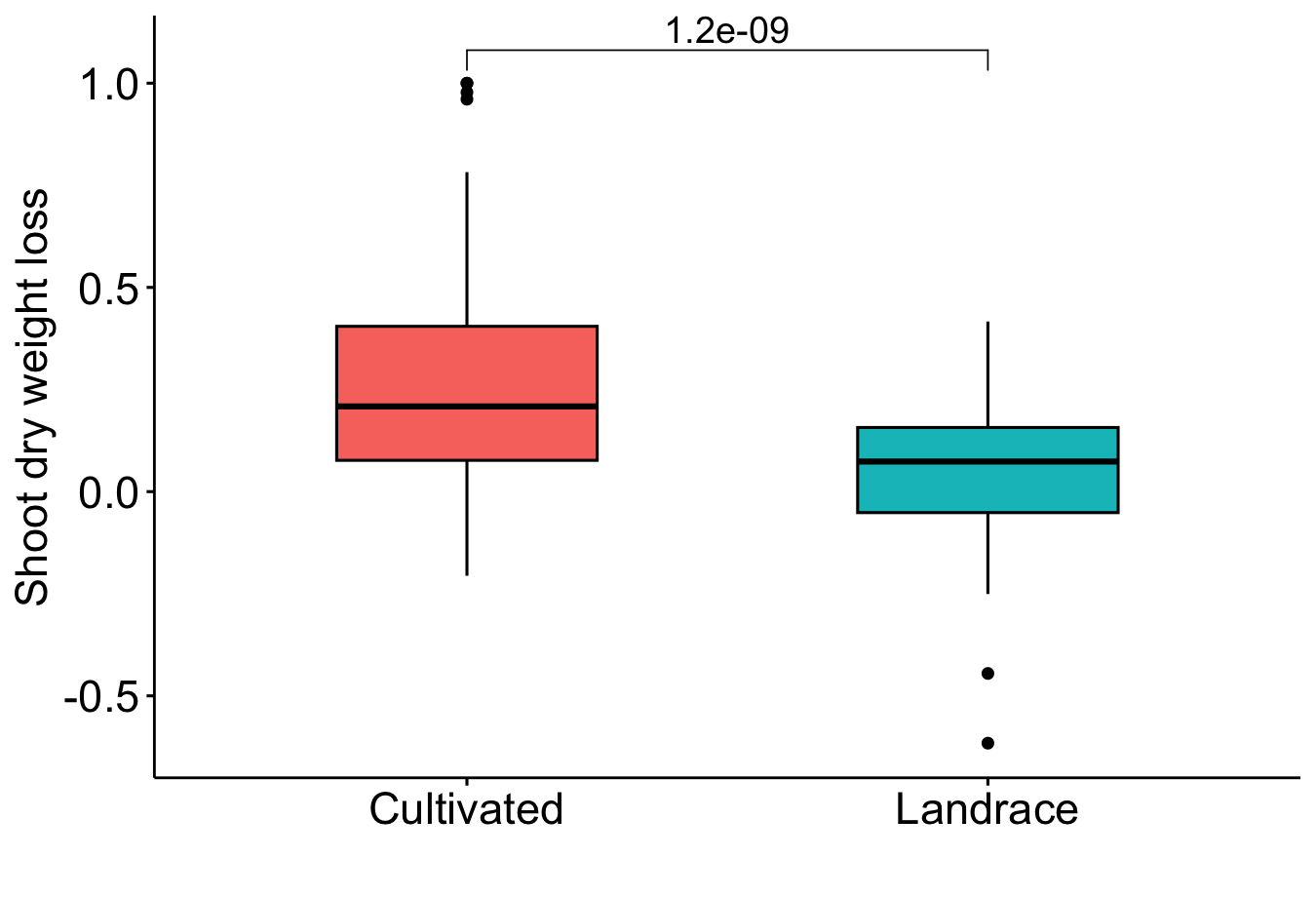


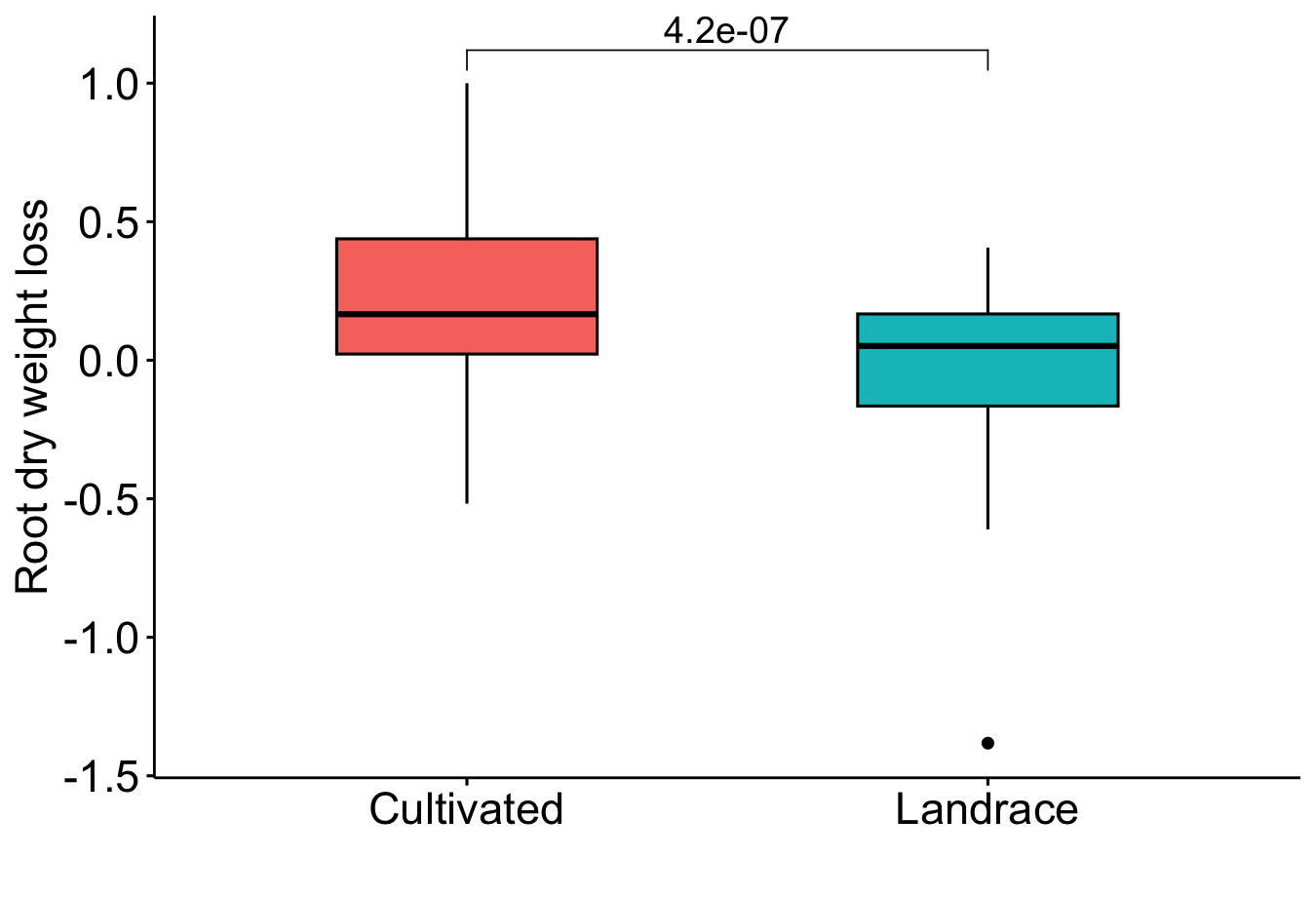

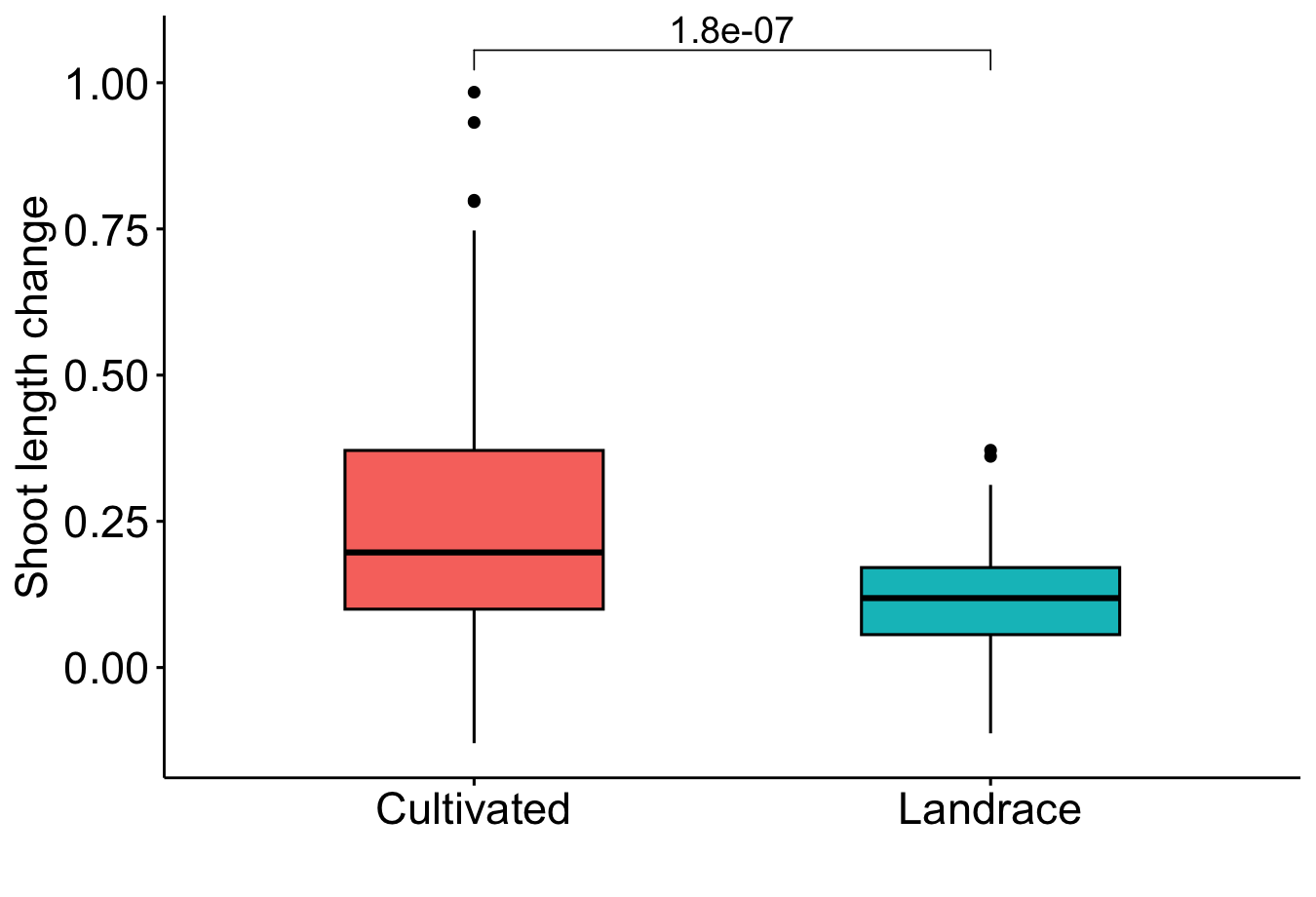


**Figure S18** Phenotypic differences of Fusarium root rot traits (2I) between *L. culinaris* landrace and cultivated accessions. Box limits: upper and lower quartiles, center lines: medians, whiskers: 1.5× interquartile ranges, dots: outlines. *P* values was estimated using *t*-test.


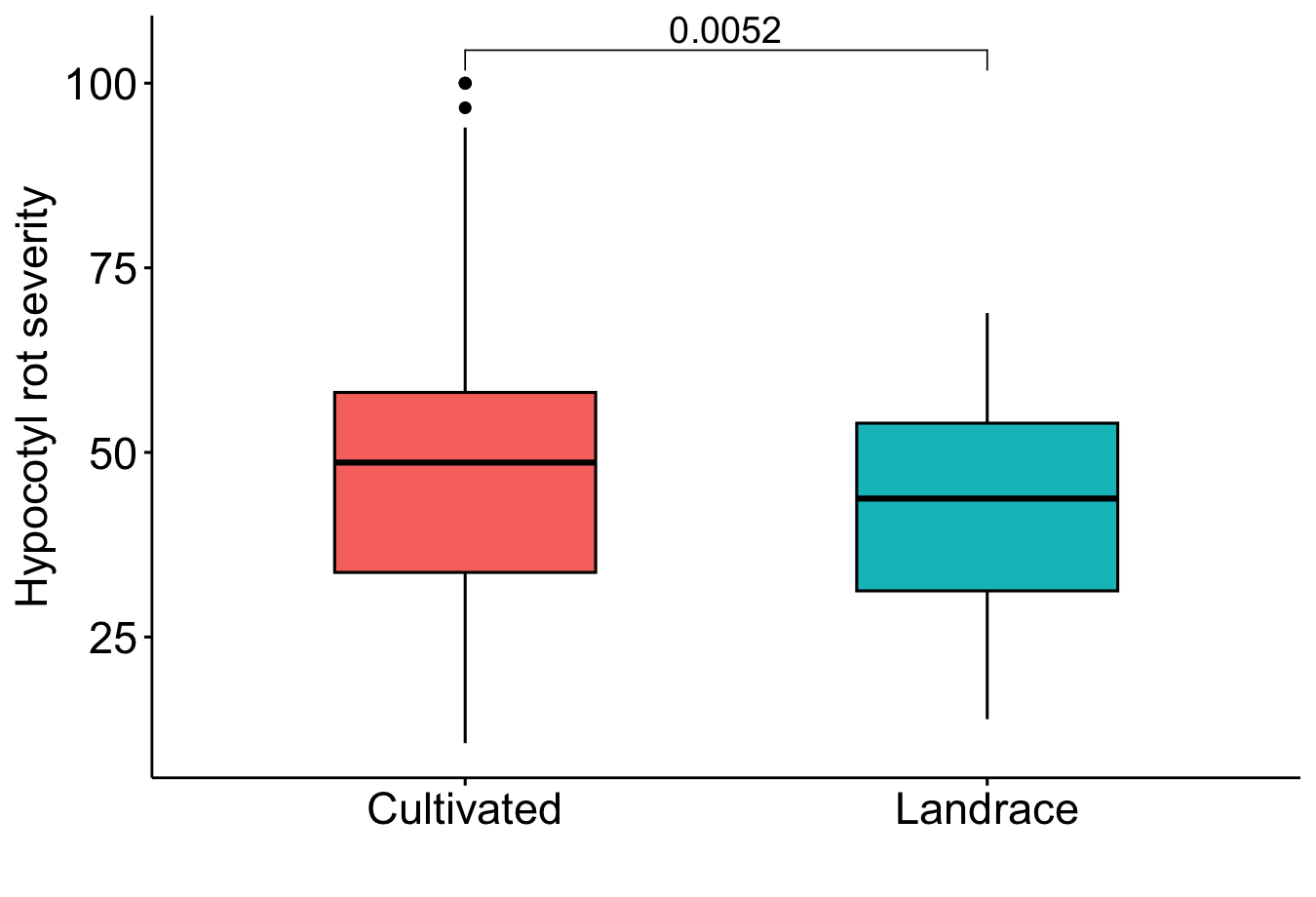

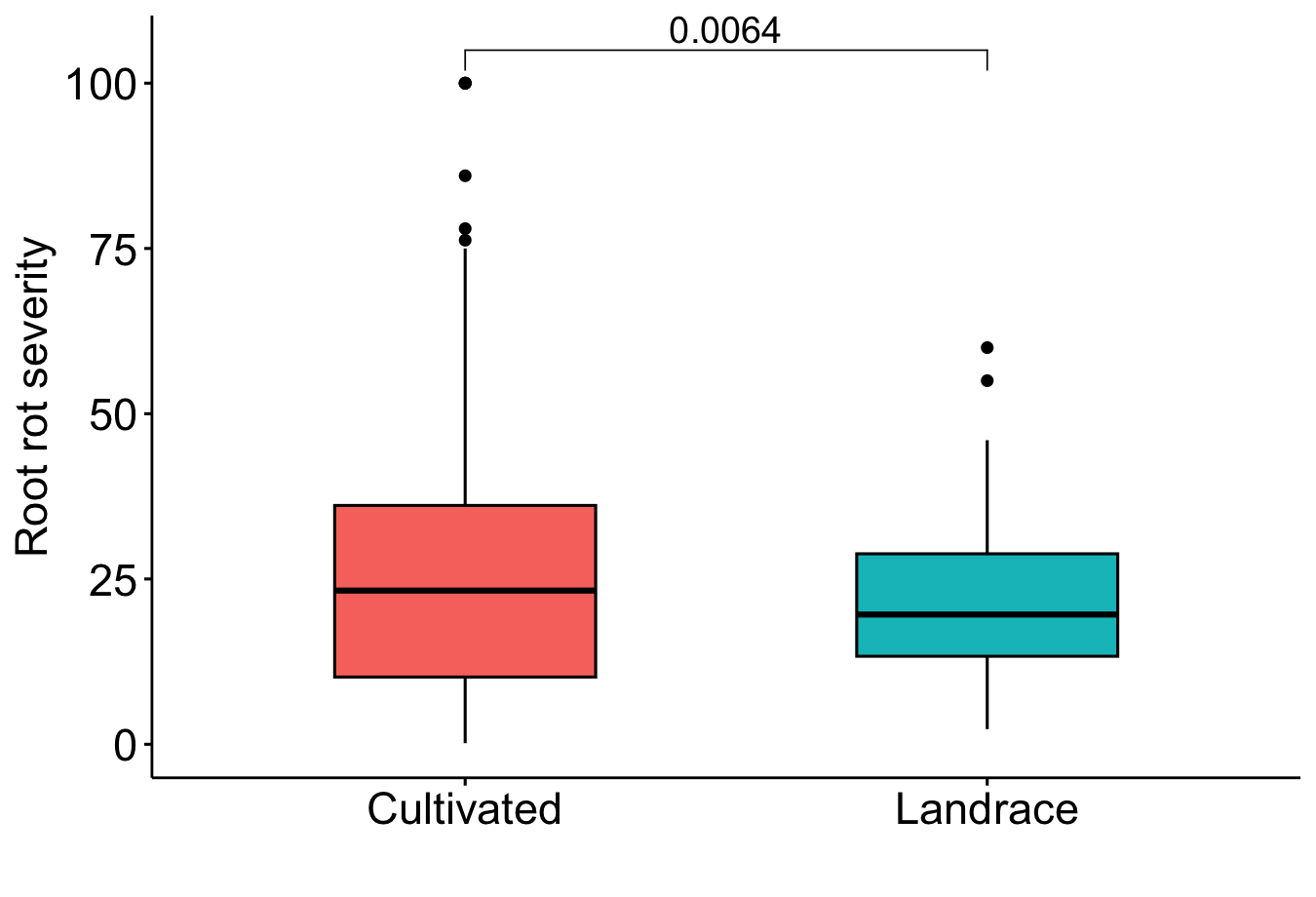

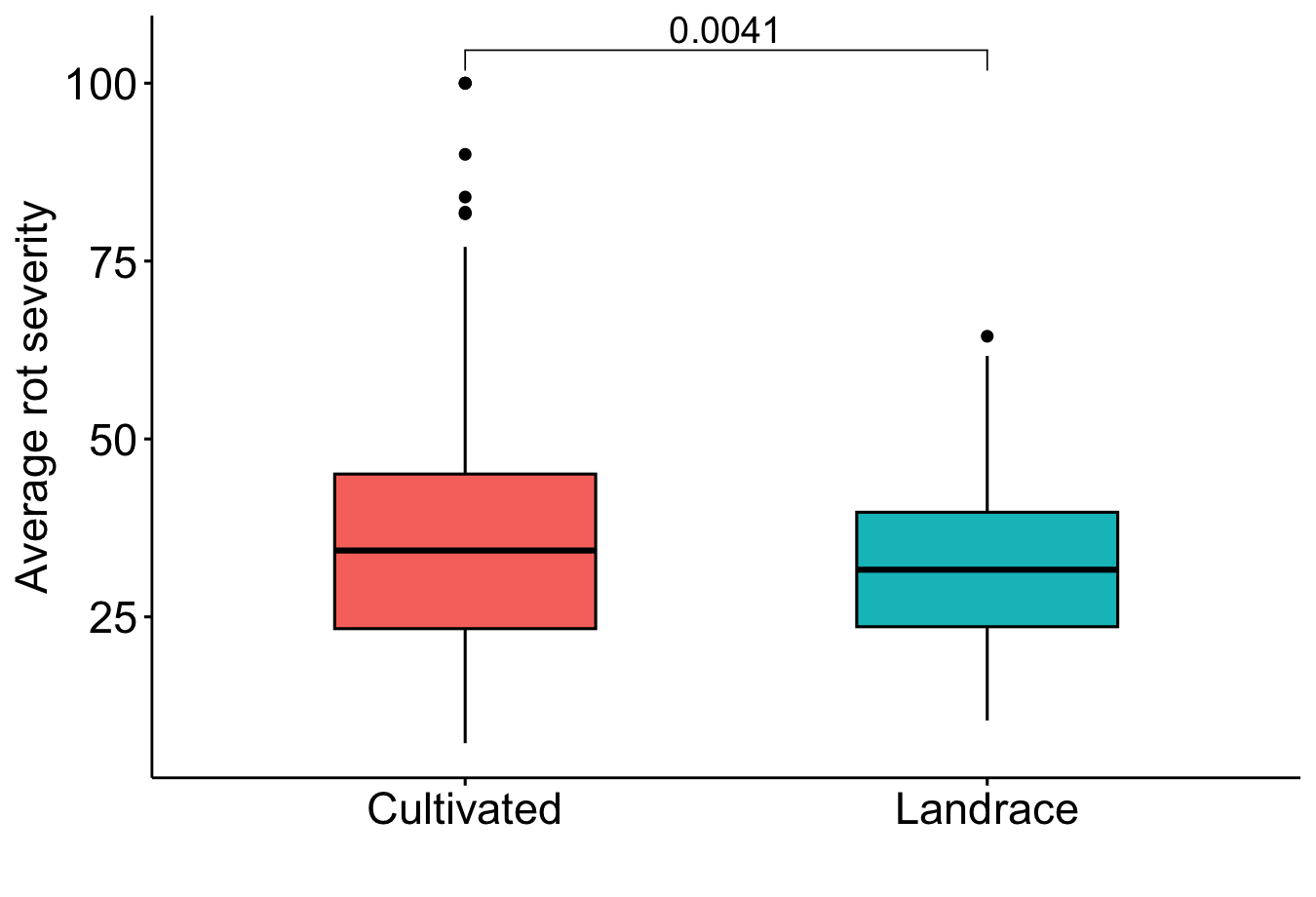

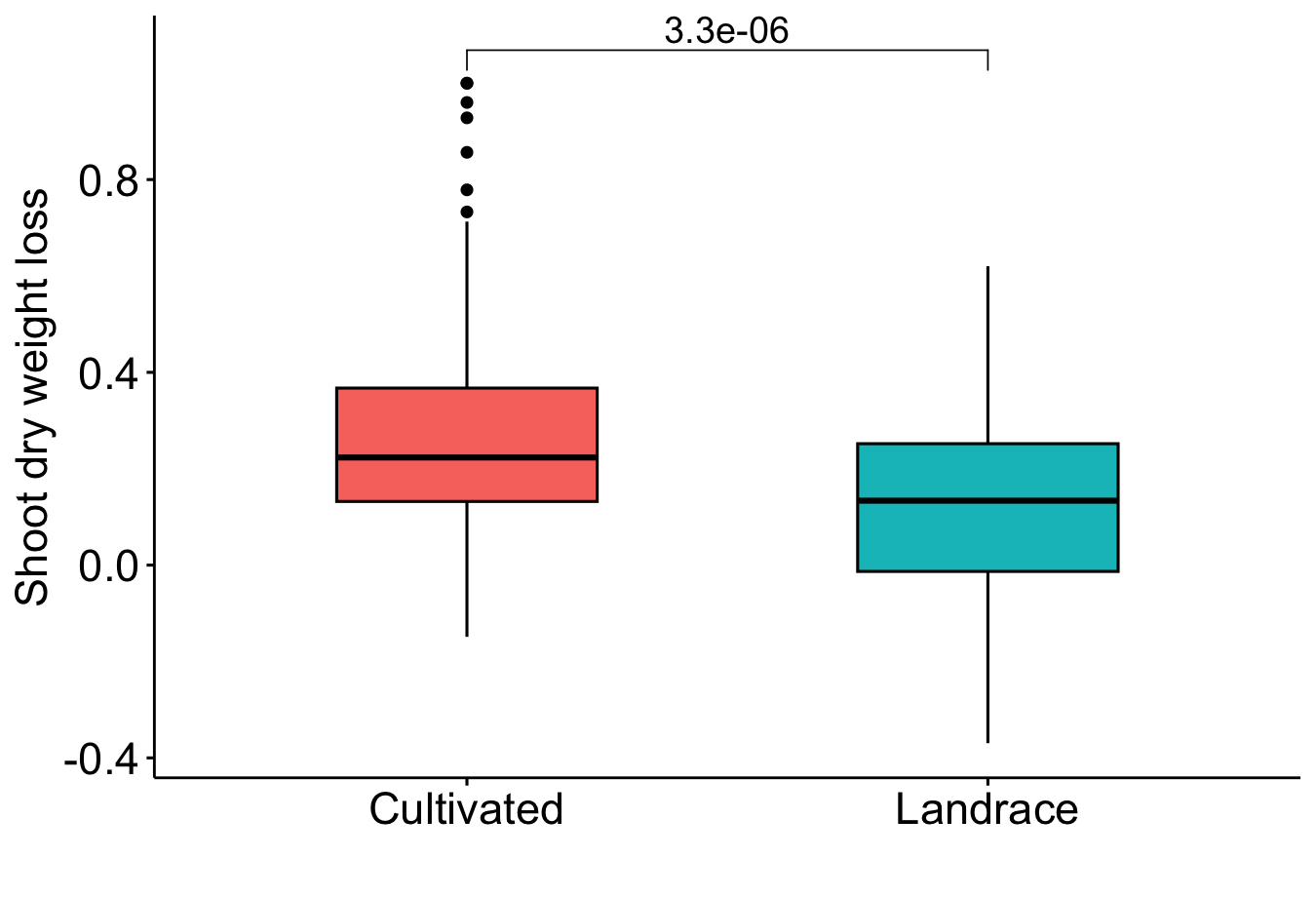


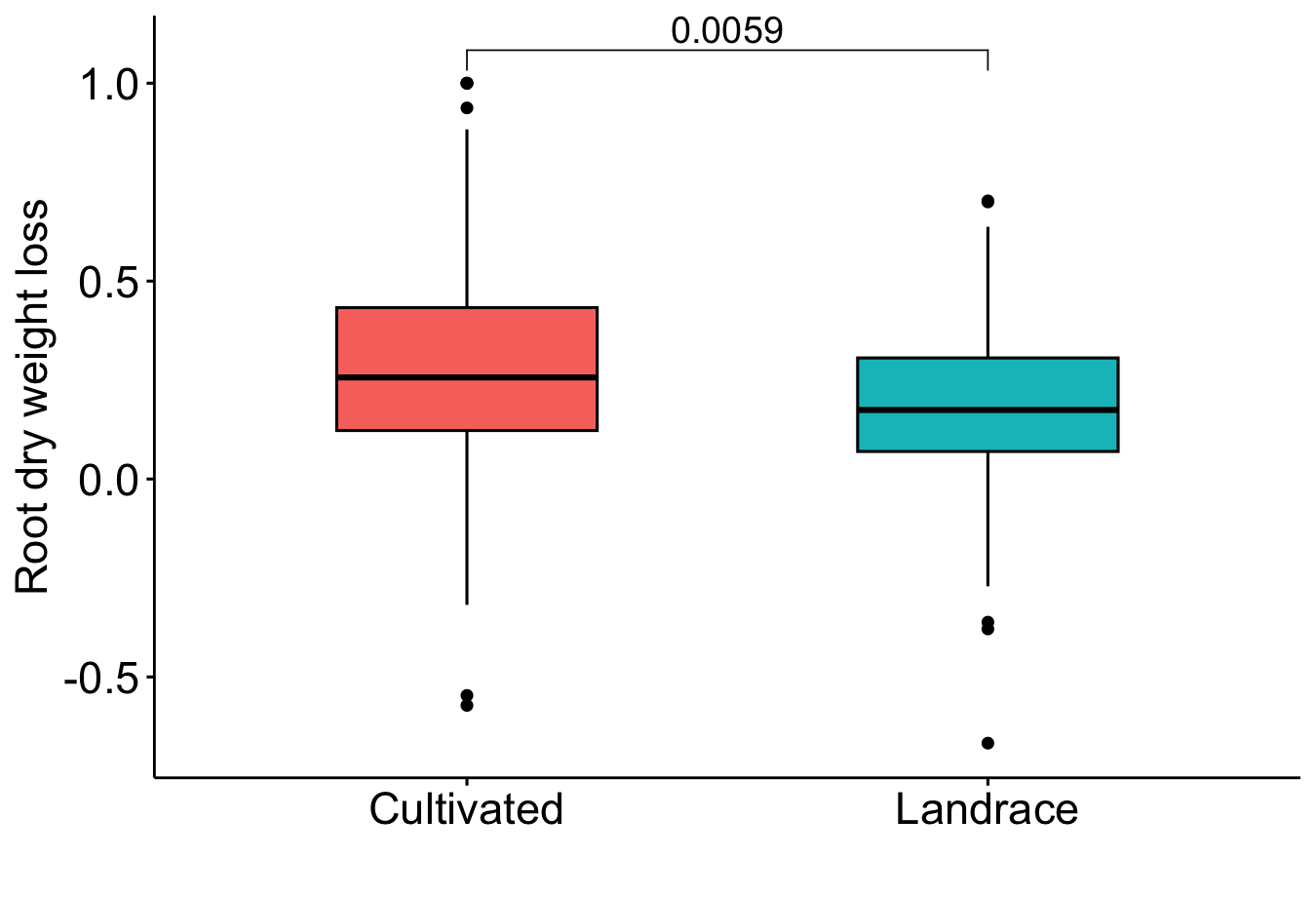

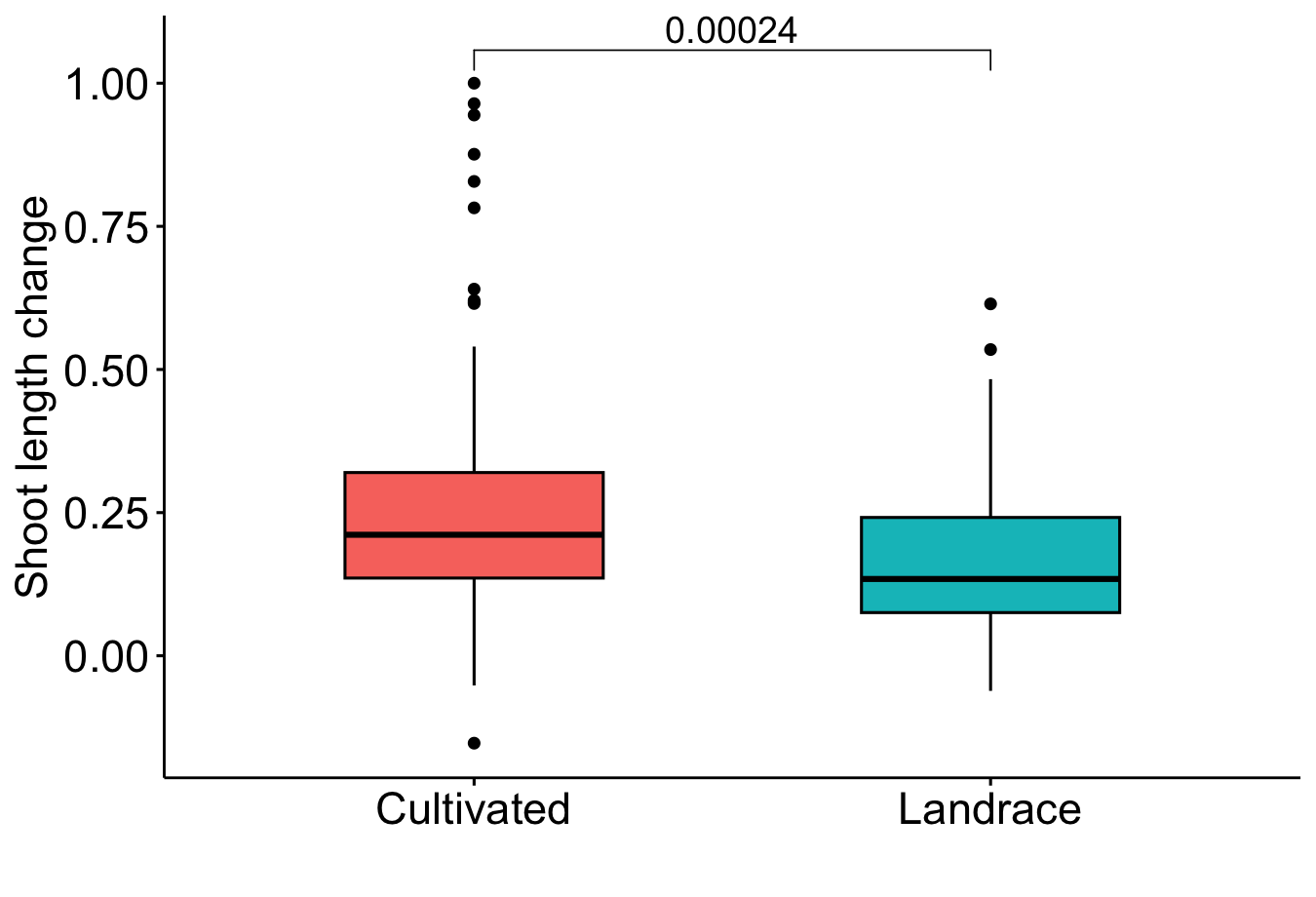


**Figure S19** Phenotypic differences of Fusarium root rot traits (4I) between *L. culinaris* landrace and cultivated accessions. Box limits: upper and lower quartiles, center lines: medians, whiskers: 1.5× interquartile ranges, dots: outlines. *P* values was estimated using *t*-test.


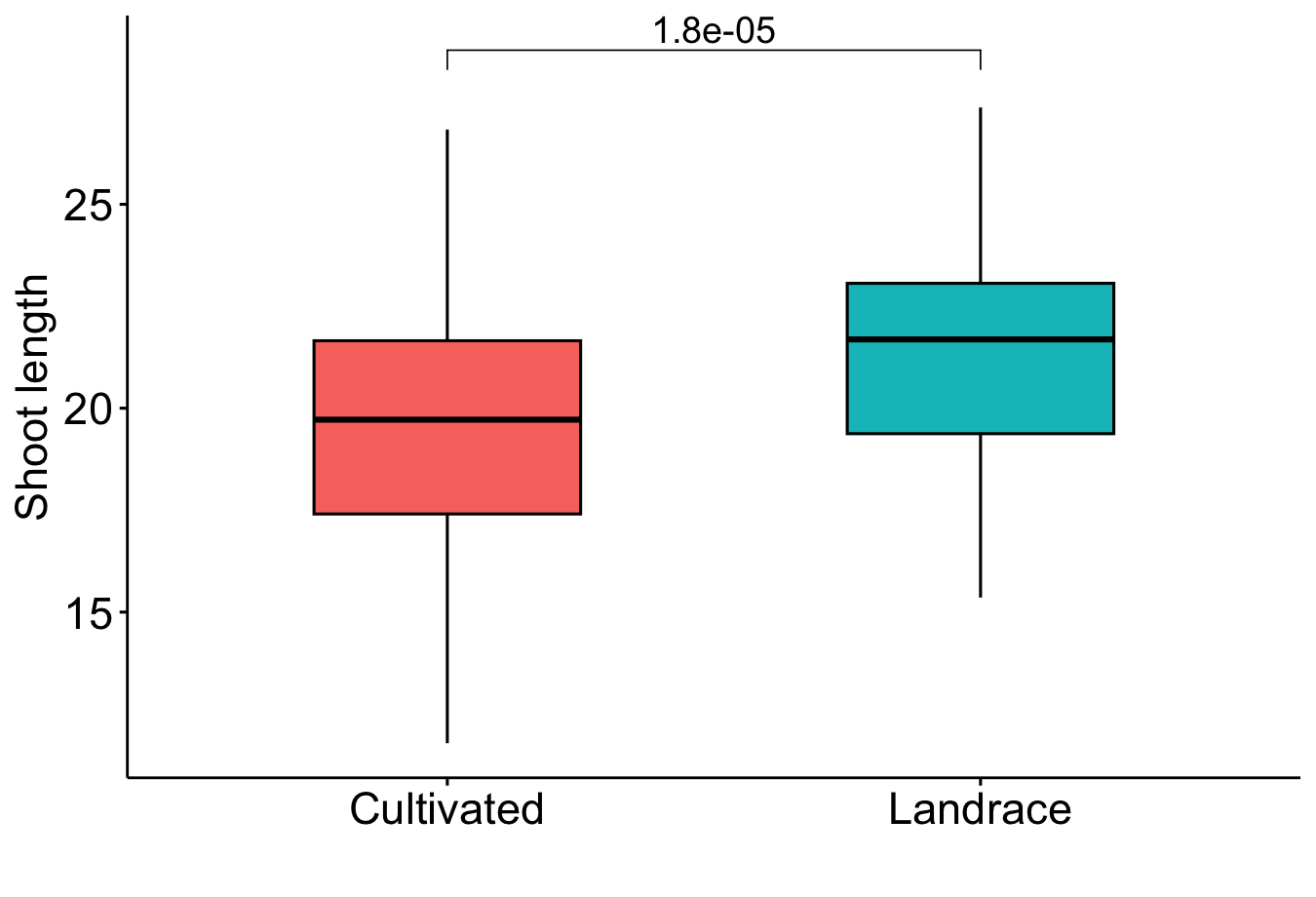

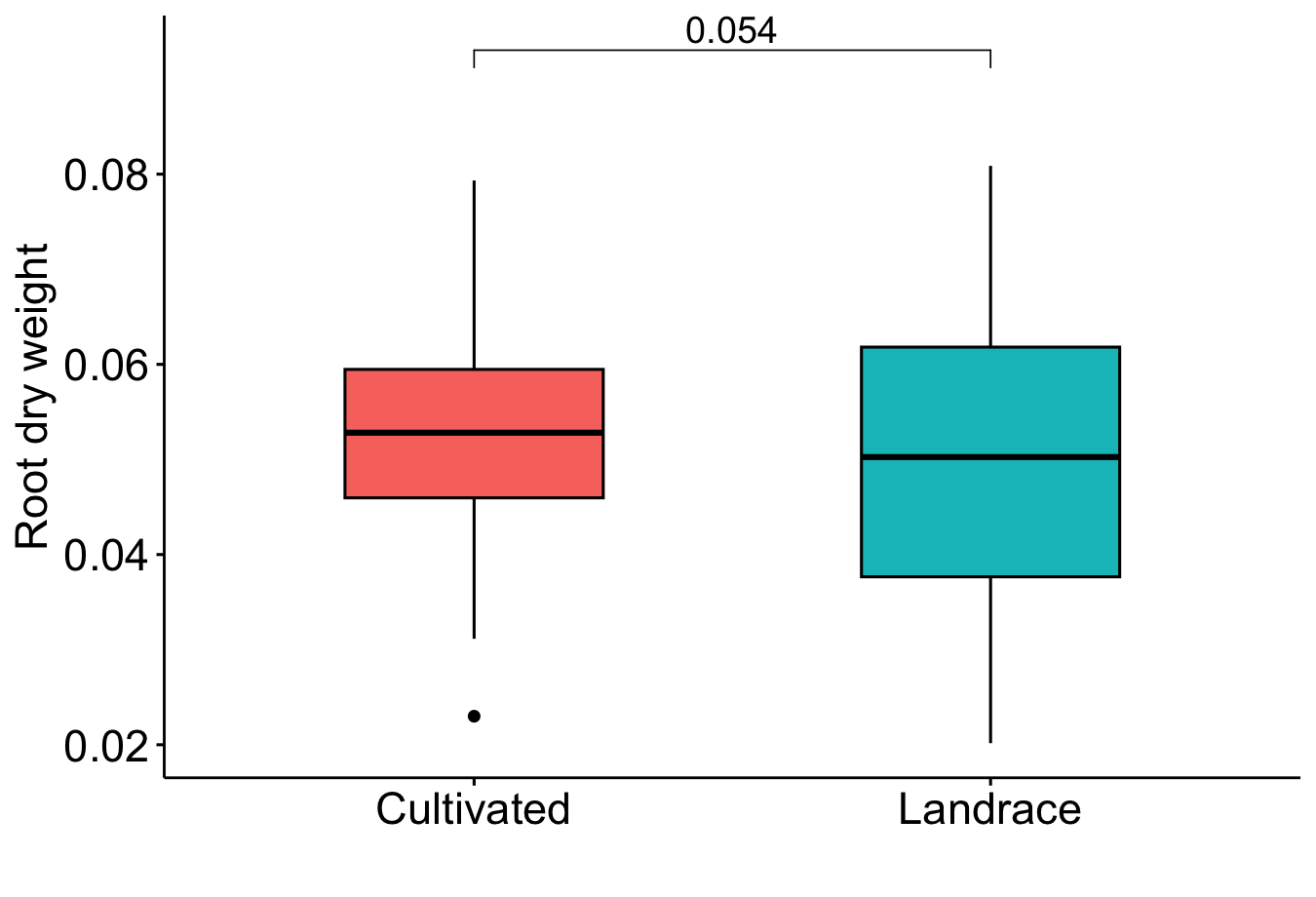

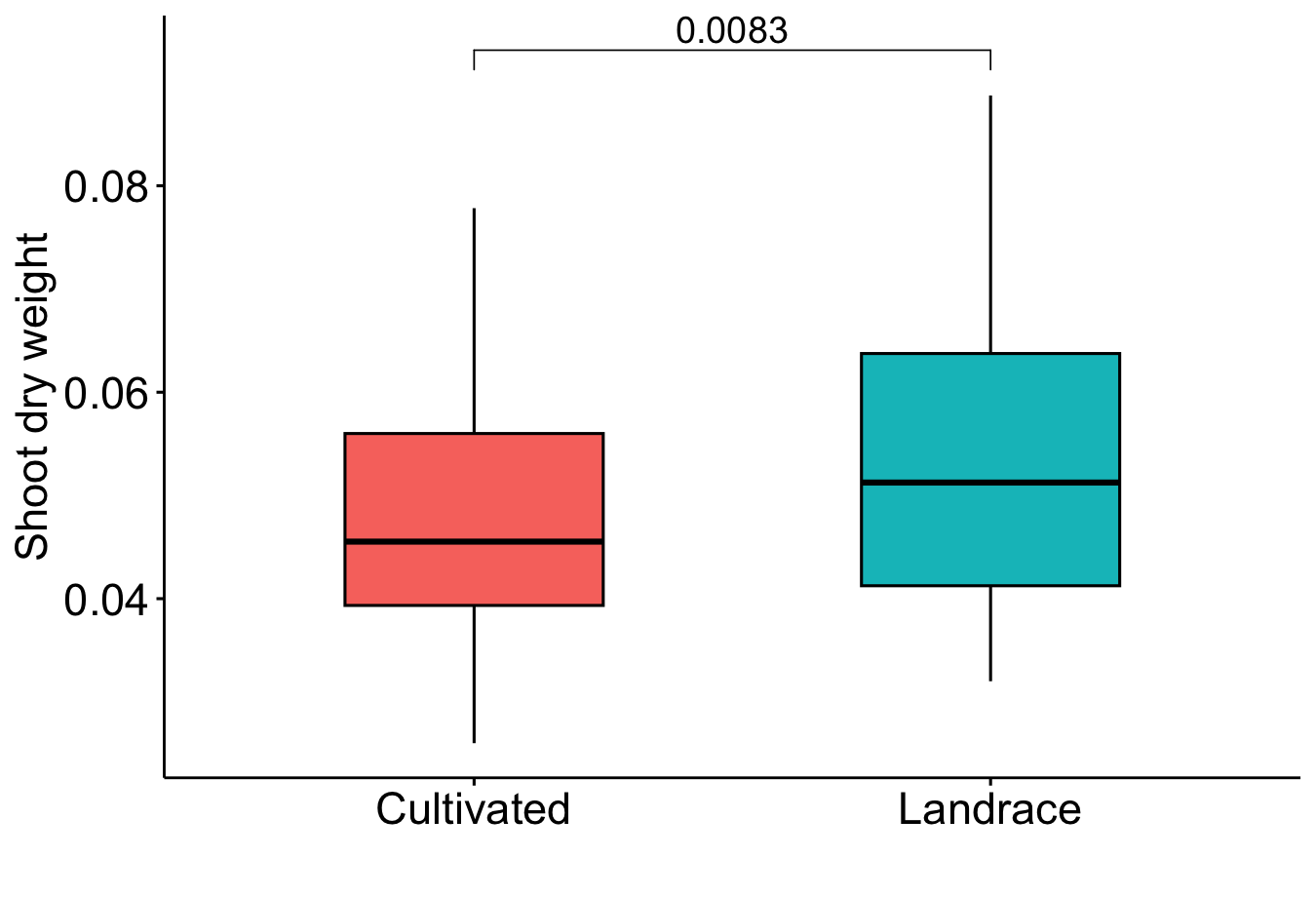


**Figure S20** Phenotypic differences of shoot length and root and shoot dry weight between *L. culinaris* landrace and cultivated accessions. Box limits: upper and lower quartiles, center lines: medians, whiskers: 1.5× interquartile ranges, dots: outlines. *P* values was estimated using *t*-test.

**Figure S21** GWAS result from the analysis of shoot weight loss and comprehensive value measured under the 2I treatment. The red horizontal dashed line represents the significance threshold (Bonferroni correction, α = 0.05). The lower panel shows the local Manhattan plot surround the significantly associated SNP (chr7:7779810) in the gene *Lcu.2RBY.7g004590* (*TIR-NBS-LRR*) on chromosome 7.

**
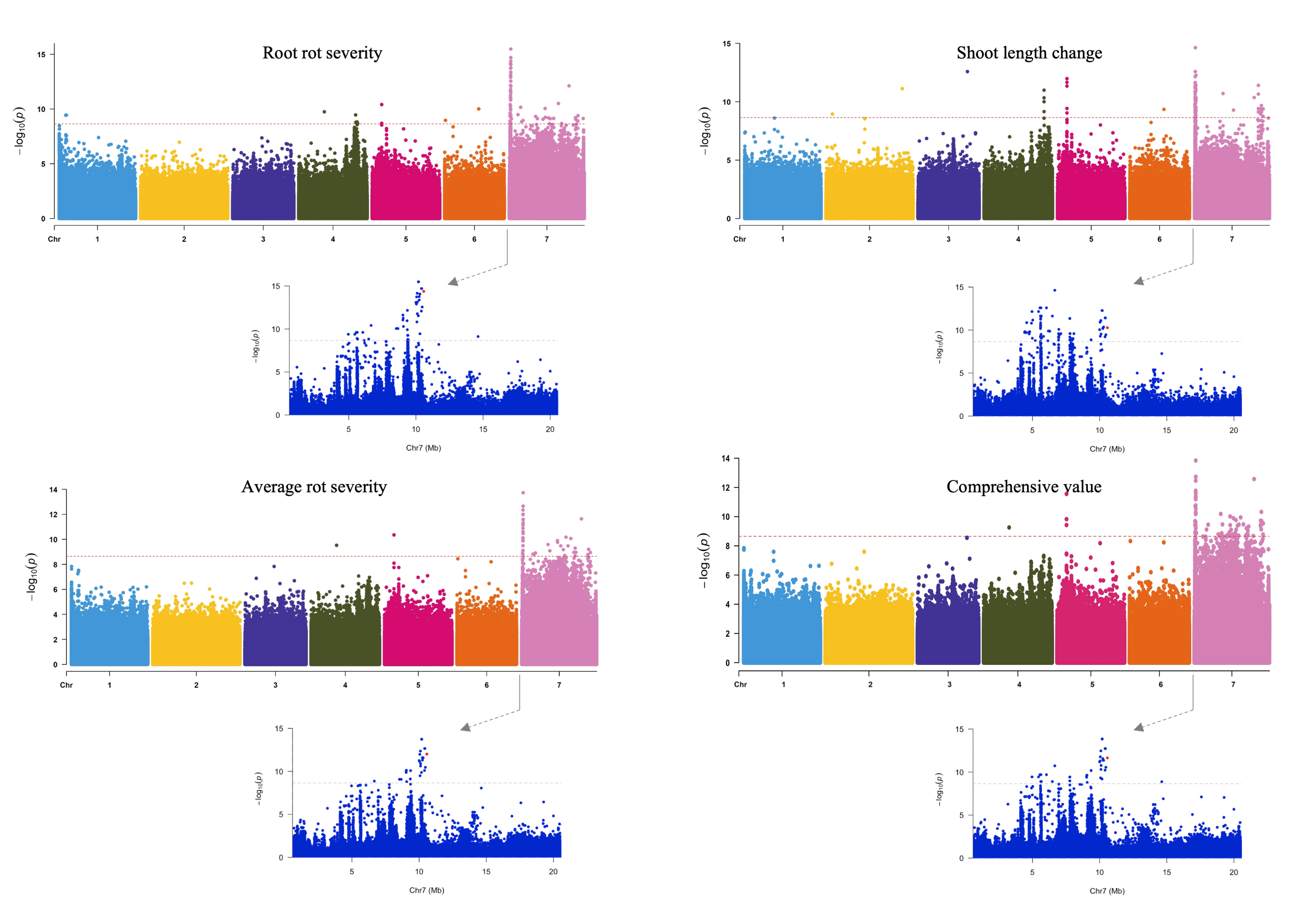
**

**Figure S22** GWAS result from the analysis of root rot severity, average rot severity, shoot length change and comprehensive value measured under the 2I treatment. The red horizontal dashed line represented the significance threshold (Bonferroni correction, α = 0.05). The lower panel shows the local Manhattan plot surround the significantly associated SNP (chr7: 10576840) in the gene *Lcu.2RBY.7g005600* (*ABP19a*) on chromosome 7.

**Figure S23** Haplotype analysis on the candidate gene *ABP19a* (*Lcu.2RBY.7g005600*). **(a)** Gene structure and the major eight haplotypes of *ABP19a* (*Lcu.2RBY.7g005600*). **(b)** Phenotypic differences for the six FRR traits (hypocotyl rot severity, root rot severity, average rot severity, shoot dry weight loss, root dry weight loss, and shoot length change) based on the haplotypes of *ABP19a* (*Lcu.2RBY.7g005600*). Box limits: upper and lower quartiles, center lines: medians, whiskers: 1.5× interquartile ranges, dots: outlines. The letters above the boxes indicate significant differences (*P* < 0.05, Tukey’s HSD test) between pairs of group comparison. **(c)** Frequency of eight haplotypes in three phylogeographic groups in *L. culinaris* landrace and four market type groups in *L. culinaris* cultivated accessions.

**
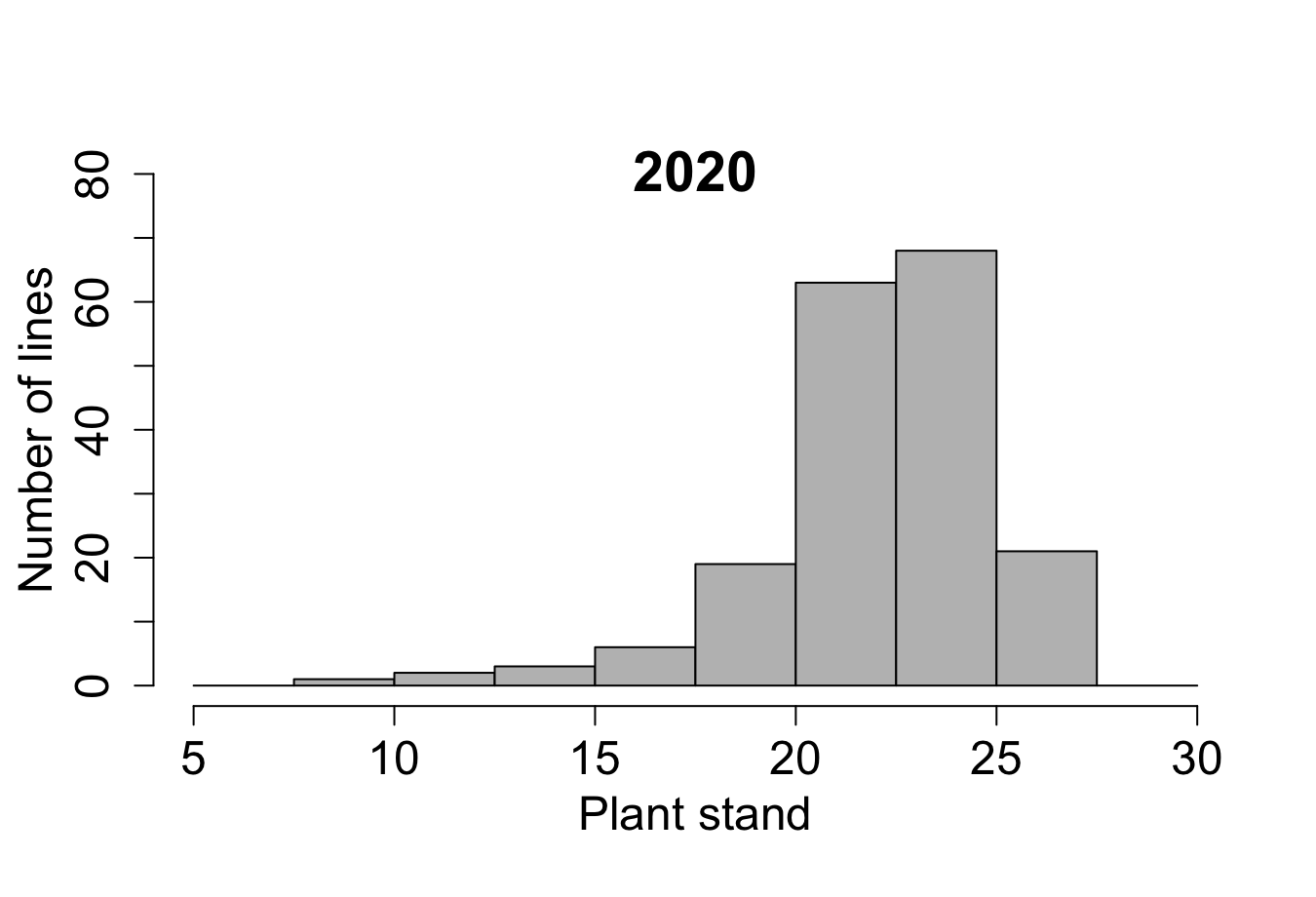
**
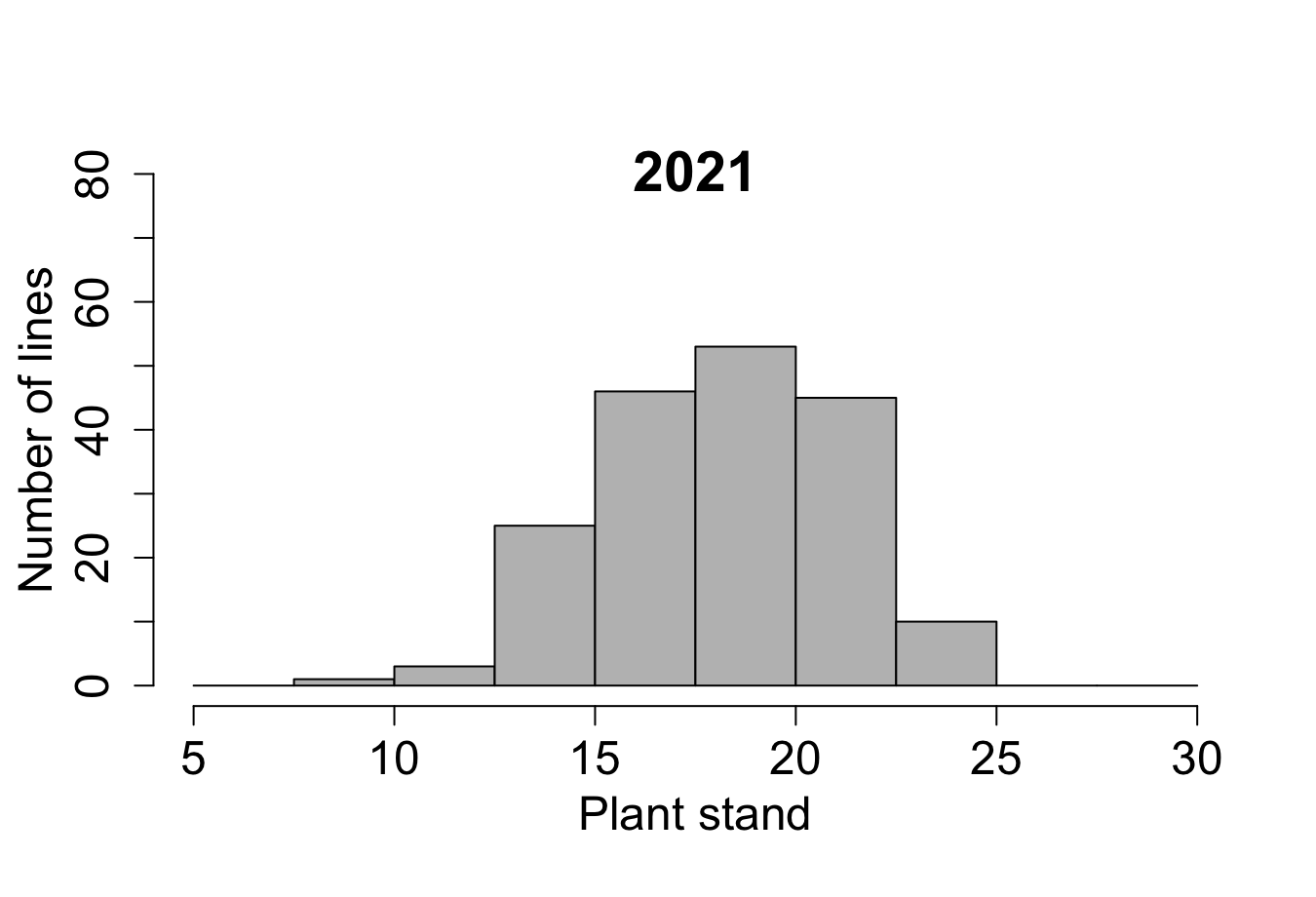
**
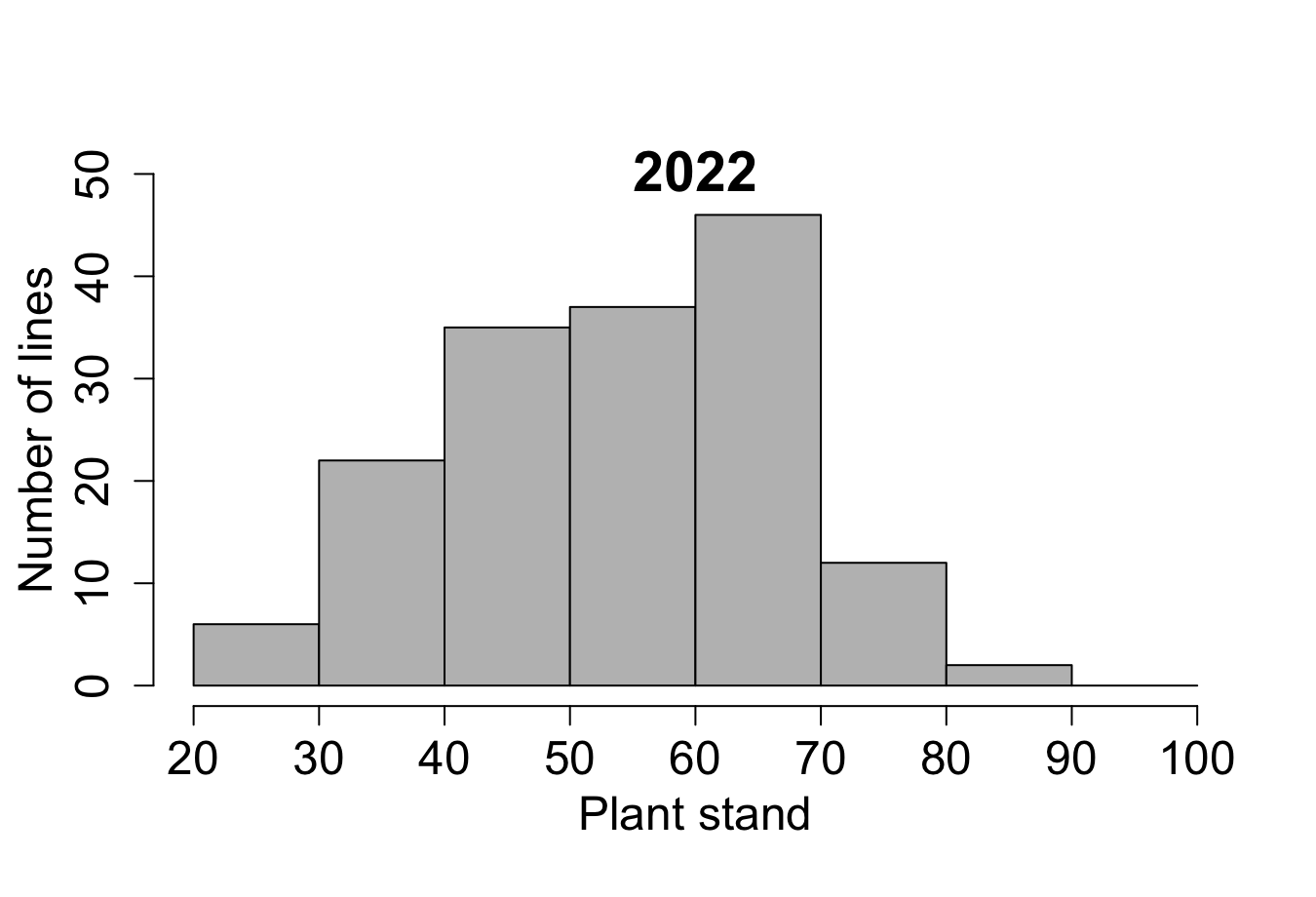
**

**
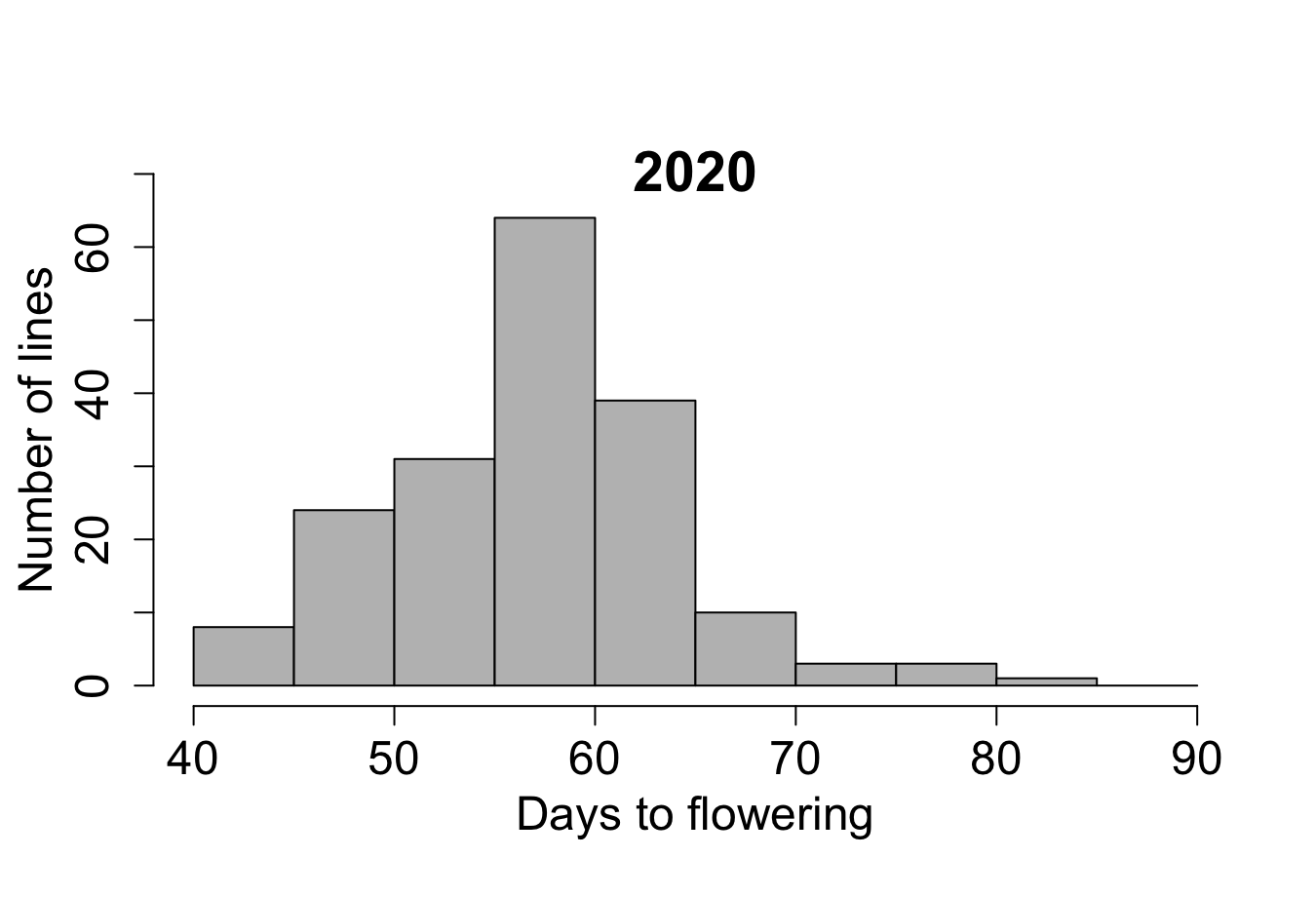

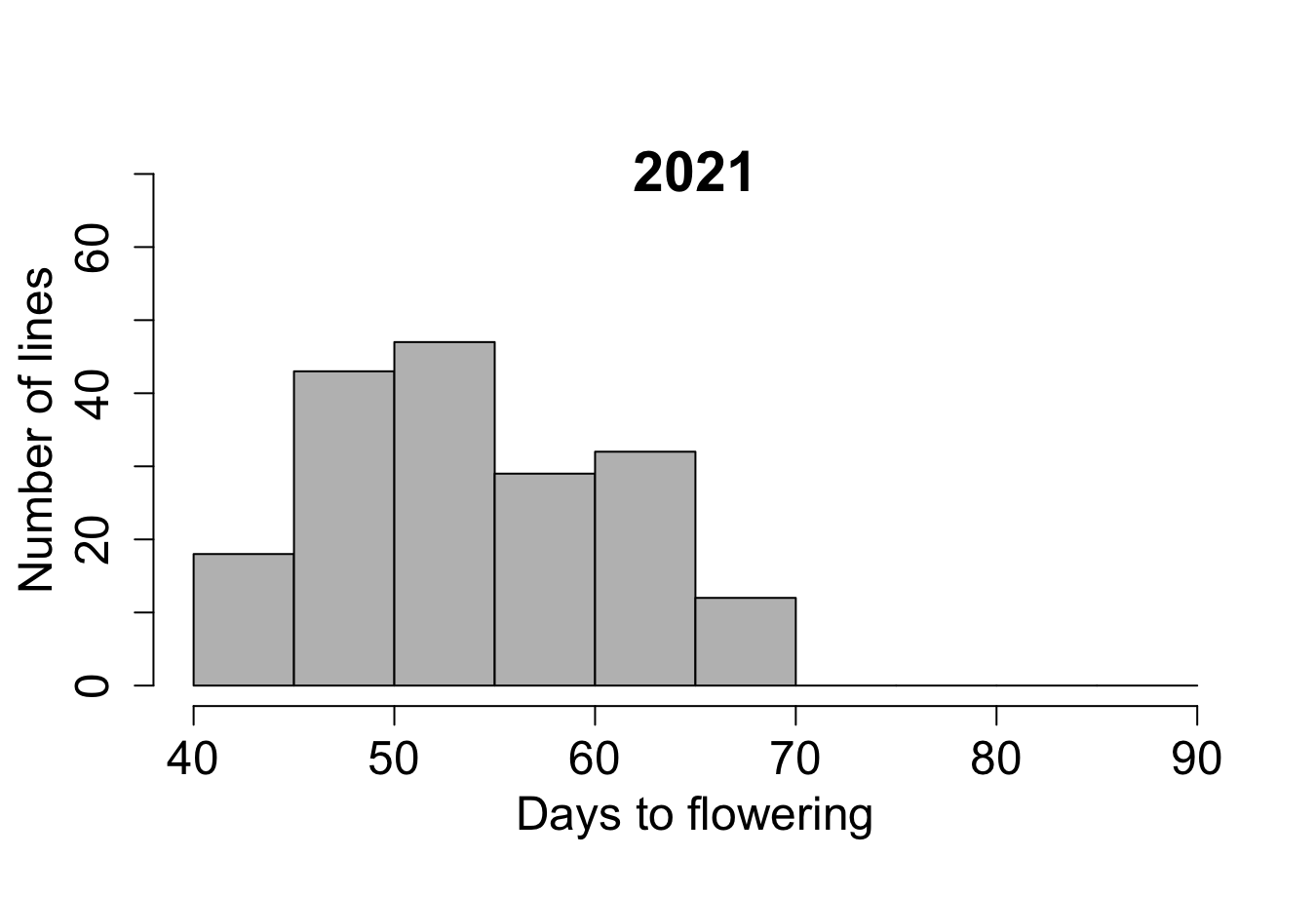

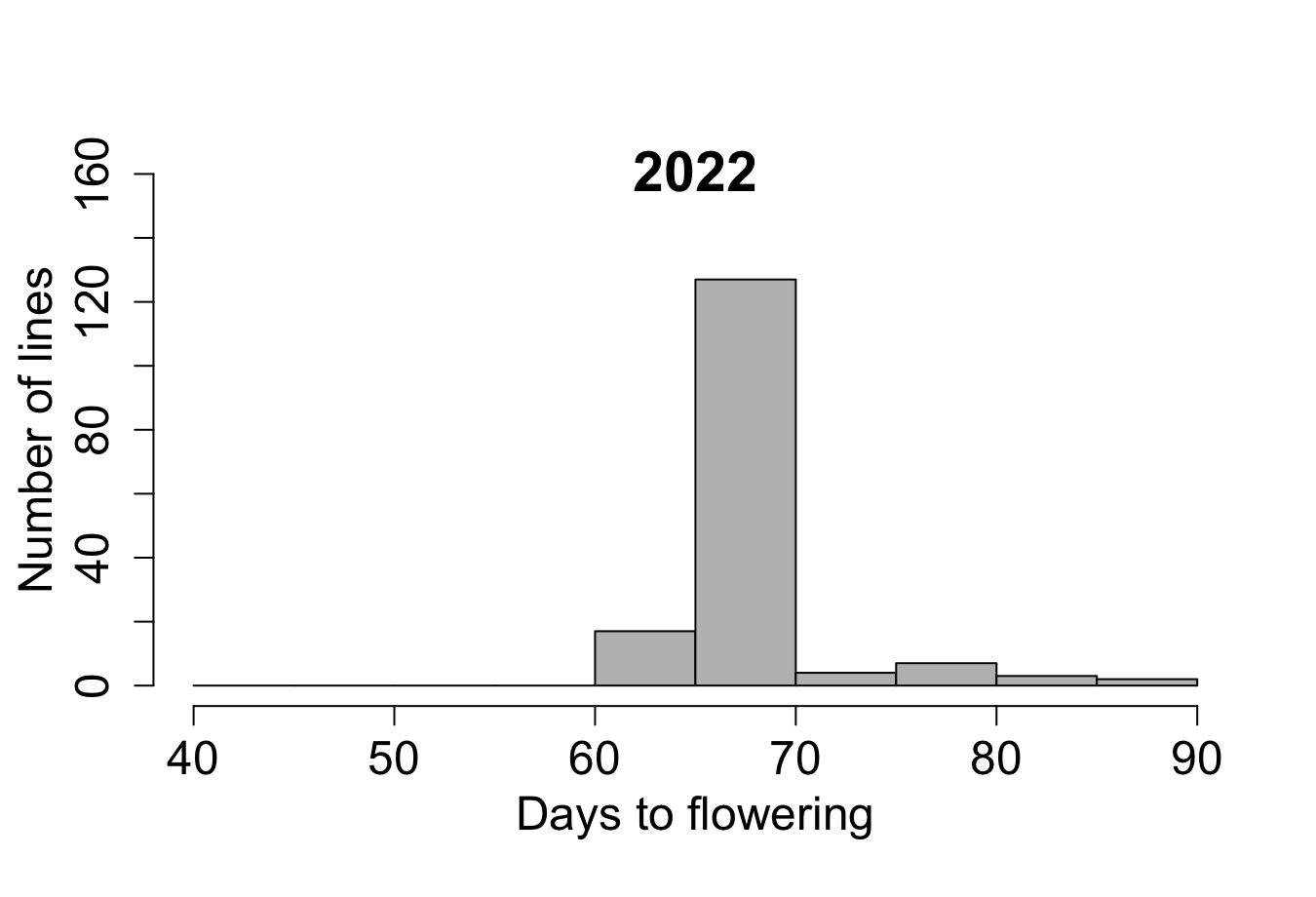
**

**
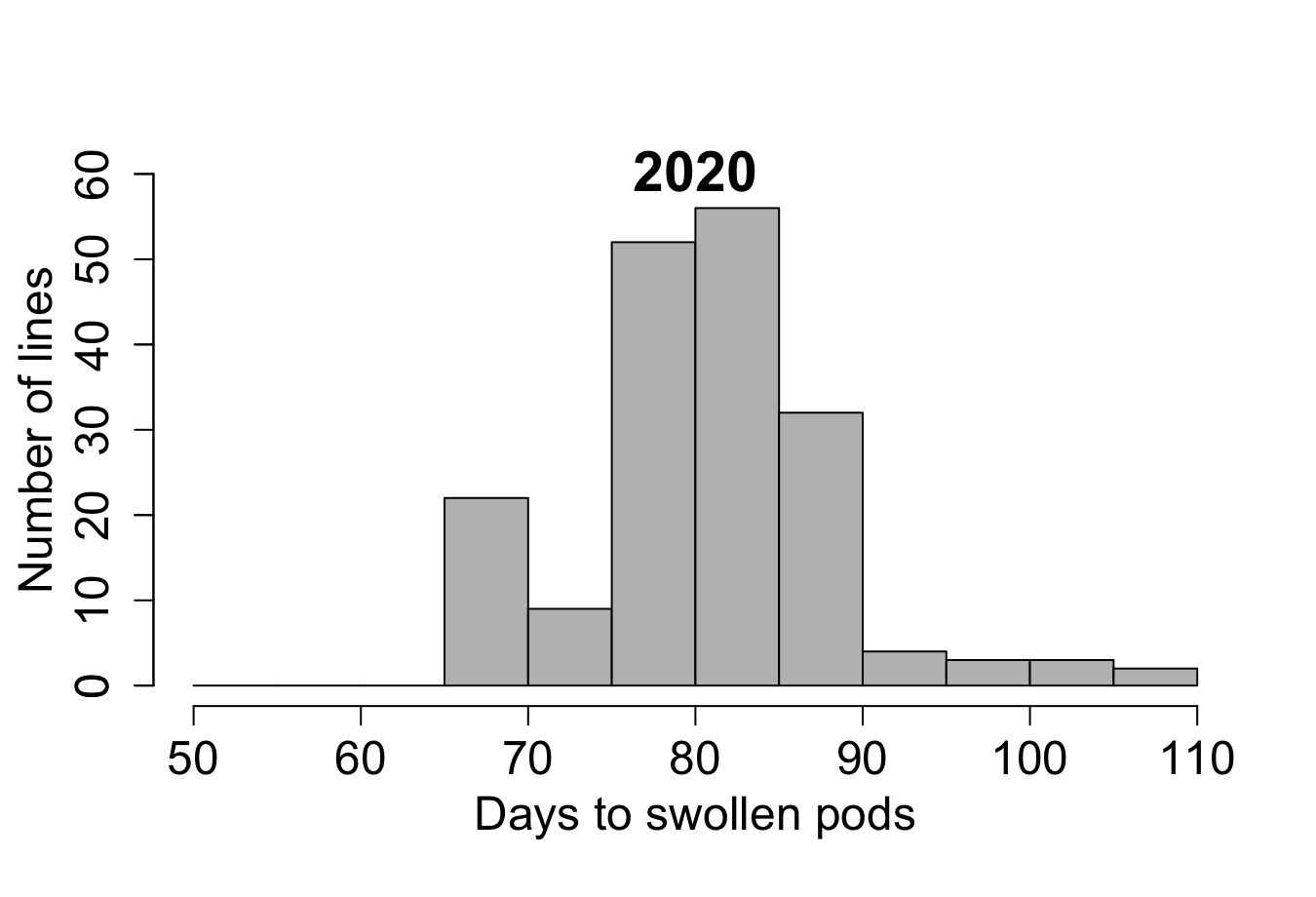

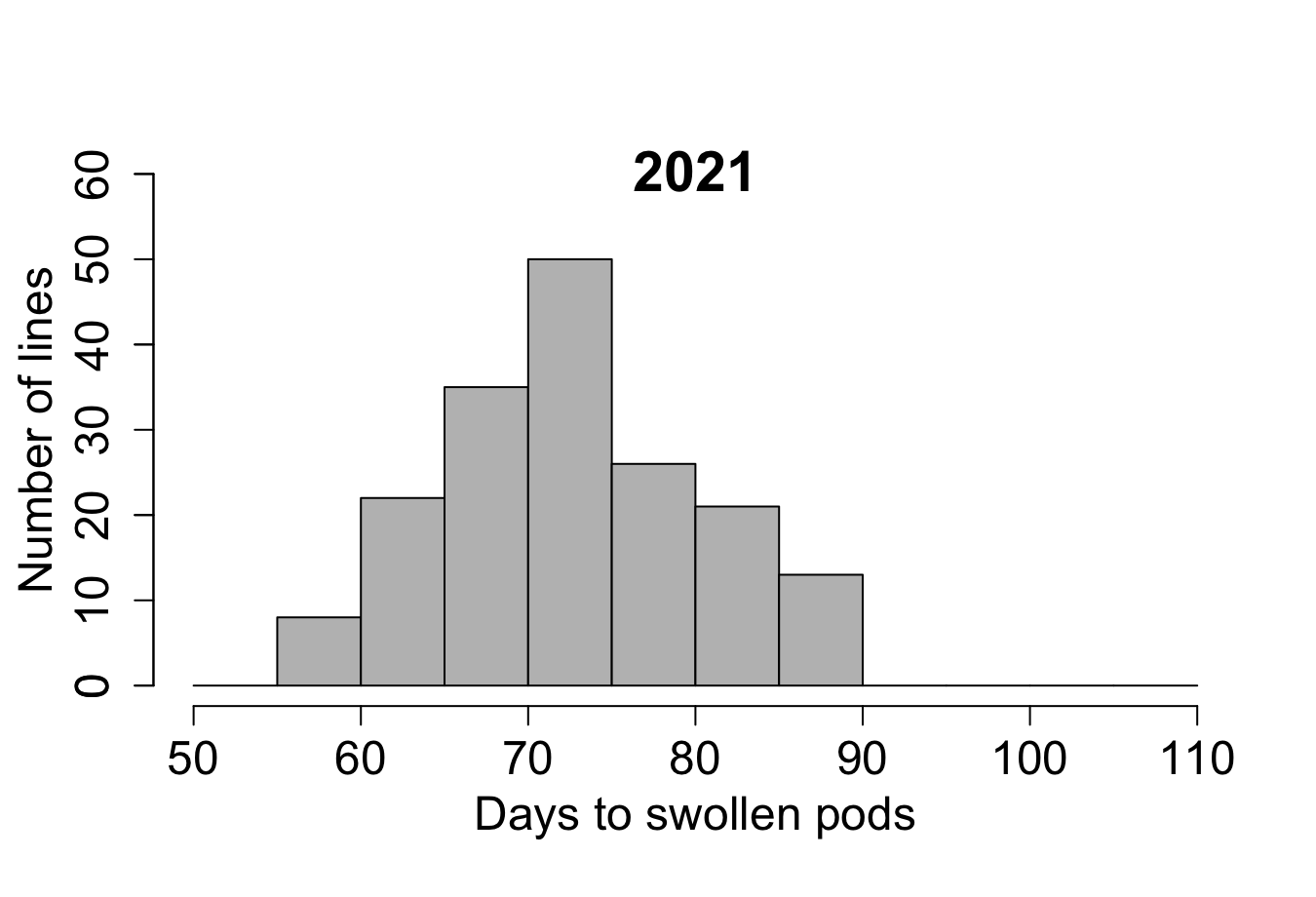

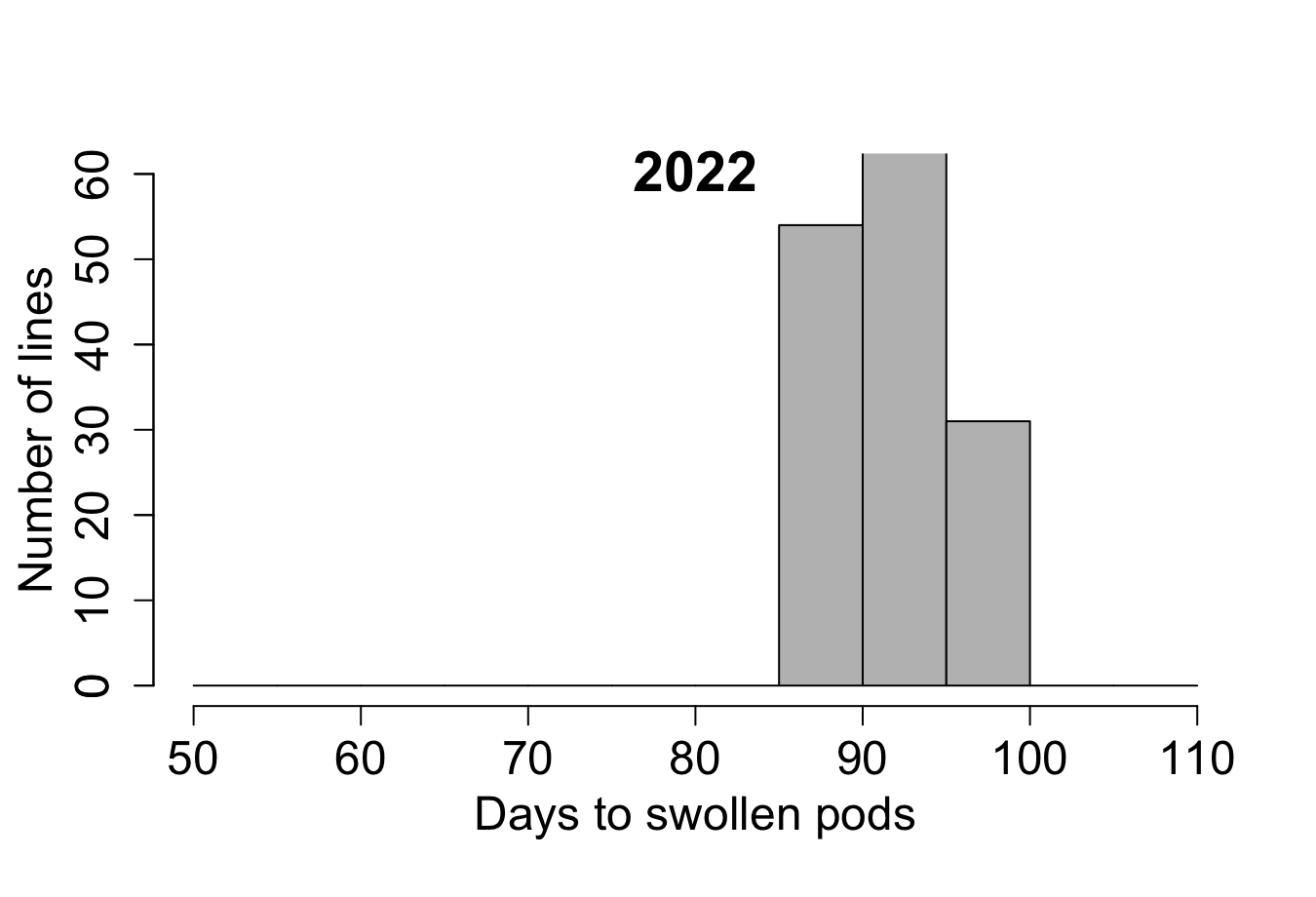
**

**
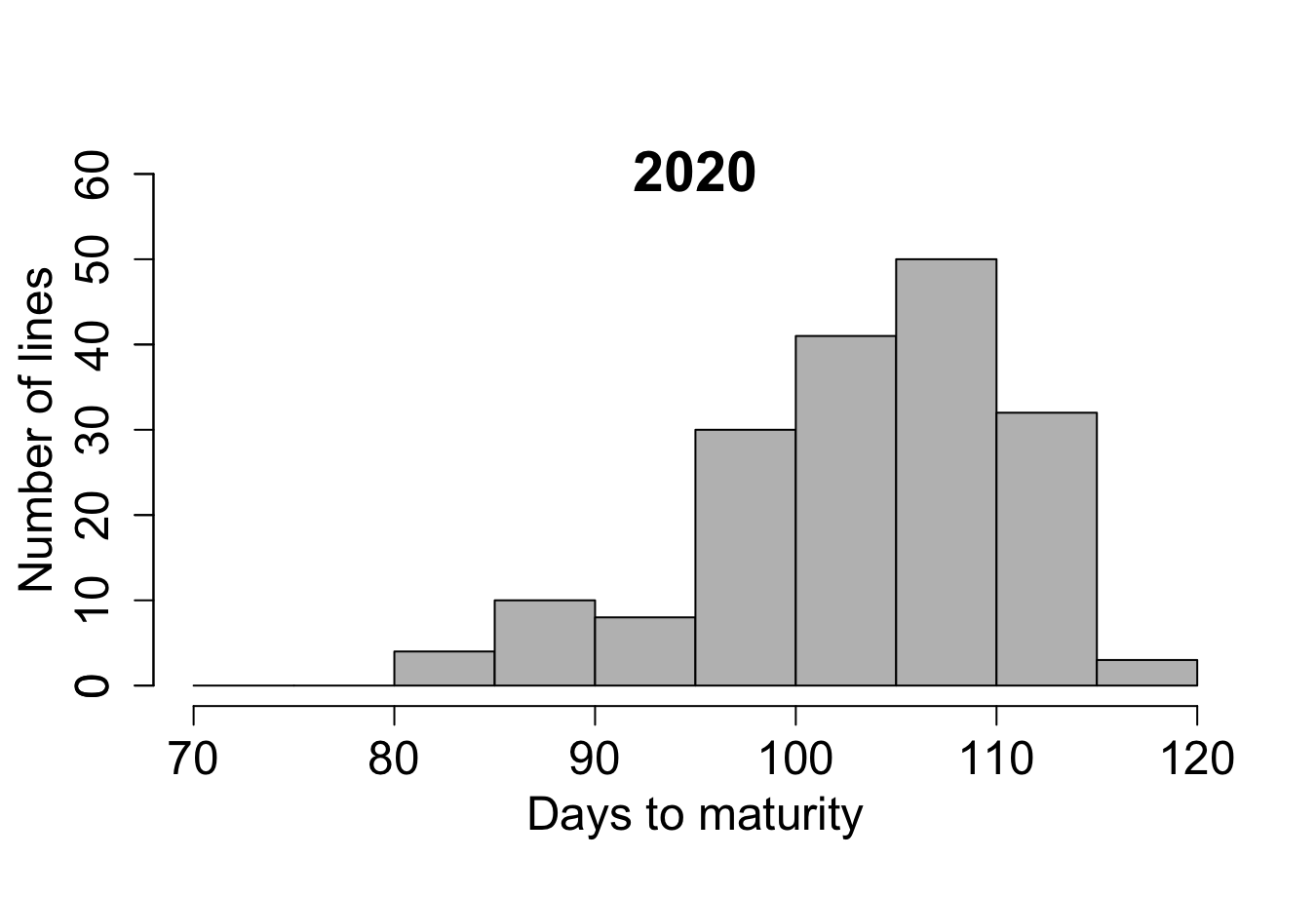

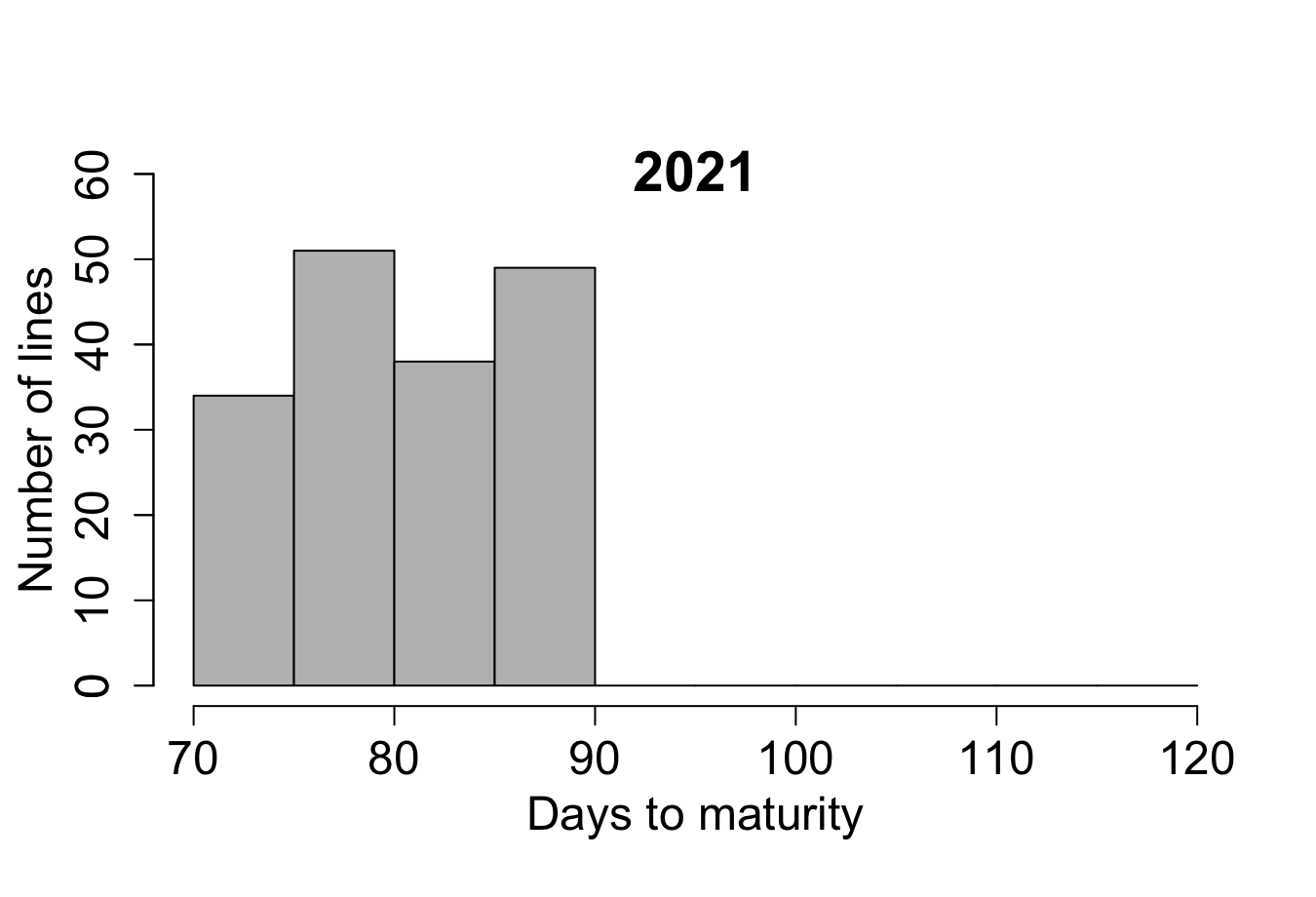

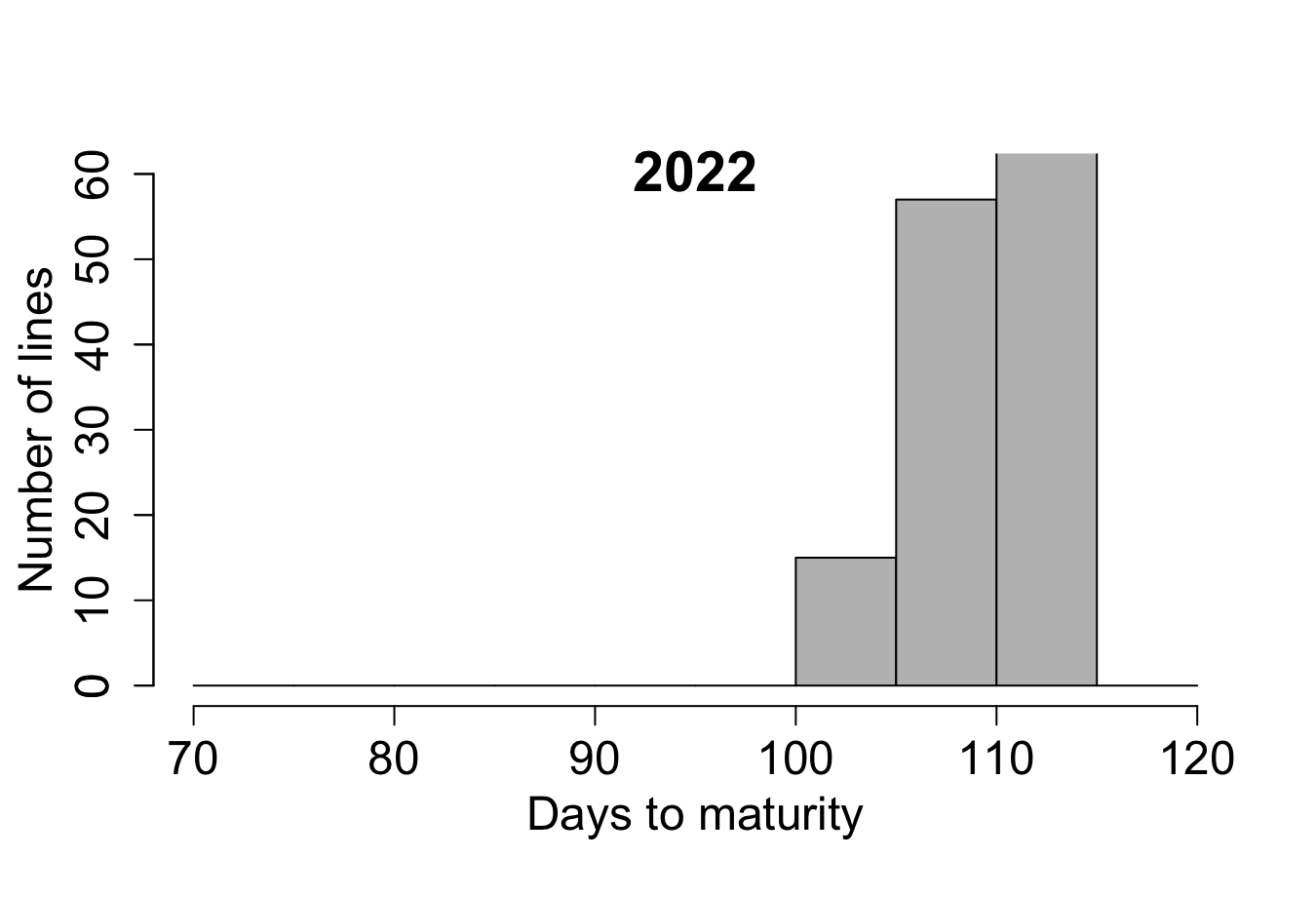
**

**
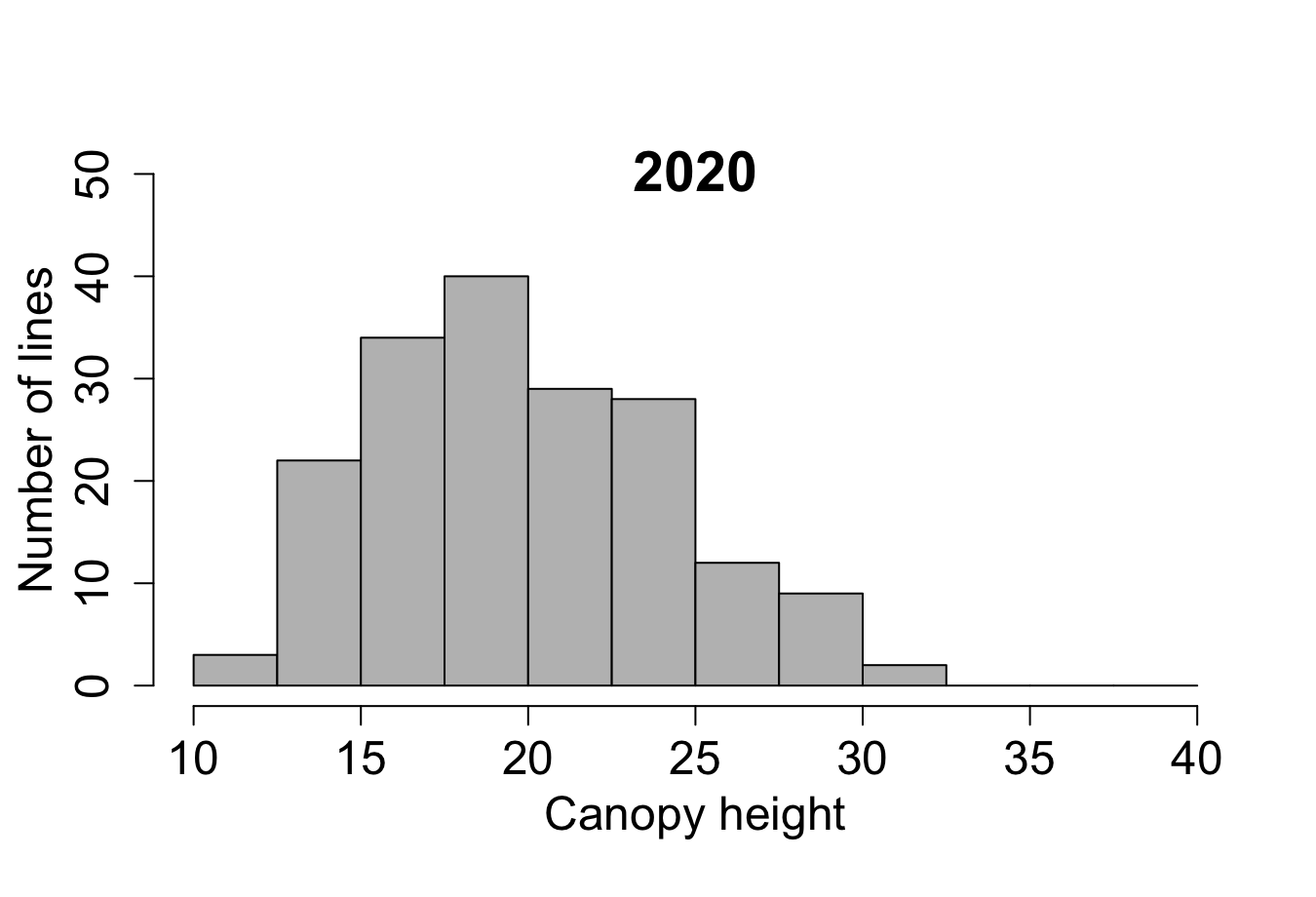

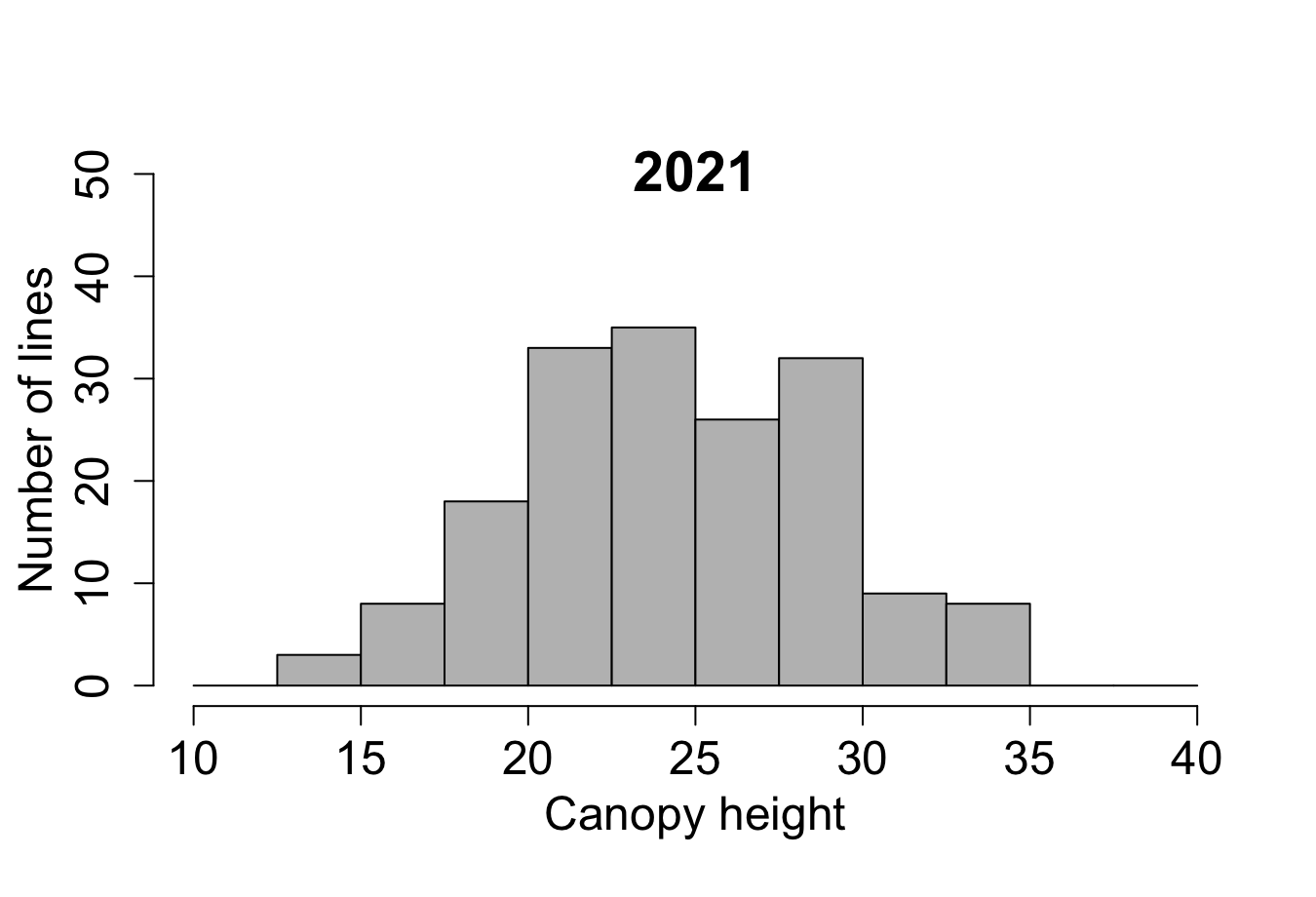

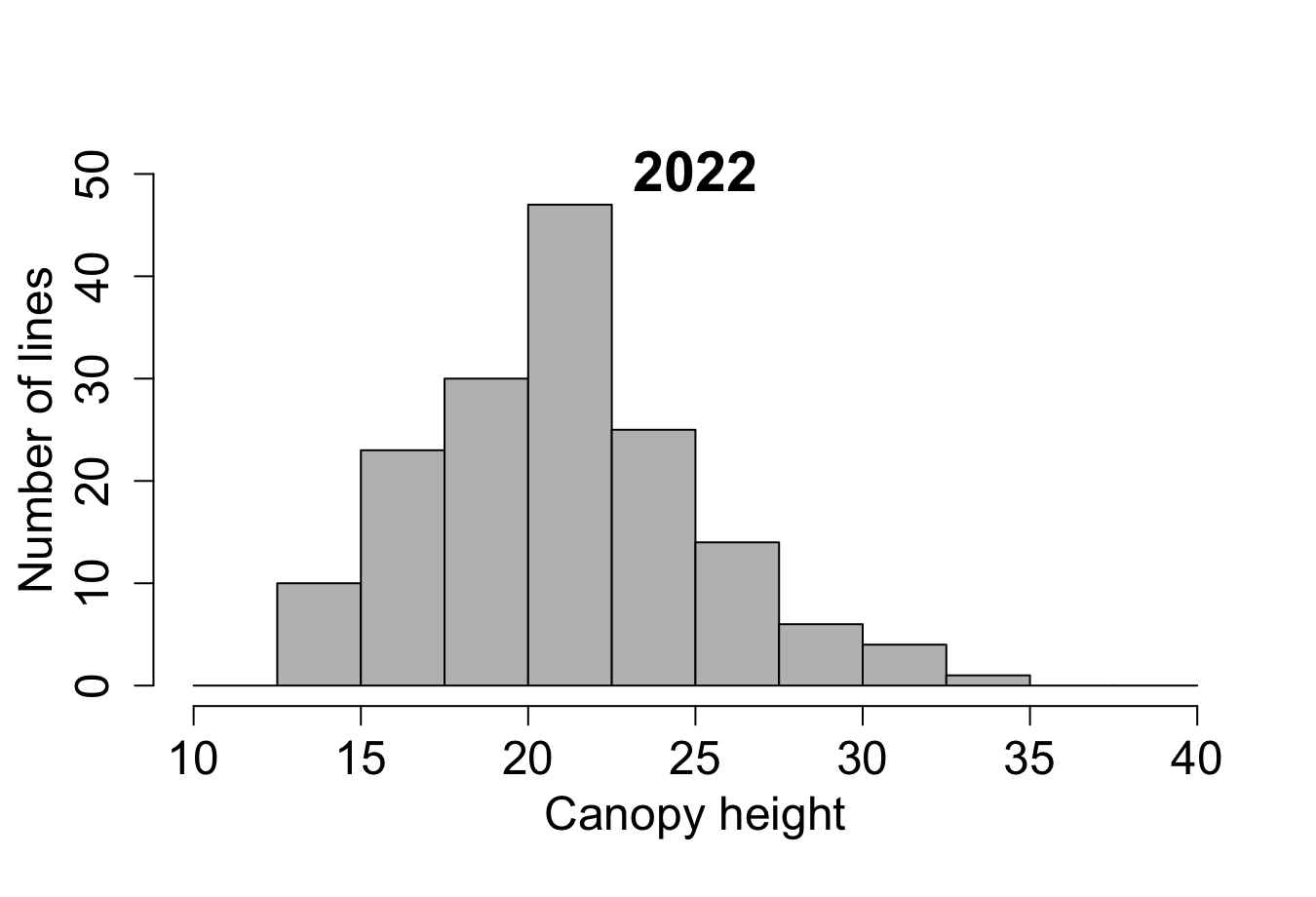
**

**
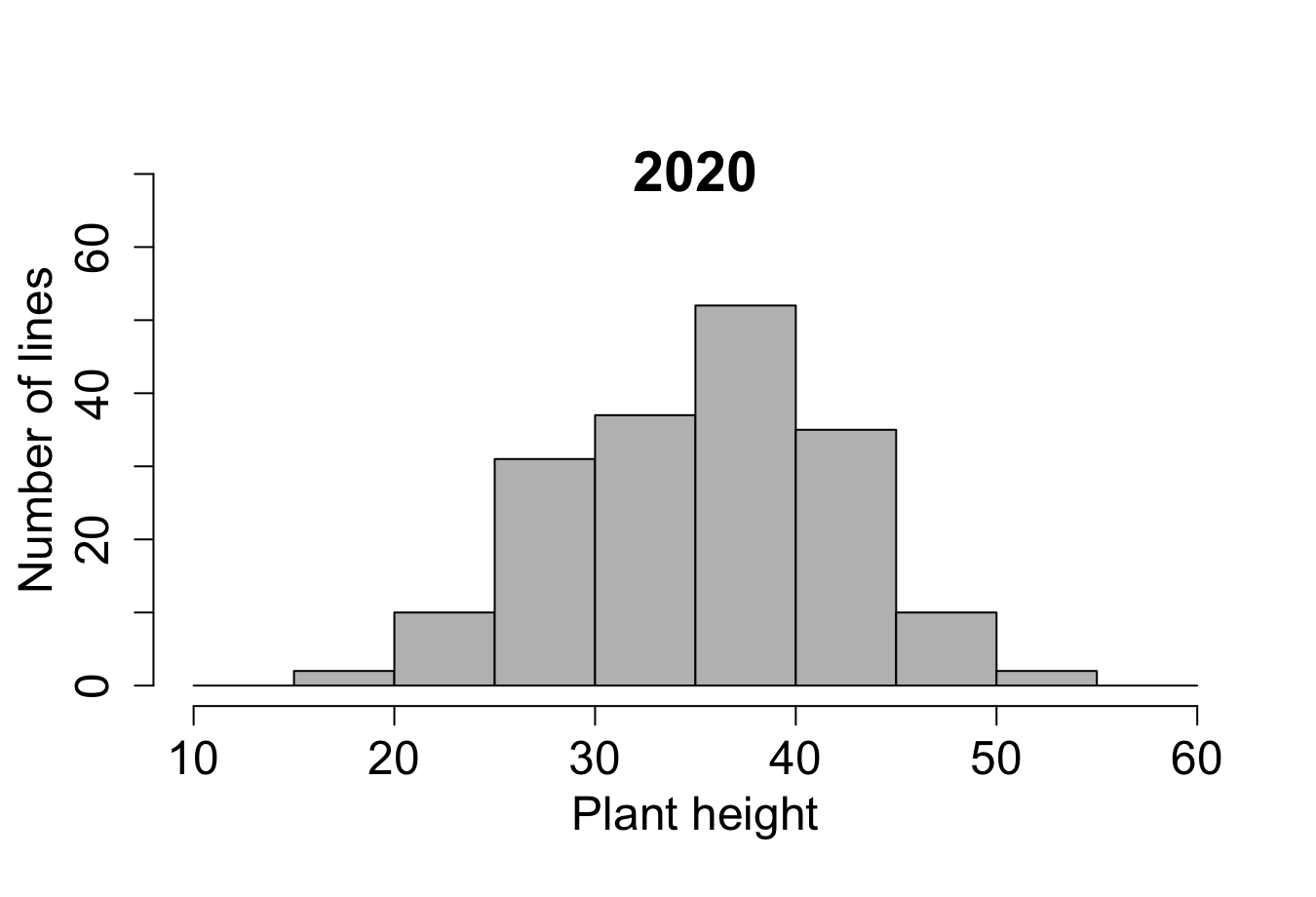

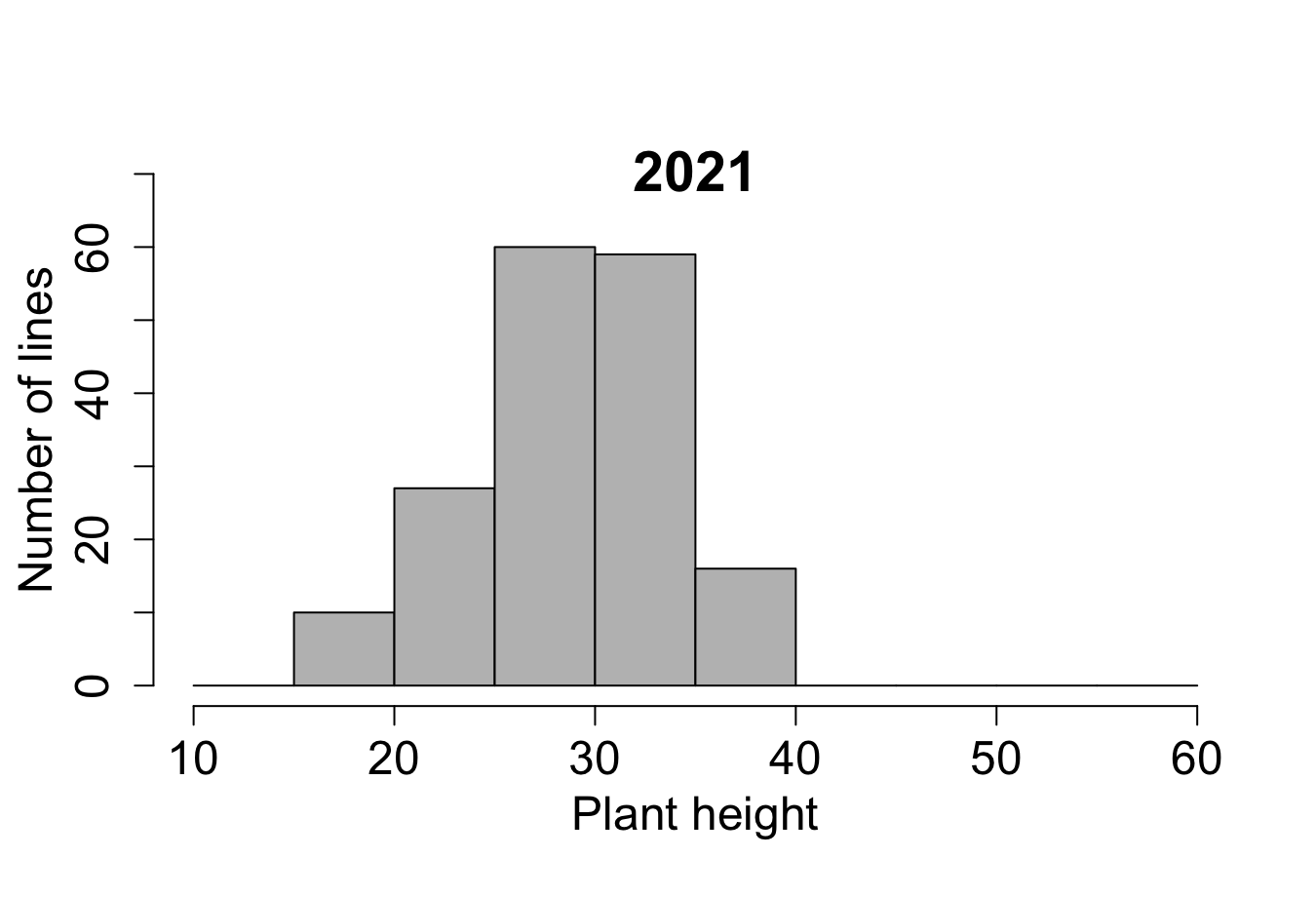

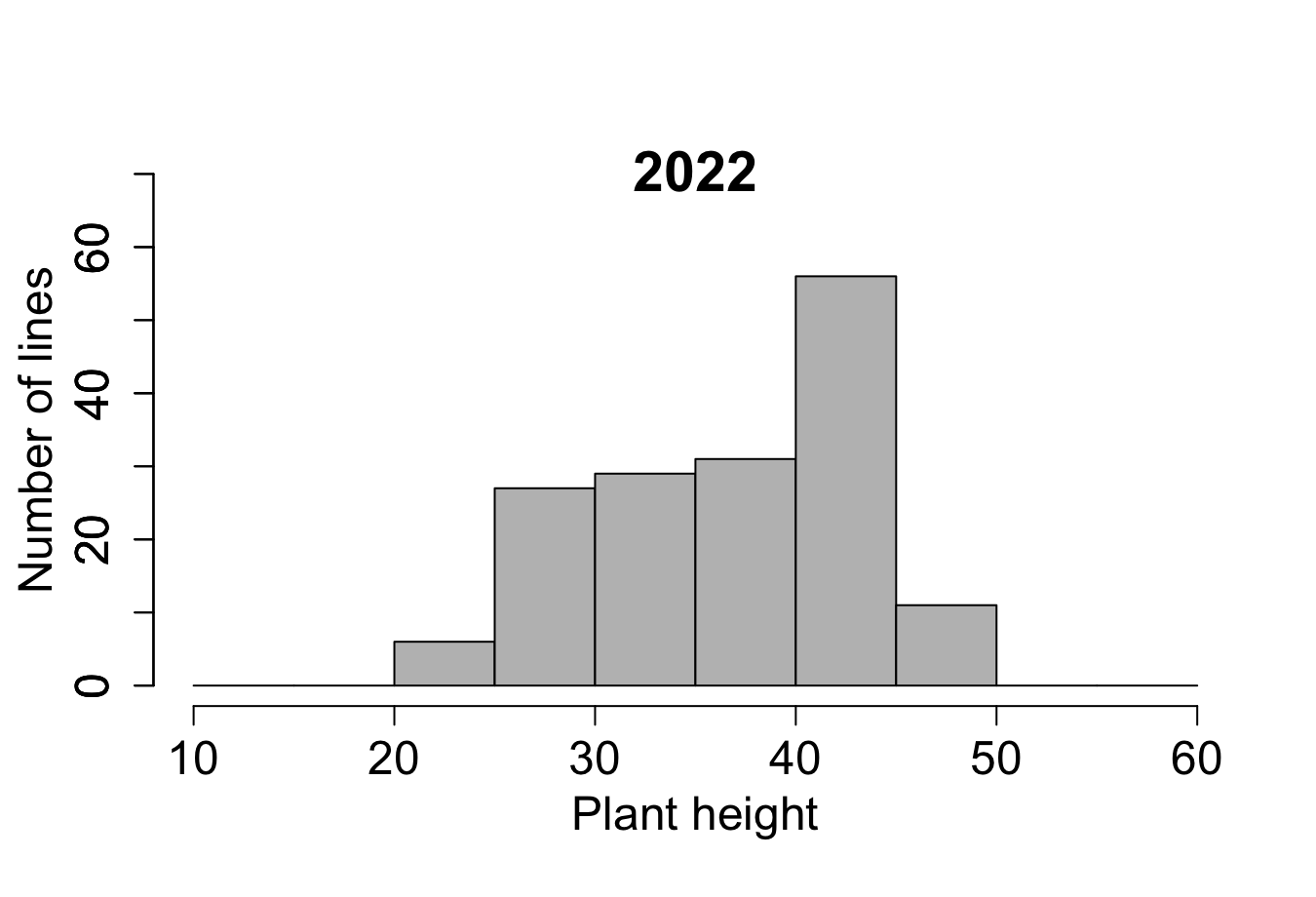
**

**Figure S24** Frequency distribution of phenotypic variation of 13 agronomic traits collected over three years for 183 *L. culinaris* landrace and cultivated accessions.

**Figure S25** Weather temperature comparison for April to August in 2020, 2021, and 2022

**Figure S26** Seed variation among different *Lens* groups (*L. nigricans*, *L. ervoides*, *L. odemensis*, *L. orentalis*, *L. culinaris* landrace, *L. culinaris* cultivated)
